# Synthesis of (*E*,*E*)-Dienones and (*E*,*E*)-Dienals via Palladium-Catalyzed *γ*,*δ*-Dehydrogenation of Enones and Enals

**DOI:** 10.1016/j.isci.2019.09.027

**Published:** 2019-09-21

**Authors:** Gao-Fei Pan, Xing-Long Zhang, Xue-Qing Zhu, Rui-Li Guo, Yong-Qiang Wang

**Affiliations:** 1 Key Laboratory of Synthetic and Natural Functional Molecule Chemistry of Ministry of Education, College of Chemistry & Materials Science, Northwest University, Xi'an 710069, P. R. China

**Keywords:** Catalysis, Organic Synthesis, Chemical Compounds in Materials Science

## Abstract

A new strategy for the synthesis of conjugated (*E*,*E*)-dienones and (*E*,*E*)-dienals via a palladium-catalyzed aerobic *γ*,*δ*-dehydrogenation of enones and enals has been developed. The method can be employed in the direct and efficient synthesis of various (*E*,*E*)-dienones and (*E*,*E*)-dienals, including non-substituted *α*-, *β*-, and *γ*- and/or *δ*-substituted (*E*,*E*)-dienones and (*E*,*E*)-dienals. The protocol is featured by the ready accessibility and elaboration of the starting materials, good functional group compatibility, and mild reaction conditions. Furthermore, the reaction is of complete *E*,*E*-stereoselectivity and uses molecular oxygen as the sole clean oxidant.

## Highlights

• Good compatibility of various substitutions
• Complete *E*,*E*-stereoselectivity
• Ready accessibility of the starting materials
• Oxygen as the sole oxidant

## Graphical Abstract

**Figure:**
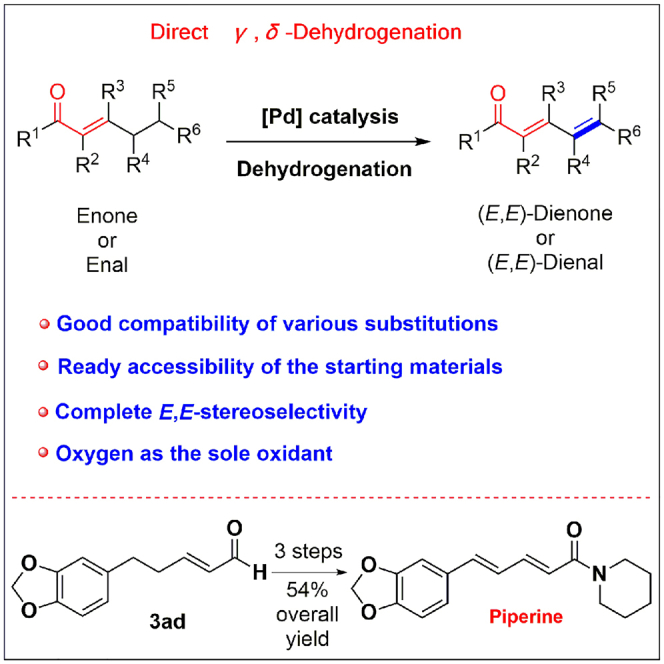

## Introduction

**Scheme 1:**
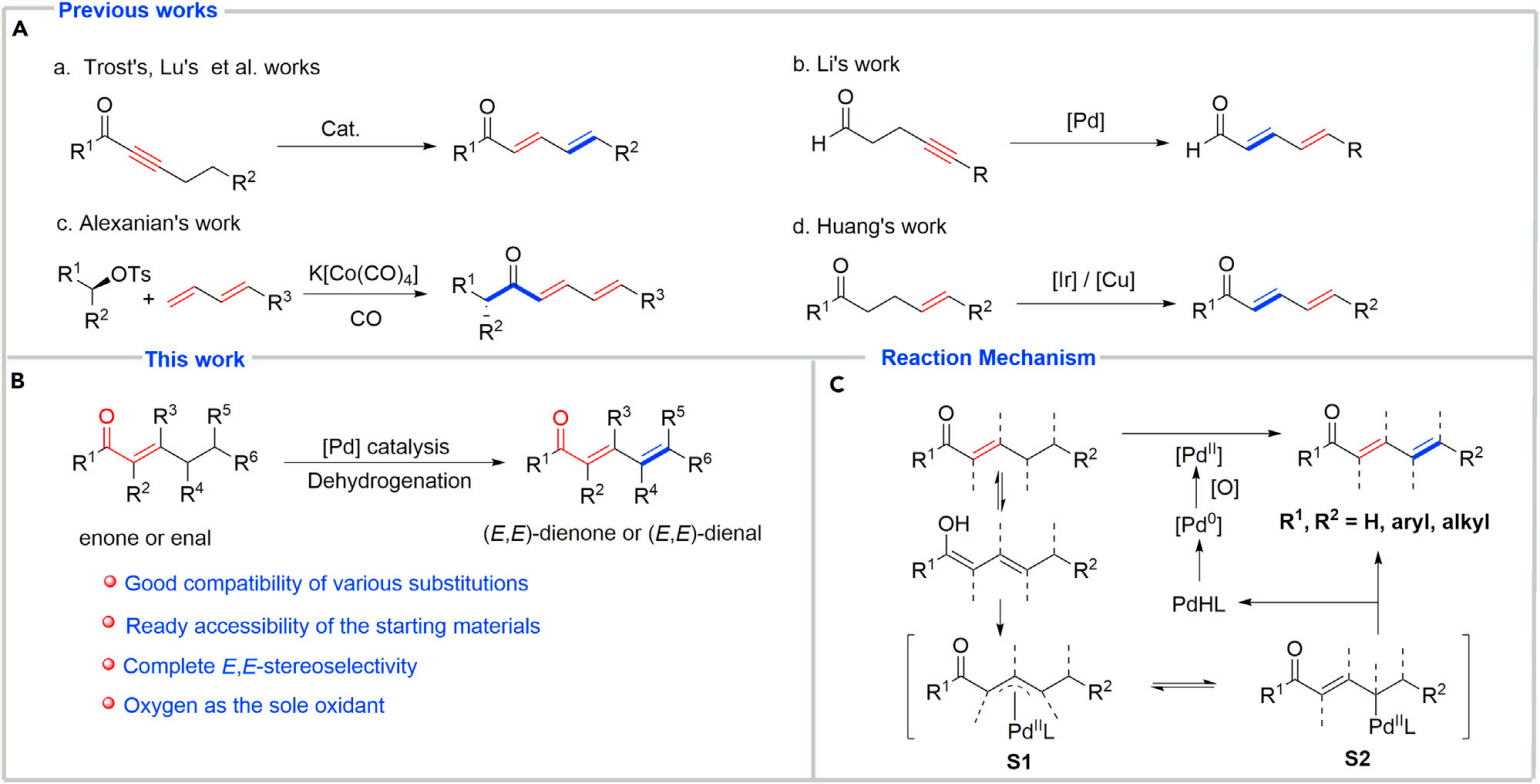
Strategies for Synthesis of (*E*,*E*)-Dienones and (*E*,*E*)-Dienals (A) Previous work for synthesis of (*E*,*E*)-dienones and (*E*,*E*)-dienals. (B) Our work for synthesis of (*E*,*E*)-dienones and (*E*,*E*)-dienals. (C) Reaction mechanism for the palladium-catalyzed γ,δ-dehydrogenation of enones and enals.

(*E*,*E*)-*α*,*β*,*γ*,*δ*-unsaturated carbonyl structural motifs are prevalent in natural products, drug molecules, and functional organic materials (Harned and Volp, 2011, Woerly et al., 2014). Conjugated dienones and dienals are also versatile precursors for 1,2- (Zhang and Morken, 2009), 1,4- (Csákÿ et al., 2010, Amoah and Dieter, 2017), or 1,6-addition (Poulsen et al., 2015, den Hartog et al., 2015, Caruana et al., 2014, Shaw and White, 2015, Gu et al., 2015); Diels-Alder reaction (Xiong et al., 2012, Li et al., 2012, Tian et al., 2014); cycloaddition (Horie et al., 2011, Albrecht et al., 2012); and other transformations (Meisner et al., 2012, Bos et al., 2013). Traditionally, the approaches to access conjugated dienones or dienals involve Knoevenagel condensation (He et al., 2011), Wittig-Horner reaction (An et al., 2015, Poulsen et al., 2016), Claisen rearrangement (Cookson and Gopalan, 1978, Motika et al., 2015), and addition-elimination reaction (Crouch et al., 2011, Yuan and Han, 2012, Kim and Oh, 2015, Li et al., 2019). These methods usually require basic conditions, which might be incompatible with the existing functional groups and/or the original stereochemistry. Moreover, these methods are often multistep sequences and suffer from low yields. In 1988, Trost's group (Trost and Schmidt, 1988, Trost and Kazmaier, 1992, Trost and Rudd, 2002, Trost and Rudd, 2005, Trost and Biannic, 2015) and Lu's group (Guo and Lu, 1993, Inoue and Imaizumi, 1988, Kwong et al., 2008, Lu et al., 2001, Ma et al., 1988, Ma et al., 1989) independently and virtually simultaneously developed the isomerization of alkynones to the corresponding conjugated dienones (Scheme 1A, a). Recently, Li's group reported a palladium-catalyzed isomerization of 4-alkynals to conjugated dienals (Scheme 1A, b) (Hearne and Li, 2017). In spite of the alkyne isomerization protocol being a great advance in view of the relatively mild reaction conditions, the inherent structural feature of alkyne prevents the method from the direct preparation of multi-substituted dienones and dienals. More recently, Alexanian et al. reported an elegant cobalt-catalyzed carbonylative cross-coupling of alkyl tosylates and dienes to synthesize conjugated dienones (Scheme 1A, c) (Sargent and Alexanian, 2017). Also, Huang et al. reported a great direct aerobic *α*,*β*-dehydrogenation of *γ*,*δ*-unsaturated amides and acids to produce conjugated dienamides and dienoic acids by an iridium/copper relay catalysis process (Scheme 1A, d) (Wang et al., 2018). Although these remarkable progresses have been made, significant challenges remain unaddressed, for example, limited substrate scope and tedious preparation of starting material. Therefore, new strategies for facile and efficient synthesis of conjugated dienones and dienals are still highly desirable.

Our group has long sought catalytic conditions for aerobic dehydrogenation reactions. We thought if dienones or dienals could be prepared by the aerobic *γ*,*δ*-dehydrogenation of enones or enals (Scheme 1B). This strategy has two advantages: (1) the precursors, enones or enals, can be obtained readily (some of them are commercially available and they also can be easily synthesized by aldol-like condensations, *α*-substitution of carbonyl compounds and subsequent elimination, oxidative *α*,*β*-dehydrogenation of saturated ketones or aldehydes, and so on) (Wade, 2005, Smith and March, 2001, Nicolaou et al., 2000, Nicolaou et al., 2002, Izawa et al., 2011, Diao and Stahl, 2011, Bigi and White, 2013, Huang and Dong, 2013, Deng et al., 2014, Huang et al., 2015, Jie et al., 2016, Yoshii et al., 2016, Chen et al., 2017) and (2) dienones and dienals bearing substituent groups in various positions could be produced directly. Despite these obvious benefits, to the best our knowledge, the efficient *γ*,*δ*-dehydrogenation of enones or enals to produce conjugated dienones or dienals has not been reported so far.

Mechanistically, we conceived that transition metal, especially palladium, could activate the allylic C–H bond to afford a *π*-allylpalladium intermediate (**S1**), which could generate a *γ*-palladation enone or enal (**S2**) (Patil and Yamamoto, 2006), which then underwent a sequence *β*-hydride elimination to give conjugated dienyl carbonyl product and Pd^II^-hydride intermediate that underwent reductive elimination and oxidation to complete the catalytic cycle (Scheme 1C). In this protocol, there were two challenges: one is the avoiding the direct oxidation of alkene bond of starting material (e.g., Wacker-type oxidation) and the other is preventing the product from the deeper oxidation (e.g., to generate trienone). To address the challenges, an efficient but mild catalytic oxidative system should be developed.

## Results and Discussion

To test this proposal, we chose enone (**1aa**) as the model substrate to begin our investigation. Initially, various palladium catalysts were examined with DMSO as the solvent and molecular oxygen as the terminal oxidant (Table 1, entries 1–7). Pd(TFA)_2_, Pd(OAc)_2_, Pd(PPh_3_)_4_, and Pd_2_(dba)_3_ reaction systems afforded the desired product **2aa** in 38%, 23%, 25%, and 18% yields, respectively, whereas PdCl_2_ and Pd(PPh_3_)_2_Cl_2_ systems could not react and Pd(OH)_2_ reaction system only provided trace **2aa**. Considering that trifluoroacetic acid (TFA) and Pd(OAc)_2_ can generate more electropositive [Pd(II)O_2_CCF_3_]^+^ species *in situ* (Lu et al., 1999, Jia et al., 2000), which is predictably easier to form *π*-allylpalladium intermediate (Scheme 1C, **S1**) and *γ*-palladation enone (Scheme 1C, **S2**), thereby facilitating the *γ*,*δ*-dehydrogenation reaction, 0.2 equiv. of TFA was introduced into Pd(OAc)_2_-catalyzed reaction system. To our delight, the reaction gave (*E*,*E*)-dienone **2aa** in 63% yield with complete double bond (*E*,*E*)-stereoselectivity (Table 1, entry 8). Then, 0.2 equiv. TFA was added into other palladium-catalyzed reaction systems. Interestingly, the yields of most of the reactions were improved to a certain extent; nevertheless, the result of the combination of Pd(OAc)_2_ and TFA was still the better (Table 1, entries 9–14). Next, the solvent was screened, and DMSO proved to be the best solvent (Table 1, entries 15–17). After careful investigation of the amount of TFA, 2.0 equiv. TFA provided the highest yield (Table 1, entry 18). Replacing TFA with other acids proved to be either less effective or totally ineffective (Table 1, entry 19). Thus the optimized reaction conditions for the *γ*,*δ*-dehydrogenation of **1aa** were identified as following: **1aa** (0.5 mmol), Pd(OAc)_2_ (10 mol%), and TFA (2.0 equiv.) under oxygen atmosphere in DMSO at 80°C.

**Table 1:** Optimization of the Reaction Conditions


Entry	Pd source	TFA (equiv.)	Solvent	Yield^a^ (%)
1	Pd(TFA)_2_	–	DMSO	38
2	Pd(OAc)_2_	–	DMSO	23
3	Pd(PPh_3_)_4_	–	DMSO	25
4	Pd_2_(dba)_3_	–	DMSO	18
5	PdCl_2_	–	DMSO	NR
6	Pd(PPh_3_)_2_Cl_2_	–	DMSO	NR
7	Pd(OH)_2_	–	DMSO	Trace
8	Pd(OAc)_2_	0.2	DMSO	63
9	Pd(TFA)_2_	0.2	DMSO	51
10	Pd(PPh_3_)_4_	0.2	DMSO	48
11	Pd_2_(dba)_3_	0.2	DMSO	56
12	PdCl_2_	0.2	DMSO	Trace
13	Pd(PPh_3_)_2_Cl_2_	0.2	DMSO	NR
14	Pd(OH)_2_	0.2	DMSO	59
15	Pd(OAc)_2_	0.2	DMF	25
16	Pd(OAc)_2_	0.2	CH_3_CN	50
17	Pd(OAc)_2_	0.2	THF	20
18^b^	Pd(OAc)_2_	2.0	DMSO	73
19^c^	Pd(OAc)_2_	2.0	DMSO	<13

Reaction conditions: Unless otherwise noted, the reaction was carried out with **1aa** (0.5 mmol), [Pd] (10 mol %) in solvent (2.5 mL) under O_2_ (1 atm) atmosphere at 80°C for 12 h.

a Isolated yield.

b Amount of TFA: 0.1 equiv. (30%), 0.2 equiv. (63%), 0.5 equiv. (66%), 1.0 equiv. (68%), 1.5 equiv. (70%), 3.0 equiv. (68%).

c Other acid (1.0 mL): for hydrochloric acid and benzoic acid, no product; AcOH and TsOH, trace product; CF_3_SO_3_H, 12% yield.

**Scheme 2:**
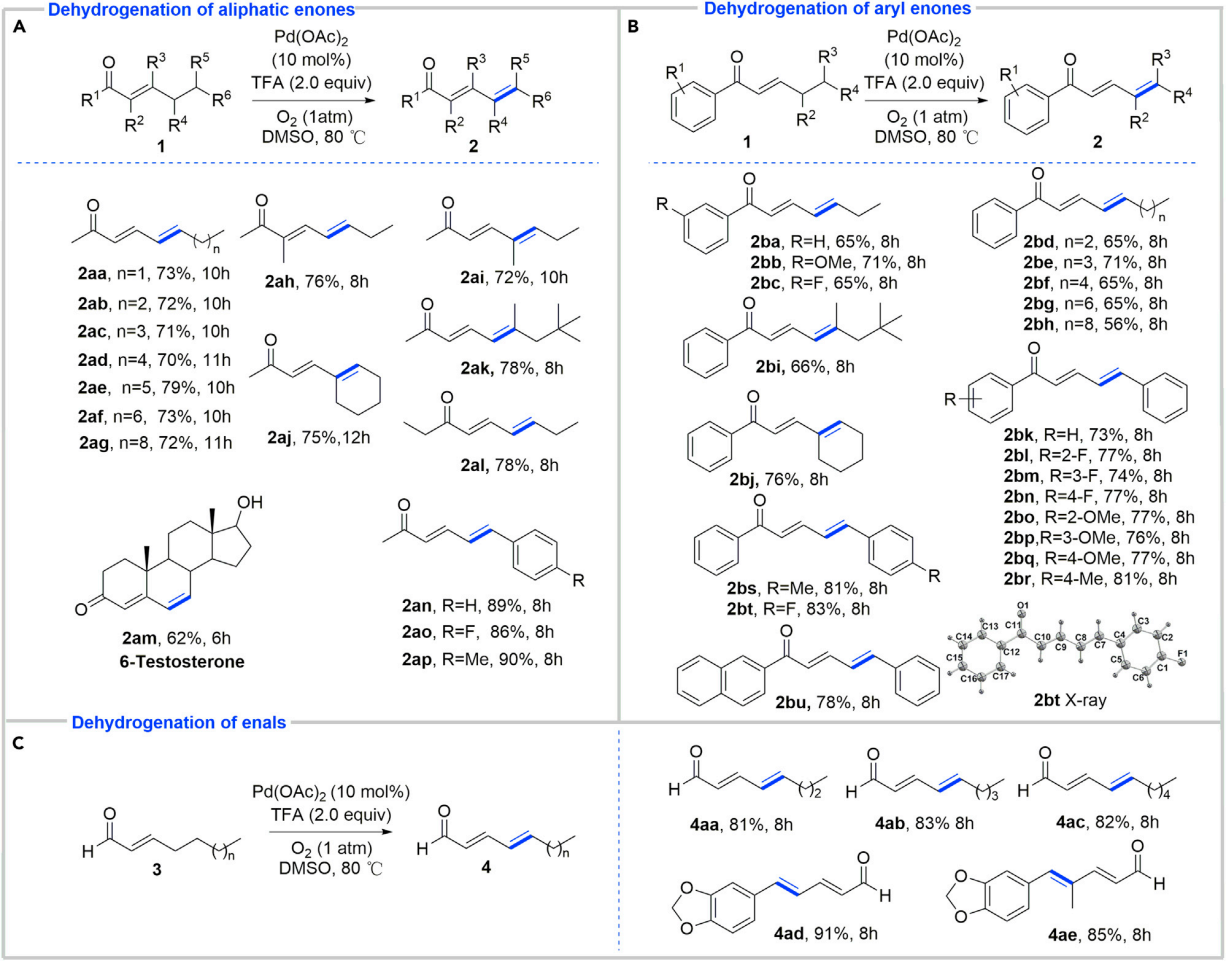
Substrate Scope of the Palladium-Catalyzed *γ*,*δ*-Dehydrogenation of Enones and Enals (A) Dehydrogenation of aliphatic enones. (B) Dehydrogenation of aryl enones. (C) Dehydrogenation of enals.

With the optimized reaction conditions in hand, we next surveyed the substrate scope (Scheme 2A). First, the length of carbon chain of enones was increased to check if further oxidation could happen. Delightedly, all of them only provided the desired (*E*,*E*)-dienones in 70%–79% yields and no further oxidative product (e.g., trienone) was observed (Scheme 2A, **2aa**-**2ag**). Substitutions at each position (i.e., *α*-, *β*-, *γ*-, or *δ*-positions or beyond), despite their increasing steric hindrance, were all well-tolerated (**2ah**-**2am**). Note that the *γ*,*δ*-dehydrogenation could occur not only on aliphatic chain but also on aliphatic cycles (**2aj**). Interestingly, a steroid compound **1am** also successfully underwent the *γ*,*δ*-dehydrogenation to give 6-testosterone (**2am**) in good yield. This case together with **2al** showed that the current catalytic reaction conditions preferred *γ*,*δ*-dehydrogenation to *α*,*β*-dehydrogenation, highlighting the advantage of the process for the synthesis of dienones. *δ-*Aryl-substituted enones could also be *γ,δ-*dehydrogenated in excellent yields (**2an**-**2ap**). It is noteworthy that, in all cases, only *E*,*E*-isomers were obtained, and no *Z*-isomers can be detected by analyzing the reaction mixtures.

Next, we investigated another kind of enones, aryl enones (Scheme 2B). 1-Arylhept-2-en-1-ones bearing either electron-donating or electron-withdrawing groups all reacted smoothly to provide the desired dienones in good yields (**2ba**-**2bc**). Increasing the length of the alkyl chain (**2bd**-**2bh**) or changing the straight chain to branched chain (**2bi**) or aliphatic cycle (**2bj**) was permitted. A series of substituted (*E*)-1,5-diphenylpent-2-en-1-ones (**1bk**-**1bt**) were also investigated. The results indicated that both the position (*o*-, *m*- or *p*-) and the electronic properties (electron-donating or electron-withdrawing property) of substitution groups did not affect the dehydrogenation and that they all afforded the corresponding (*E*,*E*)-dienones in 73%–81% yields. The other aromatic substrate, naphthyl enone, was also suitable for the reaction to give dienone **2bu** in good yields. Again, only *E*,*E*-isomers were obtained. The structure of **2bt** was confirmed by single-crystal X-ray diffraction (see Supplemental Information).

Then, we focused on the *γ*,*δ*-dehydrogenation of enals, which were challenging substrates due to the aldehyde's susceptibility toward oxidation under oxidative conditions (Padala and Jeganmohan, 2012, Liu et al., 2015, Santhoshkumar et al., 2015) and undesired metal insertion into an acyl C−H bond (Bosnich, 1998, Fristrup et al., 2008, Garralda, 2009, Jun et al., 2007, Leung and Krische, 2012, Modak et al., 2012, Murphy and Dong, 2014, Willis, 2010). Pleasingly, all enals worked well as enones to produce the desired (*E*,*E*)-dienals in good to excellent yields, and the susceptible aldehyde group remained intact, indicating that the oxidative dehydrogenation conditions were very mild (Scheme 2C). The reaction also only provided *E*,*E*-isomers, and no *Z*-isomers could be detected.

**Scheme 3:**
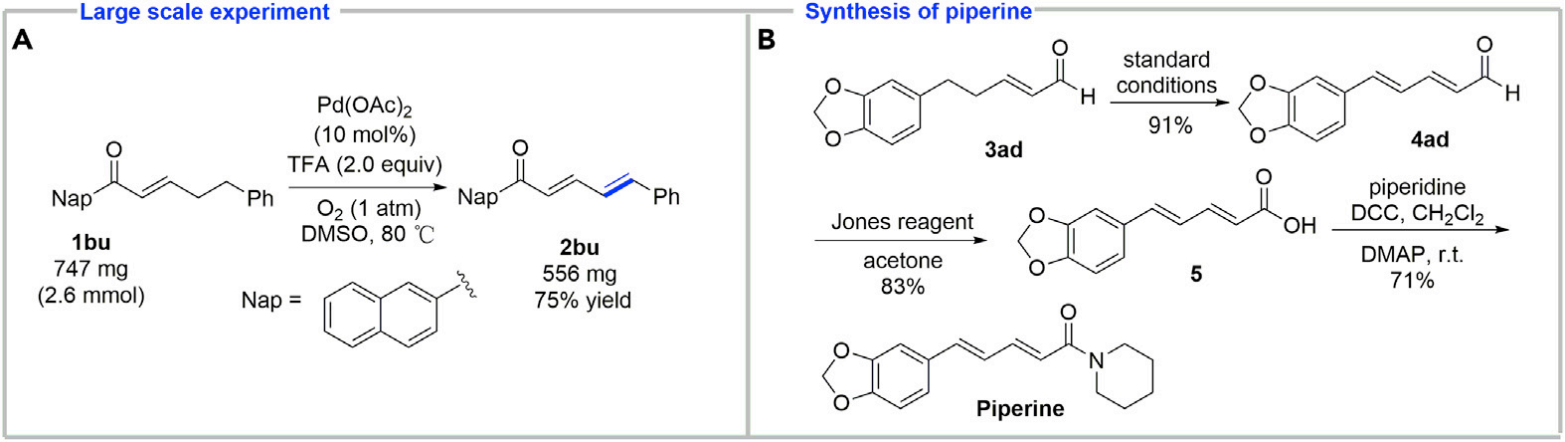
The Practicality of the Palladium-Catalyzed *γ*,*δ*-Dehydrogenation of Enones and Enals (A) Large-scale experiment. (B) Synthesis of piperine.

To test the practicality of the method, a large-scale experiment has been carried out. With the above-mentioned standard reaction conditions, **1bu** (747 mg, 2.6 mmol) was converted into the desired dienone **2bu** (556 mg) in 75% yield (Scheme 3A). Notably, when the catalyst loading was reduced to 6 mol %, the yield was not decreased, although more reaction time was required.

To highlight the synthetic utility of this methodology, we employed it as a key step to rapidly synthesize a natural product, piperine, an alkaloid responsible for the pungency of black pepper and long pepper. Recent investigations have shown that piperine has diverse bioactivities including chemopreventive, antioxidant, immunomodulatory, anticarcinogenic, stimulatory, hepatoprotective, anti-inflammatory, antimicrobial, and antiulcer activities (Doucette et al., 2013, Gorgani et al., 2017). Enal **3ad** was converted into (*E*,*E*)-dienal **4ad** under standard conditions, followed by oxidation with Jones reagent to acid and the condensation with piperidine to give piperine in three steps in 54% overall yield (Scheme 3B).

**Scheme 4:**
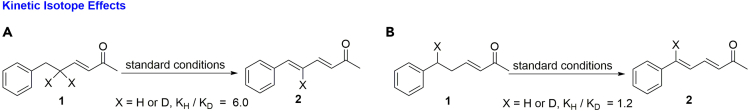
Kinetic Isotope Effect (A) The KIE value of two parallel competition reactions of **1an** and *γ*-deuterated [D_2_]-**1an**. (B) The intramolecular KIE value for the reaction of *δ*-deuterated [D]-**1an**.

To gain insight into the reaction mechanism, we carried out a series of kinetic isotope effect (KIE) experiments (Scheme 4). The KIE value of two parallel competition reactions of **1an** and *γ*-deuterated [D_2_]-**1an** was found to be 6.0 (Scheme 4A), and the intramolecular KIE value for the reaction of *δ-*deuterated [D]-**1an** was 1.2 (Scheme 4B). These results showed that the cleavage of the *γ*-C−H bond should be involved in the rate-determining step, whereas the elimination of *δ-*C−H bond was fast and not rate limiting. The complete *E*,*E*-stereoselectivity of dienones and dienals might be attributed to the formation of the thermodynamically more stable *E*-product at *β*-hydride elimination step in the current heating reaction conditions (80°C) and also to the probable presence of Pd-mediated isomerization of olefins under the current Pd-catalyzed reaction system (Bond and Hellier, 1965, Stang and White, 2011). In the reaction system, there were the nucleophilic TFA and H_2_O, which could react with *π*-allylpalladium intermediate (Chen and White, 2004, Chen et al., 2005), but we could not detect any corresponding product, which probably could be ascribed to the fast *β*-hydride elimination of *γ*-palladation **S2**, or the fast elimination of TFA or H_2_O of the corresponding –O_2_CCF_3_- or –OH-substituted products under the current acidic reaction conditions. In-depth studies are currently underway to fully elucidate the mechanistic details.

### Conclusion

In summary, we have developed a new strategy for the synthesis of conjugated (*E*,*E*)-dienones and (*E*,*E*)-dienals via a palladium-catalyzed aerobic *γ*,*δ*-dehydrogenation of enones and enals. Compared with the previous methods, the biggest advantage of the method is the generality. The method can be employed in the direct and efficient synthesis of various (*E*,*E*)-dienones and (*E*,*E*)-dienals, including non-substituted and *α*-, *β*-, *γ*-, and/or *δ*-substituted (*E*,*E*)-dienones and (*E*,*E*)-dienals. Another advantage of the method is the ready accessibility and elaboration of the starting materials, enones and enals, some of which are commercially available, and they also can be easily obtained by conventional approaches. Furthermore, the reaction is of complete *E*,*E*-stereoselectivity and uses molecular oxygen as the sole clean oxidant. Owing to mild reaction conditions and good functional group compatibility, the approach should have broad applications in organic synthesis, medical, and material chemistry.

### Limitations of the Study

*α*,*β*-Unsaturated amides, acids, and ester provided the *γ*,*δ*-dehydrogenated products in low yields under the current reaction conditions.

## Methods

All methods can be found in the accompanying Transparent Methods supplemental file.

## Data and Code Availability

The structures of **2bt** reported in this article have been deposited in the Cambridge Crystallographic Data Center under accession numbers CCDC: 1892057.

## Supplemental Information

Document S1. Transparent Methods and Figures S1–S110

## References

[bib1] Albrecht Ł., Dickmeiss G., Acosta F.C., Rodríguez-Escrich C., Davis R.L., Jørgensen K.A. (2012). Asymmetric organocatalytic formal [2+2]-cycloadditions via bifunctional H-bond directing dienamine catalysis. J. Am. Chem. Soc..

[bib2] Amoah E., Dieter R.K. (2017). Regioselective 1,4-conjugate addition of grignard reagents to *α*,*β-γ*,*δ*-DIenones and *α*,*β-γ*,*δ*-dienyl thiol esters. J. Org. Chem..

[bib3] An P., Xu N.-S., Zhang H.-L., Cao X.-P., Shi Z.-F., Wen W. (2015). Facile preparation of *α*-Cyano-*α*,*ω*-diaryloligovinylenes: a new class of color-tunable solid emitters. Chem. Asian J..

[bib4] Bigi M.A., White M.C. (2013). Terminal olefins to linear *α*,*β*-unsaturated ketones: Pd(II)/hypervalent iodine Co-catalyzed Wacker oxidation-dehydrogenation. J. Am. Chem. Soc..

[bib5] Bond G.C., Hellier M. (1965). Homogeneous catalysis by noble metal salts I. The homogeneous isomerization of olefins by palladium compounds. J. Catal..

[bib6] Bos P.H., Antalek M.T., Porco J.A., Stephenson C.R.J. (2013). Tandem dienone photorearrangement–cycloaddition for the rapid generation of molecular complexity. J. Am. Chem. Soc..

[bib7] Bosnich B. (1998). Asymmetric catalysis. A comparative study of the mechanisms of intramolecular hydroacylation and hydrosilation. Acc. Chem. Res..

[bib8] Caruana L., Kniep F., Johansen T.K., Poulsen P.H., Jørgensen K.A. (2014). A new organocatalytic concept for asymmetric *α*-alkylation of aldehydes. J. Am. Chem. Soc..

[bib9] Chen M.S., White M.C. (2004). A sulfoxide-promoted, catalytic method for the regioselective synthesis of allylic acetates from monosubstituted olefins via C-H oxidation. J. Am. Chem. Soc..

[bib10] Chen M.S., Prabagaran N., Labenz N.A., White M.C. (2005). Serial ligand catalysis: a highly selective allylic C-H oxidation. J. Am. Chem. Soc..

[bib11] Chen Y., Huang D., Zhao Y., Newhouse T.R. (2017). Allyl-palladium-catalyzed ketone dehydrogenation enables telescoping with enone *α*,*β*-vicinal difunctionalization. Angew. Chem. Int. Ed..

[bib12] Cookson R.C., Gopalan R. (1978). A new synthesis of conjugated dienones. J. Chem. Soc. Chem. Commun..

[bib13] Crouch I.T., Dreier T.D., Frantz D.E. (2011). Palladium-catalyzed elimination/isomerization of enol triflates into 1,3-dienes. Angew. Chem. Int. Ed..

[bib14] Csákÿ A.G., de la Herrán G., Murcia M.C. (2010). Conjugate addition reactions of carbon nucleophiles to electron-deficient dienes. Chem. Soc. Rev..

[bib15] Deng Y., Gong W., He J., Yu J.-Q. (2014). Ligand-enabled triple C-H activation reactions: one-pot synthesis of diverse 4-Aryl-2-quinolinones from propionamides. Angew. Chem. Int. Ed..

[bib16] Diao T., Stahl S.S. (2011). Synthesis of cyclic enones via direct palladium-catalyzed aerobic dehydrogenation of ketones. J. Am. Chem. Soc..

[bib17] Doucette C.D., Hilchie A.L., Liwski R., Hoskin D.W. (2013). Piperine, a dietary phytochemical, inhibits angiogenesis. J. Nutr. Biochem..

[bib18] Fristrup P., Kreis M., Palmelund A., Norrby P.-O., Madsen R. (2008). The mechanism for the rhodium-catalyzed decarbonylation of aldehydes: a combined experimental and theoretical study. J. Am. Chem. Soc..

[bib19] Garralda M.A. (2009). Aldehyde C-H activation with late transition metal organometallic compounds. Formation and reactivity of acyl hydrido complexes. Dalton Trans..

[bib20] Gorgani L., Mohammadi M., Najafpour G.D., Nikzad M. (2017). Piperine-the bioactive compound of black pepper: from isolation to medicinal formulations. Compr. Rev. Food Sci. Food Saf..

[bib21] Gu X., Guo T., Dai Y., Franchino A., Fei J., Zou C., Dixon D.J., Ye J. (2015). Direct catalytic asymmetric doubly vinylogous michael addition of *α*,*β*-unsaturated *γ*-butyrolactams to dienones. Angew. Chem. Int. Ed..

[bib22] Guo C., Lu X. (1993). Reinvestigation on the catalytic isomerisation of carbon-carbon triple bonds. J. Chem. Soc. Perkin Trans..

[bib23] Harned A.M., Volp K.A. (2011). The Sorbicillinoid family of natural products: isolation, biosynthesis, and synthetic studies. Nat. Prod. Rep..

[bib24] den Hartog T., Huang Y., Fañanás-Mastral M., Meuwese A., Rudolph A., Pérez M., Minnaard A.J., Feringa B.L. (2015). On the mechanism of Cu-catalyzed enantioselective extended conjugate additions: a structure-based approach. ACS Catal..

[bib25] He Y.-H., Hu Y., Guan Z. (2011). Natural *α*-amino acid L-lysine–catalyzed Knoevenagel condensations of *α*,*β*-unsaturated aldehydes and 1,3-dicarbonyl compounds. Synth. Commun..

[bib26] Hearne Z., Li C.-J. (2017). Palladium-catalysed atom-economical synthesis of conjugated dienals from terminal acetylenes and acrolein. Chem. Commun. (Camb.).

[bib27] Horie H., Kurahashi T., Matsubara S. (2011). Nickel-catalyzed cycloaddition of *α*,*β*,*γ*,*δ*-unsaturated ketones with alkynes. Angew. Chem. Int. Ed..

[bib28] Huang Z., Dong G. (2013). Catalytic direct *β*-arylation of simple ketones with aryl iodides. J. Am. Chem. Soc..

[bib29] Huang Z., Sam Q.P., Dong G. (2015). Palladium-catalyzed direct β-arylation of ketones with diaryliodonium salts: a stoichiometric heavy metal-free and user-friendly approach. Chem. Sci..

[bib30] Inoue Y., Imaizumi S. (1988). Catalytic formation of conjugated dienones from ynones by ruthenium complex. J. Mol. Catal..

[bib31] Izawa Y., Pun D., Stahl S.S. (2011). Palladium-catalyzed aerobic dehydrogenation of substituted cyclohexanones to phenols. Science.

[bib32] Jia C., Piao D., Lu W., Oyamada J., Kitamura T., Fujiwara Y. (2000). Efficient activation of aromatic C-H bonds for addition to C-C multiple bonds. Science.

[bib33] Jie X., Shang Y., Zhang X., Su W. (2016). Cu-catalyzed sequential dehydrogenation conjugate addition for *β*-functionalization of saturated ketones: scope and mechanism. J. Am. Chem. Soc..

[bib34] Jun C.-H., Jo E.-A., Park J.-W. (2007). Intermolecular hydroacylation by transition-metal complexes. Eur. J. Org. Chem..

[bib35] Kim H.Y., Oh K. (2015). 1,3-Dienones and 2H-Pyran-2-ones from soft *α*-vinyl enolization of *β*-chlorovinyl ketones: defined roles of Brönsted and Lewis base. Org. Lett..

[bib36] Kwong C.K.-W., Fu M.Y., Lam C.S.-L., Toy P.H. (2008). The phosphine-catalyzed alkyne to 1,3-diene isomerization reaction. Synthesis.

[bib37] Leung J.C., Krische M.J. (2012). Catalytic intermolecular hydroacylation of c-c *π*-bonds in the absence of chelation assistance. Chem. Sci..

[bib38] Li J.-L., Liu T.-Y., Chen Y.-C. (2012). Aminocatalytic asymmetric diels-alder reactions via HOMO activation. Acc. Chem. Res..

[bib39] Li C., Li M., Zhong W., Jin Y., Li J., Wu W., Jiang H. (2019). Palladium-catalyzed oxidative allylation of sulfoxonium ylides: regioselective synthesis of conjugated dienones. Org. Lett..

[bib40] Liu X., Li X., Liu H., Guo Q., Lan J., Wang R., You J. (2015). Aldehyde as a traceless directing group for Rh(III)-Catalyzed C-H activation: a facile access to diverse indolo[1,2-α]quinolines. Org. Lett..

[bib41] Lu W., Yamaoka Y., Taniguchi Y., Kitamura T., Takaki K., Fujiwara Y. (1999). Palladium(II)-Catalyzed carboxylation of benzene and other aromatic compounds with carbon monoxide under very mild conditions. J. Organomet. Chem..

[bib42] Lu X., Zhang C., Xu Z. (2001). Reactions of electron-deficient alkynes and allenes under phosphine catalysis. Acc. Chem. Res..

[bib43] Ma D., Lin Y., Lu X., Yu Y. (1988). A novel stereoselective synthesis of conjugated dienones. Tetrahedron Lett..

[bib44] Ma D., Yu Y., Lu X. (1989). Highly stereoselective isomerization of Ynones to conjugated dienones catalyzed by transition-metal complexes. J. Org. Chem..

[bib45] Meisner J.S., Sedbrook D.F., Krikorian M., Chen J., Sattler A., Carnes M.E., Murray C.B., Steigerwald M., Nuckolls C. (2012). Functionalizing molecular wires: a tunable class of *α*,*ω*-Diphenyl-*μ*,*ν*-Dicyano-Oligoenes. Chem. Sci..

[bib46] Modak A., Deb A., Patra T., Rana S., Maity S., Maiti D. (2012). A general and efficient aldehyde decarbonylation reaction by using a palladium catalyst. Chem. Commun. (Camb.).

[bib47] Motika S.E., Wang Q., Ye X., Shi X. (2015). Ambient synthesis of dienals via triazole-gold and amine catalysis relay. Org. Lett..

[bib48] Murphy S.K., Dong V.M. (2014). Enantioselective hydroacylation of olefins with rhodium catalysts. Chem. Commun. (Camb.).

[bib49] Nicolaou K.C., Zhong Y.-L., Baran P.S. (2000). A new method for the one-step synthesis of *α*,*β*-unsaturated carbonyl systems from saturated alcohols and carbonyl compounds. J. Am. Chem. Soc..

[bib50] Nicolaou K., Montagnon C.T., Baran P.S. (2002). Modulation of the reactivity profile of IBX by ligand complexation: ambient temperature dehydrogenation of aldehydes and ketones to *α*,*β*-unsaturated carbonyl compounds. Angew. Chem. Int. Ed..

[bib51] Padala K., Jeganmohan M. (2012). Highly regio- and stereoselective ruthenium(II)-Catalyzed direct *ortho*-alkenylation of aromatic and heteroaromatic aldehydes with activated alkenes under open atmosphere. Org. Lett..

[bib52] Patil N.T., Yamamoto Y. (2006). Palladium catalyzed cascade reactions involving *π*-allyl palladium chemistry. Top. Organomet. Chem..

[bib53] Poulsen P.H., Feu K.S., Paz B.M., Jensen F., Jørgensen K.A. (2015). Organocatalytic asymmetric 1,6-addition/1,4-addition sequence to 2,4-dienals for the synthesis of chiral chromans. Angew. Chem. Int. Ed..

[bib54] Poulsen P.H., Vergura S., Monleón A., Jørgensen D.K.B., Jørgensen K.A. (2016). Controlling asymmetric remote and cascade 1,3-dipolar cycloaddition reactions by organocatalysis. J. Am. Chem. Soc..

[bib55] Santhoshkumar R., Mannathan S., Cheng C.H. (2015). Ligand-controlled divergent C-H functionalization of aldehydes with enynes by cobalt catalysts. J. Am. Chem. Soc..

[bib56] Sargent B.T., Alexanian E.J. (2017). Cobalt-catalyzed carbonylative cross-coupling of alkyl tosylates and dienes: stereospecific synthesis of dienones at low pressure. J. Am. Chem. Soc..

[bib57] Shaw S., White J.D. (2015). Regioselective and enantioselective addition of sulfur nucleophiles to acyclic *α*,*β*,*γ*,*δ*-unsaturated dienones catalyzed by an iron(III)-salen complex. Org. Lett..

[bib58] Smith M.B., March J. (2001). Advanced Organic Chemistry.

[bib59] Stang E.M., White M.C. (2011). Molecular complexity via C-H activation: a dehydrogenative diels-alder reaction. J. Am. Chem. Soc..

[bib60] Tian X., Hofmann N., Melchiorre P. (2014). Asymmetric vinylogous diels-alder reactions catalyzed by a chiral phosphoric acid. Angew. Chem. Int. Ed..

[bib61] Trost B.M., Biannic B. (2015). Redox cycloisomerization approach to 1,2-dihydropyridines. Org. Lett..

[bib62] Trost B.M., Kazmaier U. (1992). Internal redox catalyzed by triphenylphosphine. J. Am. Chem. Soc..

[bib63] Trost B.M., Rudd M.T. (2002). An unusual ruthenium-catalyzed cycloisomerization of alkynes and propargyl alcohols. J. Am. Chem. Soc..

[bib64] Trost B.M., Rudd M.T. (2005). Ruthenium-catalyzed cycloisomerizations of diynols. J. Am. Chem. Soc..

[bib65] Trost B.M., Schmidt T. (1988). A simple synthesis of dienones via isomerization of alkynones effected by palladium catalysts. J. Am. Chem. Soc..

[bib66] Wade L.G. (2005). Organic Chemistry.

[bib67] Wang Z., He Z., Zhang L., Huang Y. (2018). Iridium-catalyzed aerobic *α*,*β*-dehydrogenation of *γ*,*δ*-unsaturated amides and acids: activation of both *α*- and *β*-C−H bonds through an Allyl−Iridium intermediate. J. Am. Chem. Soc..

[bib68] Willis M.C. (2010). Transition metal catalyzed alkene and alkyne hydroacylation. Chem. Rev..

[bib69] Woerly E.M., Roy J., Burke M.D. (2014). Synthesis of most polyene natural product Motifs using just 12 building blocks and one coupling reaction. Nat. Chem..

[bib70] Xiong X.-F., Zhou Q., Gu J., Dong L., Liu T.-Y., Chen Y.-C. (2012). Trienamine catalysis with 2,4-dienones: development and application in asymmetric diels–alder reactions. Angew. Chem. Int. Ed..

[bib71] Yoshii D., Jin X., Yatabe T., Hasegawa J.-Y., Yamaguchi K., Mizuno N. (2016). Gold nanoparticles on OMS-2 for heterogeneously catalyzed aerobic oxidative *α*,*β*-dehydrogenation of *β*-Heteroatom-substituted ketones. Chem. Commun. (Camb.).

[bib72] Yuan F.-Q., Han F.-S. (2012). Synthesis of densely substituted *α*,*β*,*γ*,*δ*-dienones via the Pd^II^-catalyzed allylation, H-migration, and aerobic oxidative *δ*-hydride elimination cascade. Org. Lett..

[bib73] Zhang P., Morken J.P. (2009). Catalytic enantioselective allylation of dienals through the intermediacy of unsaturated *π*-allyl complexes. J. Am. Chem. Soc..

