# Supplementary material for: Synthesis of (E,E)-Dienones and (E,E)-Dienals via Palladium-Catalyzed γ,δ-Dehydrogenation of Enones and Enals
Source: iScience. 2019 Sep 21;20:229–36. doi: 10.1016/j.isci.2019.09.027 (PMC6817633; doi:10.1016/j.isci.2019.09.027)
Supplement: Document S1. Transparent Methods and Figures S1–S110 [file mmc1.pdf]

ISCI, Volume *20*

Supplemental Information

Synthesis of (*E*,*E*)-Dienones and (*E*,*E*)-Dienals via Palladium-Catalyzed γ,δ-Dehydrogenation of Enones and Enals

Gao-Fei Pan, Xing-Long Zhang, Xue-Qing Zhu, Rui-Li Guo, and Yong-Qiang Wang

Transparent Methods

1. General information

All commercial reagents and solvents were used as received without further purification. Reactions were followed with TLC (0.254 mm silica gel 60-F plates). Visualization was accomplished with UV light. Flash chromatographies were carried out on silica gel 200-300 mesh. Optical rotations were reported as follows: $[\alpha]^{27}_{D}$ (*c* g/100 mL, in solvent). Melting points (m. p.) were measured on electrothermal digital melting point apparatus and were uncorrected. ^{1}HNMR and ^{13}CNMR spectra were recorded at 400 MHz using $CDCl_3$ as solvent. Spectra were referenced internally to the residual proton resonance in $CDCl_3$ (δ 7.26 ppm) with tetramethylsilane (TMS, δ 0.00 ppm) as the internal standard. Chemical shifts (δ) were reported as part per million (ppm) in δ scale downfield from TMS. Multiplicities are reported as follows: s = singlet, d = doublet, t = triplet, m = multiplet, br. s = broad singlet. Infrared (IR) data were recorded as films on potassium bromide plates on a Bruker Tensor 27 FT-IR spectrometer. Absorbance frequencies are reported in reciprocal centimeters (cm^{-1}). High resolution mass spectra were acquired on a Bruker Daltonics MicroTof-Q II mass spectrometer. X-ray crystal structure analyses were measured on Bruker Smart APEXIICCD instrument using Mo-Kα radiation. The structures were solved and refined using the SHELXTL software package.

2. General Procedure for the Palladium-Catalyzed *γ*,*δ*-Dehydrogenation reaction

A seal tube containing enones or enals (0.5 mmol) and $Pd(OAc)_2$ (10 mol%), was evacuated and filled with dioxygen gas using an oxygen containing balloon. Then, DMSO (2.5 mL), trifluoroacetic acid (TFA) (1.0 mmol) were sequentially added to the system via syringe under an oxygen atmosphere. The reaction mixture was stirred at 80 ℃ until completion of the reaction (TLC). Then the reaction was cooled to room temperature and partitioned between water and ethyl acetate. The layers were separated and the organic layer was washed with water (3 × 5.0 mL) and washed with aqueous saturated brine solution (3 × 5.0 mL), dried over Na_2SO_4, filtered and concentrated under reduced pressure. Purification by flash chromatography afforded the (*E*, *E*)-dienones or (*E*, *E*)-dienals.

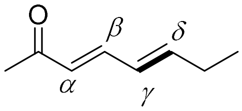

2aa

(*3E*, *5E*)-octa-3,5-dien-2-one (2aa) (Ma et al., 1989) Prepared according to general procedure to afford as pale yellow oil (73% yield). R_f= 0.56 (EtOAc / hexanes 1 : 30). ^{1}H NMR (400 MHz, $CDCl_3$): δ = 7.11 (dd, J = 15.6, 9.6 Hz, 1H, β-H), 6.31 – 6.13 (m, 2H, γ-H and δ-H), 6.06 (d, J = 15.7 Hz, 1H, α-H), 2.26 (s, 3H, CH_3), 2.22 – 2.10 (m, 2H, CH_2), 1.06 (t, J = 7.4 Hz, 3H, CH_3). ^{13}C NMR (100 MHz, $CDCl_3$): δ = 198.6, 147.0, 144.0, 128.8, 127.9, 27.1, 26.1, 12.8. HRMS (ESI) for $C_8H_{13}O$ [M+H$^+$]: Calcd: 147.0780; Found: 147.0788. IR (KBr): 2925, 2854, 1722, 1670, 1459, 1257, 1054, 1012, 800, 617 cm^{-1}.

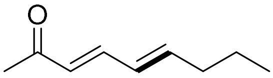

2ab

(*3E*, *5E*)-nona-3,5-dien-2-one (2ab) (Ma et al., 1989) Prepared according to general procedure to afford as pale yellow oil (72% yield). R_f= 0.50 (EtOAc / hexanes 1 : 30). ^{1}H NMR (400 MHz, $CDCl_3$): δ = 7.19 – 7.02 (m, 1H, β-H), 6.21 – 6.14 (m, 2H, γ-H and δ-H), 6.06 (d, J = 15.6 Hz, 1H, α-H), 2.26 (s, 3H, CH_3), 2.21 – 2.13 (m, 2H, CH_2), 1.53 – 1.42 (m, 2H, CH_2), 0.93 (t, J = 7.4 Hz, 3H, CH_3). ^{13}C NMR (100 MHz , $CDCl_3$): δ = 198.9, 145.6, 144.1, 129.0, 128.8, 35.2, 27.2, 22.0, 13.7. HRMS (ESI) for $C_9H_{14}NaO$ [M+Na$^+$]: Calcd: 161.0937; Found: 161.0943. IR (KBr): 3448, 2965,1724, 1677, 1459, 1361, 1284, 1130, 981, 742 cm^{-1}.

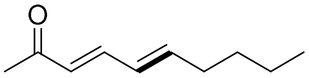

2ac

(*3E*, *5E*)-deca-3,5-dien-2-one (2ac) (Yoo et al., 2006) Prepared according to general procedure to afford as colorless oil (71% yield). R_f= 0.45 (EtOAc / hexanes 1 : 30). ^{1}H NMR (400 MHz, $CDCl_3$): δ = 7.14 – 7.05 (m, 1H, β-H), 6.22 – 6.17 (m, 2H, γ-H and δ-H), 6.06 (d, J = 15.6 Hz, 1H, α-H), 2.19 (s, 3H, CH_3), 2.23 – 2.16 (m, 2H, CH_2), 1.47 – 1.37 (m, 2H, CH_2), 1.37 – 1.28 (m, 2H, CH_2), 0.89 (t, J = 7.2 Hz, 3H, CH_3). ^{13}C NMR (100 MHz , $CDCl_3$): δ = 198.9, 146.0, 144.2, 128.9, 128.8, 32.9, 30.9, 27.2,

22.4, 14.0. HRMS (ESI) for $C_{10}H_{16}NaO$ [M+Na$^+$]: Calcd: 175.1093; Found: 175.1086. IR (KBr): 2940, 2834, 1704, 1457, 1376, 1267, 1193, 1126, 1027 cm^{-1}.

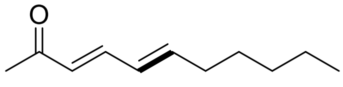

2ad

(*3E*, *5E*)-undeca-3,5-dien-2-one (2ad) (Ma et al., 1989) Prepared according to general procedure to afford as colorless oil (70% yield). R_f= 0.45 (EtOAc / hexanes 1 : 30). ^{1}H NMR (400 MHz, $CDCl_3$): δ = 7.15 – 7.06 (m, 1H, β-H), 6.21 – 6.16 (m, 2H, γ-H and δ-H), 6.05 (d, J = 15.5 Hz, 1H, α-H), 2.26 (s, 3H, CH_3), 2.22 – 2.15 (m, 2H, CH_2), 1.48 – 1.40 (m, 2H, CH_2), 1.38 – 1.23 (m, 4H, $2CH_2$), 0.90 (t, J = 6.9 Hz, 3H, CH_3). ^{13}C NMR (100 MHz, $CDCl_3$): δ = 198.8, 145.9, 144.1, 128.8, 128.8, 33.1, 31.4, 28.4, 27.1, 22.5, 14.0. HRMS (ESI) for $C_{11}H_{18}NaO$ [M+Na$^+$]: Calcd: 189.1250; Found: 189.1238. IR (KBr): 2927, 2859, 1668, 1459, 1359, 1253, 1151, 998 cm^{-1}.

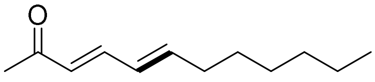

2ae

(*3E*, *5E*)-dodeca-3,5-dien-2-one (2ae) Prepared according to general procedure to afford as pale yellow (79% yield). R_f= 0.36 (EtOAc / hexanes 1 : 30). ^{1}H NMR (400 MHz, $CDCl_3$): δ = 7.14 – 7.06 (m, 1H, β-H), 6.21 – 6.16 (m, 2H, γ-H and δ-H), 6.05 (d, J = 15.6 Hz, 1H, α-H), 2.26 (s, 3H, CH_3), 2.22 – 2.16 (m, 2H, CH_2), 1.46 – 1.39 (m, 2H, CH_2), 1.36 – 1.27 (m, 6H, $3CH_2$), 0.89 (t, J = 6.7 Hz, 3H, CH_3). ^{13}C NMR (100 MHz, $CDCl_3$): δ = 198.8, 145.9, 144.1, 128.9, 128.8, 33.2, 31.7, 28.9, 28.7, 27.2, 22.6, 14.1. HRMS (ESI) for $C_{12}H_{21}O$ [M+H$^+$]: Calcd: 181.1587; Found: 181.1590. IR (KBr): 2958, 2922, 2854, 1658, 1584, 1450, 1263, 1096, 1025, 803 cm^{-1}.

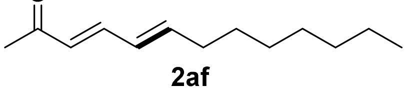

2af

(*3E*, *5E*)-trideca-3,5-dien-2-one (2af) Prepared according to general procedure to afford as pale yellow oil (73% yield). R_f = 0.45 (EtOAc / hexanes 1 : 30). ^{1}H NMR (400 MHz, $CDCl_3$): δ = 7.14 – 7.06 (m, 1H, β-H), 6.20 – 6.15 (m, 2H, γ-H and δ-H), 6.05 (d, J = 15.5 Hz, 1H, α-H), 2.26 (s, 3H, CH_3), 2.21 – 2.15 (m, 2H, CH_2), 1.47 –

1.41 (m, 2H, CH_2), 1.33 – 1.20 (m, 8H, $4CH_2$), 0.88 (t, J = 6.9 Hz, 3H, CH_3). ^{13}C NMR (100 MHz, $CDCl_3$): δ = 198.9, 146.0, 144.2, 128.9, 128.8, 33.2, 31.9, 29.2, 29.2, 28.8, 27.2, 22.7, 14.2. HRMS (ESI) for $C_{13}H_{22}NaO$ [M+Na$^+$]: Calcd: 217.1563; Found: 217.1572. IR (KBr): 2925, 2854, 1675, 1458, 1257, 1187, 1124, 1027, 725 cm^{-1}.

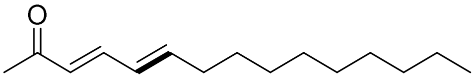

2ag

(*3E*, *5E*)-pentadeca-3,5-dien-2-one (2ag) Prepared according to general procedure to afford as pale yellow oil (72% yield). R_f= 0.56 (EtOAc / hexanes 1 : 30); ^{1}H NMR (400 MHz, $CDCl_3$): δ = 7.14 – 7.06 (m, 1H, β-H), 6.21 – 6.15 (m, 2H, γ-H and δ-H), 6.05 (d, J = 15.5 Hz, 1H, α-H), 2.26 (s, 3H, CH_3), 2.21 – 2.14 (m, 2H, CH_2), 1.46 – 1.38 (m, 2H, CH_2), 1.32 – 1.24 (m, 12H, $6CH_2$), 0.88 (t, J = 6.9 Hz, 3H, CH_3). ^{13}C NMR (100 MHz, $CDCl_3$): δ = 198.9, 146.0, 144.2, 128.9, 128.9, 33.3, 32.0, 29.6, 29.5, 29.4, 29.3, 28.8, 27.2, 22.8, 14.2. HRMS (ESI) for $C_{15}H_{26}NaO$ [M+Na$^+$]: Calcd: 245.1876; Found: 245.1882. IR (KBr): 3411, 2927, 2856, 1671, 1459, 1363, 1257, 1189, 1156, 1027 cm^{-1}.

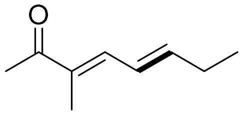

2ah

(*3E*, *5E*)-3-methylnona-3,5-dien-2-one (2ah) Prepared according to general procedure to afford as pale yellow oil (76% yield). R_f= 0.43 (EtOAc / hexanes 1 : 20). ^{1}H NMR (400 MHz, $CDCl_3$): δ = 7.02 (d, J = 10.9 Hz, 1H, β-H), 6.50 – 6.38 (m, 1H, γ-H), 6.22 – 6.14 (m, 1H, δ-H), 2.33 (s, 3H, CH_3), 2.30 – 2.20 (m, 2H, CH_2), 1.87 (s, 3H, CH_3), 1.08 (t, J = 7.5 Hz, 3H, CH_3). ^{13}C NMR (100 MHz, $CDCl_3$): δ = 200.0, 145.5, 140.0, 134.8, 125.8, 26.6, 25.6, 13.3, 11.5. HRMS (ESI) for $C_{10}H_{16}NaO$ [M+Na$^+$]: Calcd: 175.1093; Found: 175.1086. IR (KBr): 2958, 2930, 2872, 1715, 1674, 1459, 1372, 1239, 1169, 976 cm^{-1}.

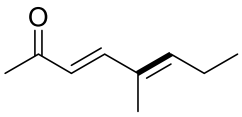

2ai

(*3E*, *5E*)-5-methylocta-3,5-dien-2-one (2ai) (Zou et al., 2008) Prepared according to general procedure to afford as pale yellow oil (72% yield). R_f= 0.53 (EtOAc / hexanes 1 : 20). ^{1}H NMR (400 MHz, $CDCl_3$): δ = 7.15 (d, J = 16.0 Hz, 1H, β-H), 6.08 (d, J = 16.0 Hz, 1H, α-H), 5.94 (t, J = 7.3 Hz, 1H, δ-H), 2.29 (s, 3H, CH_3), 2.28 – 2.18 (m, 2H, CH_2), 1.78 (s, 3H, CH_3), 1.04 (t, J = 7.5 Hz, 3H, CH_3). ^{13}C NMR (100 MHz, $CDCl_3$): δ = 199.1, 148.9, 145.0, 132.7, 125.3, 27.3, 22.4, 13.6, 12.1. HRMS (ESI) for $C_9H_{14}NaO$ [M+Na$^+$]: Calcd: 161.0937; Found: 161.0950. IR (KBr): 2964, 1671, 1574, 1455, 1359, 1257, 1176, 1045, 977 cm^{-1}.

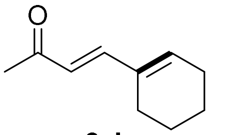

2aj

(*E*)-4-(cyclohex-1-en-1-yl)but-3-en-2-one (2aj) (Polaquini et al., 2017) Prepared according to general procedure to afford as yellow oil (75% yield). R_f = 0.43 (EtOAc / hexanes 1 : 20); ^{1}H NMR (400 MHz, $CDCl_3$): δ = 7.12 (d, J = 16.1 Hz, 1H, β-H), 6.22 (t, J = 3.9 Hz, 1H, δ-H), 6.05 (d, J = 16.1 Hz, 1H, α-H), 2.28 (s, 3H, CH_3), 2.26 – 2.18 (m, 2H, CH_2), 2.17 – 2.12 (m, 2H, CH_2), 1.75 – 1.67 (m, 2H, CH_2), 1.67 – 1.60 (m, 2H, CH_2). ^{13}C NMR (100 MHz, $CDCl_3$): δ = 199.1, 147.2, 140.0, 135.3, 124.2, 27.2, 26.7, 24.2, 22.1, 22.0. HRMS (ESI) for $C_{10}H_{14}NaO$ [M+Na$^+$]: Calcd: 173.0937; Found: 173.0932. IR (KBr): 2935, 2863, 1671, 1621, 1428,1359, 1257, 1172, 1074 cm^{-1}.

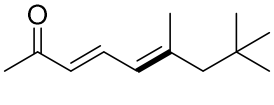

2ak

(*3E*, *5E*)-6,8,8-trimethylnona-3,5-dien-2-one (2ak) Prepared according to general procedure to afford as pale yellow oil (78% yield). R_f = 0.42 (EtOAc / hexanes 1 : 20); ^{1}H NMR (400 MHz, $CDCl_3$): 7.44 (dd, J = 15.3, 11.5 Hz, 1H, β-H), 6.09 (d, J = 15.3 Hz, 1H , α-H), 5.96 (d, J = 11.4 Hz, 1H, γ-H), 2.28 (s, 3H, CH_3), 2.07 (s, 2H, CH_2), 1.97 (s, 3H, CH_3), 0.94 (s, 9H, 3CH_3). ^{13}C NMR (100 MHz, $CDCl_3$): δ = 199.0, 149.8,

139.5, 128.5, 127.1, 54.4, 32.7, 30.2 (3 C), 27.7, 20.4. HRMS (ESI) for $C_{12}H_{21}O$ [M+H$^+$]: Calcd: 181.1587; Found: 181.1585. IR (KBr): 2957, 2924, 2855, 1654, 1583, 1434, 1223, 1089, 1011, 812 cm^{-1}.

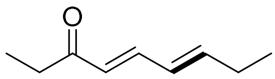

2al

(*4E*, *6E*)-nona-4,6-dien-3-one (2al) (Ma et al., 1989) Prepared according to general procedure to afford as colorless oil (78% yield). R_f= 0.40 (EtOAc / hexanes 1 : 30). ^{1}H NMR (400 MHz, $CDCl_3$): δ = 7.17 (dd, J = 15.5, 9.6 Hz, 1H, β-H), 6.30 – 6.15 (m, 2H , γ-H and δ-H), 6.10 (d, J = 15.6 Hz, 1H, α-H), 2.58 (q, J = 7.3 Hz, 2H, CH_3), 2.28 – 2.14 (m, 2H, CH_2), 1.11 (t, J = 7.4 Hz, 3H, CH_3), 1.06 (t, J = 7.4 Hz, 3H, CH_3). ^{13}C NMR (100 MHz, $CDCl_3$): δ = 201.6, 146.9, 143.0, 128.1, 127.8, 33.8, 26.3, 13.0, 8.5. HRMS (ESI) for $C_9H_{14}NaO$ [M+Na$^+$]: Calcd: 161.0937; Found: 161.0950. IR (KBr): 2960, 2924, 1957, 1261, 1091, 1023, 800 cm^{-1}.

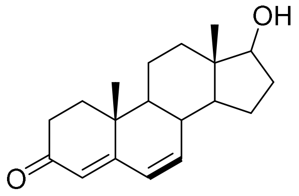

2am

(*10R*,*3S*)-17-hydroxy-10,13-dimethyl-1,2,8,9,10,11,12,13,14,15,16,17-dodecahydro -3H-cyclopenta[a]phenanthren-3-one (2am) (Peart et al., 2011) Prepared according to general procedure to afford as white solid (62% yield). R_f = 0.38 (EtOAc / hexanes 1 : 20). m. p. = 224 – 226 °C. ^{1}H NMR (400 MHz, $CDCl_3$): δ = 6.11 (s, 2H, γ-H and δ-H), 5.68 (s, 1H, α-H), 3.70 (t, J = 8.3 Hz, 1H, CH), 2.65 – 2.50 (m, 1H), 2.47 – 2.38 (m, 1H), 2.25 (t, J = 10.7 Hz, 1H, CH), 2.20 – 2.08 (m, 1H), 2.05 – 1.97 (m, 1H), 1.97 – 1.86 (m, 1H), 1.85 – 1.76 (m, 2H), 1.75 – 1.66 (m, 1H), 1.65 – 1.57 (m, 1H), 1.55 – 1.42 (m, 3H), 1.23 (br, 1H), 1.22 – 1.15 (m, 2H), 1.13 (s, 3H, CH_3), 0.85 (s, 3H, CH_3). ^{13}C NMR (100 MHz, $CDCl_3$) : δ = 199.8, 163.9, 140.6, 128.1, 123.8, 81.3, 50.9, 48.4, 44.0, 37.8, 36.4, 36.2, 34.03, 34.02, 30.5, 23.1, 20.4, 16.4, 11.1. HRMS (ESI) for $C_{19}H_{11}NaO_2$ [M+Na$^+$]: Calcd: 309.1825; Found: 309.1824. IR (KBr): 3335, 3025, 2964, 2928, 2859, 1657, 1614, 1583, 1061 cm^{-1}.

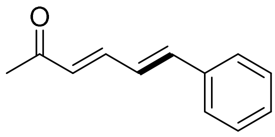

2an

(*3E*, *5E*)-6-phenylhexa-3,5-dien-2-one (2an) (Wu et al., 2016) Prepared according to general procedure to afford as yellow solid (89% yield). R_f = 0.46 (EtOAc / hexanes 1 : 20); m. p. = 37 – 38 °C. ^{1}H NMR (400 MHz, $CDCl_3$): δ = 7.45 (d, J = 7.4 Hz, 2H, Ar-H), 7.37 – 7.22 (m, 4H, Ar-H and β-H), 6.95 – 6.80 (m, 2H, γ-H and δ-H), 6.23 (d, J = 15.5 Hz, 1H, α-H), 2.29 (s, 3H, CH_3). ^{13}C NMR (100 MHz, $CDCl_3$): δ = 198.4, 143.5, 141.3, 136.0, 130.5, 129.2, 128.9, 127.3, 126.7, 27.4. HRMS (ESI) for $C_{12}H_{13}O$ [M+H$^+$]: Calcd: 173.0961; Found: 173.0950. IR (KBr): 3059, 3028, 3000, 1960, 1882, 1712, 1653, 1614, 1591, 1360, 1254, 995 cm^{-1}.

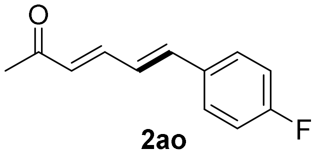

2ao

(*3E*, *5E*)-6-(4-fluorophenyl)hexa-3,5-dien-2-one (2ao) (Wu et al., 2016) Prepared according to general procedure to afford as yellow solid (86% yield). R_f = 0.55 (EtOAc / hexanes 1 : 20); m. p. = 40 – 41 °C. ^{1}H NMR (400 MHz, $CDCl_3$): δ = 7.45 (dd, J = 8.6, 5.5 Hz, 2H, Ar-H), 7.27 (dd, J = 15.5, 10.6 Hz, 1H, β-H), 7.07 – 7.03 (m, 2H, Ar-H), 6.94 – 6.75 (m, 2H, γ-H and δ-H), 6.25 (d, J = 15.5 Hz, 1H, α-H), 2.31 (s, 3H, CH_3). ^{13}C NMR (100 MHz, $CDCl_3$): δ = 198.6, 163.30 (d, J = 250.1 Hz), 143.4, 140.0, 132.3 (d, J = 3.4 Hz), 130.6, 129.1 (d, J = 8.3 Hz), 126.5 (d, J = 2.5 Hz), 116.1 (d, J = 21.9 Hz). 27.5. HRMS (ESI) for $C_{12}H_{12}FO$ [M+H$^+$]: Calcd: 191.0867; Found: 191.0877. IR (KBr): 2996, 2928, 2922, 1713, 1682, 1360, 1229, 1178, 978, 811 cm^{-1}.

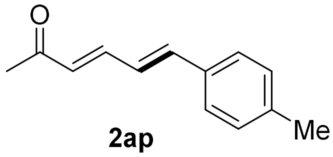

2ap

(*3E*, *5E*)-6-(p-tolyl)hexa-3,5-dien-2-one (2ap) (Wu et al., 2016) Prepared according to general procedure to afford as pale yellow solid (90% yield). R_f = 0.46 (EtOAc / hexanes 1 : 20); m. p. = 60 – 61 °C. ^{1}H NMR (400 MHz, $CDCl_3$): δ = 7.35 (d, J = 8.1 Hz, 2H, Ar-H), 7.27 (dd, J = 15.5, 10.3 Hz, 1H, β-H), 7.15 (d, J = 8.0 Hz, 2H, Ar-H),

6.93 – 6.77 (m, 2H, *γ*-H and *δ*-H), 6.21 (d, *J* = 15.5 Hz, 1H, *α*-H), 2.35 (s, 3H, CH_3), 2.29 (s, 3H, CH_3). ^{13}C NMR (100 MHz, $CDCl_3$): *δ* = 198.4, 143.8, 141.4, 139.5, 133.3, 130.0, 129.6, 127.3, 125.7, 27.3, 21.4. HRMS (ESI) for $C_{13}H_{15}O$ [M+H$^+$]: Calcd: 187.1117; Found: 187.1115. IR (KBr): 3073, 2996, 2928, 1713, 1599, 1590, 1509, 1417, 1360, 1228, 1157, 977, 830 cm^{-1}.

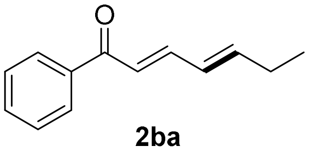

2ba

(*2E*, *4E*)-1-phenylhepta-2,4-dien-1-one (2ba) (Ma et al.,1989) Prepared according to general procedure to afford as yellow oil (65% yield). R_f= 0.39 (EtOAc / hexanes 1 : 30); ^1H NMR (400 MHz, $CDCl_3$): *δ* = 7.99 – 7.90 (m, 2H, Ar-H), 7.58 – 7.52 (m, 1H, Ar-H), 7.51 – 7.45 (m, 2H, Ar-H), 7.43 – 7.35 (m, 1H, *β*-H), 6.89 (d, *J* = 15.0 Hz, 1H, *α*-H), 6.33 – 6.25 (m, 2H, *γ*-H and *δ*-H), 2.29 – 2.20 (m, 2H, CH_2), 1.08 (t, *J* = 7.4 Hz, 3H, CH_3). ^{13}C NMR (100 MHz, $CDCl_3$): *δ* = 191.1, 148.0, 145.7, 138.4, 132.7, 128.7, 128.5, 128.3, 123.7, 26.4, 13.0. HRMS (ESI) for $C_{13}H_{15}O$ [M+H$^+$]: Calcd: 187.1117; Found: 187.1115. IR (KBr): 3062, 2960, 2867, 1673, 1619, 1452, 1284, 1103, 1010, 798, 696 cm^{-1}.

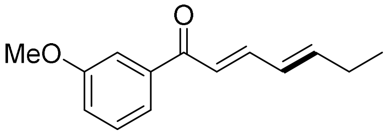

2bb

(*2E*, *4E*)-1-(3-methoxyphenyl)hepta-2,4-dien-1-one (2bb) Prepared according to general procedure to afford as yellow oil (71% yield). R_f= 0.47 (EtOAc / hexanes 1 : 10). ^1H NMR (400 MHz, $CDCl_3$): *δ* = 7.56 – 7.47 (m, 2H, Ar-H), 7.47 – 7.33 (m, 2H, Ar-H and *β*-H), 7.10 (dd, *J* = 8.0, 2.4 Hz, 1H, Ar-H), 6.87 (d, *J* = 15.0 Hz, 1H, *α*-H), 6.34 – 6.29 (m, 2H, *γ*-H and *δ*-H), 3.87 (s, 3H, OCH_3), 2.29 – 2.19 (m, 2H , CH_2), 1.08 (t, *J* = 7.4 Hz, 3H , CH_3). ^{13}C NMR (100 MHz, $CDCl_3$) *δ* = 190.7, 160.0, 148.1, 145.7, 139.8, 129.6, 128.3, 123.7, 121.0, 119.3, 112.8, 55.6, 26.4, 13.0. HRMS (ESI) for $C_{14}H_{17}O_2$ [M+H$^+$]: Calcd: 217.1223; Found: 217.1228. IR (KBr): 3075, 2959, 2931, 2035, 1720, 1680, 1590, 1487, 1459, 1430, 1265, 1041, 756 cm^{-1}.

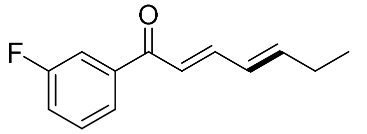

2bc

(*2E*, *4E*)-1-(3-fluorophenyl)hepta-2,4-dien-1-one (2bc) Prepared according to general procedure to afford as yellow oil (65% yield). R_f= 0.54 (EtOAc / hexanes 1 : 10). ^{1}H NMR (400 MHz, $CDCl_3$): δ = 7.71 (d, J = 7.8 Hz, 1H, Ar-H), 7.67 – 7.60 (m, 1H, Ar-H), 7.48 – 7.39 (m, 2H, Ar-H and β-H), 7.28 – 7.21 (m, 1H, Ar-H), 6.84 (d, J = 14.9 Hz, 1H, α-H), 6.36 – 6.30 (m, 2H, γ-H and δ-H), 2.30 – 2.19 (m, 2H, CH_2), 1.08 (t, J = 7.5 Hz, 3H, CH_3). ^{13}C NMR (100 MHz, $CDCl_3$): δ = 189.6, 163.0 (d, J = 247.7 Hz), 148.7, 146.4, 140.6 (d, J = 6.2 Hz), 130.3 (d, J = 7.7 Hz), 128.2, 124.1 (d, J = 2.9 Hz), 123.2, 119.6 (d, J = 21.5 Hz), 115.3 (d, J = 22.3 Hz). 26.4, 12.9. HRMS (ESI) for $C_{13}H_{14}O$ [M+Na$^+$]: Calcd: 227.0831; Found: 227.0833. IR (KBr): 2959, 2927, 2867, 1724, 1599, 1506, 1461, 1410, 1273, 1235, 1156, 1038, 844 cm^{-1}.

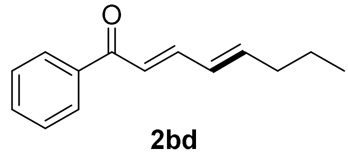

2bd

(*2E*, *4E*)-1-phenylocta-2,4-dien-1-one (2bd) (Kim et al., 2015) Prepared according to general procedure to afford as yellow oil (65% yield). R_f= 0.55 (EtOAc / hexanes 1 : 10). ^{1}H NMR (400 MHz, $CDCl_3$): δ = 7.96 – 7.91 (m, 2H, Ar-H), 7.58 – 7.51 (m, 1H, Ar-H), 7.50 – 7.36 (m, 2H, Ar-H), 7.41 (dd, J = 15.1, 10.4 Hz, 1H, β-H), 6.88 (d, J = 15.1, 1H, α-H), 6.38 – 6.20 (m, 2H, γ-H and δ-H), 2.25 – 2.17 (m, 2H, CH_2), 1.54 – 1.43 (m, 2H, CH_2), 0.94 (t, J = 7.4 Hz, 3H, CH_3). ^{13}C NMR (100 MHz, $CDCl_3$): δ = 191.1, 146.5, 145.6, 138.4, 132.6, 129.4, 128.5, 123.7, 35.4, 22.1, 13.8. HRMS (ESI) for $C_{14}H_{16}NaO$ [M+Na$^+$]: Calcd: 223.1093; Found: 223.1085. IR (KBr): 3015, 2961, 2860, 1660, 1591, 1452, 1351, 1268, 1090, 692 cm^{-1}.

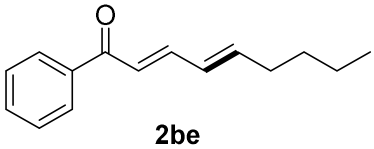

2be

(*2E*, *4E*)-1-phenylnona-2,4-dien-1-one (2be) (Armstrong et al., 2010) Prepared according to general procedure to afford as yellow oil (71% yield). R_f= 0.28 (EtOAc /

hexanes 1 : 20). ^{1}H NMR (400 MHz, $CDCl_3$): δ = 7.96 – 7.91 (m, 2H, Ar-H), 7.58 – 7.51 (m, 1H, Ar-H), 7.50 – 7.43 (m, 2H, Ar-H), 7.41 (dd, J = 15.0, 10.0 Hz, 1H, β-H), 6.88 (d, J = 15.1 Hz, 1H, α-H), 6.37 – 6.20 (m, 2H, γ-H and δ-H), 2.27 – 2.15 (m, 2H, CH_2), 1.52 – 1.23 (m, 4H, $2CH_2$), 0.92 (t, J = 7.2 Hz, 3H, CH_3). ^{13}C NMR (100 MHz, $CDCl_3$): δ = 191.1, 146.8, 145.6, 138.5, 132.6, 129.3, 128.7, 128.6, 128.5, 123.7, 33.0, 31.0, 22.4, 14.0. HRMS (ESI) for $C_{15}H_{18}NaO$ [M+Na^{+}]: Calcd: 237.1250; Found: 237.1245. IR (KBr): 3025, 2960, 2867, 1662, 1592, 1450, 1353, 1268, 1090, 696 cm^{-1}.

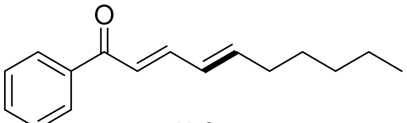

2bf

(*2E*, *4E*)-1-phenyldeca-2,4-dien-1-one (2bf) Prepared according to general procedure to afford as yellow oil (65% yield). R_f= 0.50 (EtOAc / hexanes 1 : 20). ^{1}H NMR (400 MHz, $CDCl_3$): δ= 7.97 – 7.90 (m, 2H, Ar-H), 7.57 – 7.52 (m, 1H, Ar-H), 7.50 – 7.44 (m, 2H, Ar-H), 7.40 (dd, J = 15.1, 10.0 Hz, 1H , β-H), 6.88 (d, J = 15.1 Hz, 1H, α-H), 6.39 – 6.20 (m, 2H, γ-H and δ-H), 2.27 – 2.15 (m, 2H, CH_2), 1.51 – 1.40 (m, 2H, CH_2), 1.38 – 1.24 (m, 4H, CH_2), 0.90 (t, J = 6.9 Hz, 3H, CH_3). ^{13}C NMR (100 MHz, $CDCl_3$) : δ = 191.1, 146.8, 145.6, 138.5, 132.6, 129.2, 128.7, 128.5, 123.7, 33.3, 31.5, 28.5, 22.6, 14.1. HRMS (ESI) for $C_{16}H_{21}O$ [M+H^{+}]: Calcd: 229.1587; Found: 229.1590. IR (KBr): 2917, 2863, 1664, 1592, 1452, 1263, 1008, 769, 696 cm^{-1}.

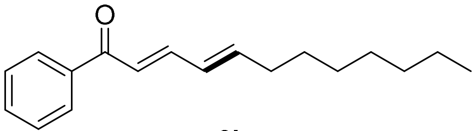

2bg

(*2E*, *4E*)-1-phenyldodeca-2,4-dien-1-one (2bg) Prepared according to general procedure to afford as yellow oil (65% yield). R_f= 0.56 (EtOAc / hexanes 1 : 20); ^{1}H NMR (400 MHz, $CDCl_3$): δ = 7.98 – 7.90 (m, 2H, Ar-H), 7.59 – 7.51 (m, 1H, Ar-H), 7.50 – 7.43 (m, 2H, Ar-H), 7.41 (dd, J = 15.1, 10.0 Hz, 1H, β-H), 6.89 (d, J = 15.1 Hz, 1H, α-H), 6.39 – 6.19 (m, 2H, γ-H and δ-H), 2.28 – 2.15 (m, 2H, CH_2), 1.52 – 1.39 (m, 2H, CH_2), 1.33 – 1.27 (m, 8H, $4CH_2$), 0.89 (t, J = 6.8 Hz, 3H, CH_3). ^{13}C NMR (100 MHz, $CDCl_3$): δ = 191.1, 146.9, 145.7, 138.4, 132.6, 129.2, 128.6, 128.5, 123.6, 33.4,

31.9, 29.3, 29.2, 28.8, 22.8, 14.2. HRMS (ESI) for $C_{18}H_5O$ [M+H$^+$]: Calcd: 257.1900; Found: 257.1891. IR (KBr): 2919, 2950, 1658, 1585, 1463, 1357, 1268, 1074, 1001, 692 cm^{-1}.

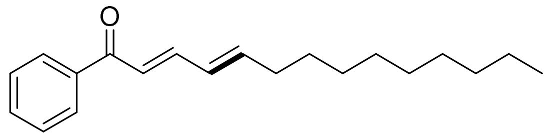

2bh

(*2E*, *4E*)-1-phenyltetradeca-2,4-dien-1-one (2bh) (Kim et al., 2015) Prepared according to general procedure to afford as yellow oil (56% yield). R_f= 0.43 (EtOAc / hexanes 1 : 20); ^{1}H NMR (400 MHz, CDCl$_3$): δ = 7.96 – 7.91 (m, 2H, Ar-H), 7.59 – 7.52 (m, 1H, Ar-H), 7.51 – 7.44 (m, 2H, Ar-H), 7.43 (dd, J = 15.1, 10.0 Hz, 1H, β-H), 6.88 (d, J = 15.1 Hz, 1H, α-H), 6.38 – 6.20 (m, 2H, γ-H and δ-H), 2.28 – 2.15 (m, 2H, CH$_2$), 1.50 – 1.40 (m, 2H, CH$_2$), 1.35 – 1.21 (m, 12H, 6CH$_2$), 0.88 (t, J = 6.8 Hz, 3H, CH$_3$). ^{13}C NMR (100 MHz, CDCl$_3$): δ = 191.1, 146.9, 145.7, 138.5, 132.6, 129.2, 128.7, 128.5, 123.7, 33.4, 32.0, 29.7, 29.6, 29.5, 29.4, 28.8, 22.8, 14.3. HRMS (ESI) for $C_{20}H_{29}O$ [M+H$^+$]: Calcd: 285.2213; Found: 285.2217. IR (KBr): 2950, 2919, 1658, 1585, 1463, 1357, 1268, 1074, 1001, 692 cm^{-1}.

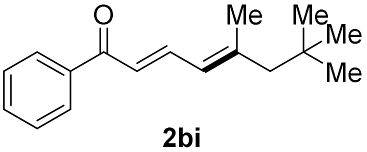

2bi

(*2E*, *4E*)-5,7,7-trimethyl-1-phenylocta-2,4-dien-1-one (2bi) Prepared according to general procedure to afford as yellow oil (66% yield). R_f= 0.43 (EtOAc / hexanes 1 : 20). ^{1}H NMR (400 MHz, CDCl$_3$): δ = 7.98 – 7.95 (m, 2H, Ar-H), 7.77 (dd, J = 14.8, 11.8 Hz, 1H, β-H), 7.74 – 7.51 (m, 1H, Ar-H), 7.49-7.45 (m, 2H, Ar-H), 6.92 (d, J = 14.8 Hz, 1H, α-H), 6.11 (d, J = 11.8 Hz, 1H, γ-H), 2.10 (s, 2H, CH$_2$), 2.01 (s, 3H, CH$_3$), 0.95 (s, 9H, 3CH$_3$). ^{13}C NMR (100 MHz, CDCl$_3$): δ = 191.1, 150.9, 141.2, 138.6, 132.6, 128.6, 128.4, 127.6, 123.3, 54.4, 32.8, 30.3, 20.6. HRMS (ESI) for $C_{17}H_{22}NaO$ [M+H$^+$]: Calcd: 265.1563; Found: 265.1579. IR (KBr): 3062, 3030, 2954, 2926, 2865, 1968, 1598, 1490, 1450, 1269, 1180, 1007, 694 cm^{-1}.

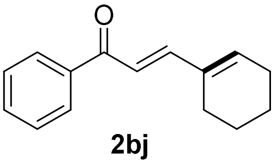

2bj

(*E*)-3-(cyclohex-1-en-1-yl)-1-phenylprop-2-en-1-one (2bj) (Kim et al., 2015) Prepared according to general procedure to afford as yellow oil (76% yield). R_f = 0.43 (EtOAc / hexanes 1 : 30). ^{1}H NMR (400 MHz, $CDCl_3$): δ = 7.96 – 7.91 (m, 2H, Ar-H), 7.59 – 7.51 (m, 1H, Ar-H), 7.49 – 7.46 (m, 2H, Ar-H), 7.42 (d, J = 16.0 Hz, 1H, β-H), 6.85 (d, J = 15.4 Hz, 1H, α-H) , 6.29 (t, J = 12.0 Hz, 1H, δ-H), 2.34 – 2.19 (m, 4H, CH_2), 1.78 – 1.69 (m, 2H, CH_2), 1.69 – 1.60 (m, 2H, CH_2). ^{13}C NMR (100 MHz , $CDCl_3$): δ = 191.4, 148.6, 140.9, 138.7, 135.7, 132.5, 128.6, 128.5, 118.9, 26.8, 24.5, 22.2, 22.1. HRMS (ESI) for $C_{15}H_{16}NaO$ [M+Na$^+$]: Calcd: 235.1093; Found: 235.1100. IR (KBr): 2925, 2854, 1582, 1650, 1444, 1290, 1122, 970, 831, 682 cm^{-1}.

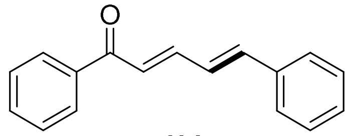

2bk

(*2E*, *4E*)-1,5-diphenylpenta-2,4-dien-1-one (2bk) (Armstrong et al., 2010) Prepared according to general procedure to afford as yellow solid (73% yield). R_f = 0.46 (EtOAc / hexanes 1 : 20); m. p. = 91 – 94 °C. ^{1}H NMR (400 MHz, $CDCl_3$): δ = 7.98 – 7.94 (m, 2H), 7.65 – 7.51 (m, 2H), 7.49 – 7.43 (m, 4H), 7.39 – 7.26 (m, 3H), 7.07 (d, J = 14.9 Hz, 1H), 7.01 – 6.93 (m, 2H). ^{13}C NMR (100 MHz, $CDCl_3$): δ = 190.5, 144.9, 142.0, 138.3, 136.1, 132.7, 129.3, 128.9, 128.6, 128.4, 127.4, 127.0, 125.5. HRMS (ESI) for $C_{17}H_{14}NaO$ [M+Na$^+$]: Calcd: 257.0937; Found: 257.0925. IR (KBr): 3060, 3029, 2958, 2928, 2597, 1963, 1903, 1717, 1682, 1657, 1578, 1284, 1253, 1010, 695 cm^{-1}.

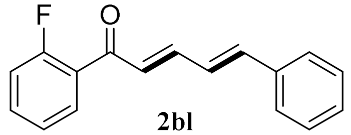

2bl

(2*E*, 4*E*)-1-(2-fluorophenyl)-5-phenylpenta-2,4-dien-1-one (2bl) Prepared according to general procedure to afford as yellow gum (77% yield). R_f = 0.42 (EtOAc / hexanes 1 : 20); ^{1}H NMR (400 MHz, $CDCl_3$): δ = 7.81 (td, J = 7.5, 1.8 Hz, 1H), 7.60 – 7.49 (m, 4H), 7.43 – 7.32 (m, 3H), 7.27 (td, J = 7.5, 0.9 Hz, 1H), 7.17 (ddd, J = 10.7, 8.3, 0.7 Hz, 1H), 7.05 – 7.01 (m, 2H), 6.97 (dd, J = 15.0, 2.8 Hz, 1H). ^{13}C NMR (100 MHz, $CDCl_3$): δ = 189.2, 161.2 (d, J = 252.9 Hz), 145.1, 142.5, 136.1,

133.9 (d, J = 8.7 Hz), 131.0 (d, J = 8.0 Hz), 129.4, 129.1 (d, J = 6.5 Hz), 129.0, 127.5, 127.3 (d, J = 13.4 Hz), 127.0, 124.6 (d, J = 3.4 Hz), 116.6 (d, J = 23.2 Hz). HRMS (ESI) for $C_{17}H_{13}FNaO$ [M+Na$^+$]: Calcd: 275.0843; Found: 275.0836. IR (KBr): 3062, 3030, 2926, 1655, 1609, 1579, 1282, 1012, 798, 692 cm^{-1}.

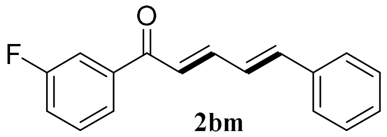

2bm

(2*E*, 4*E*)-1-(3-fluorophenyl)-5-phenylpenta-2,4-dien-1-one (2bm) Prepared according to general procedure to afford as yellow solid (74% yield). R_f = 0.36 (EtOAc / hexanes 1 : 20); m. p. = 52 – 56 °C. ^{1}H NMR (400 MHz, $CDCl_3$): δ = 7.74 (d, J = 7.7 Hz, 1H), 7.68 – 7.55 (m, 2H), 7.52 – 7.46 (m, 2H), 7.46 – 7.40 (m, 1H), 7.39 – 7.28 (m, 3H), 7.28 – 7.20 (m, 1H), 7.06 – 6.97 (m, 3H). ^{13}C NMR (100 MHz, $CDCl_3$): δ = 189.0 (d, J = 2.0 Hz), 162.9 (d, J = 247.7 Hz), 145.6, 142.6, 140.4 (d, J = 6.3 Hz), 136.0, 130.3 (d, J = 7.7 Hz), 129.5, 129.0, 127.5, 126.8, 124.8 124.1 (d, J = 2.9 Hz), 119.7 (d, J = 21.5 Hz), 115.3 (d, J = 22.3 Hz). HRMS (ESI) for $C_{17}H_{14}FO$ [M+H$^+$]: Calcd: 253.1023; Found: 253.1026. IR (KBr): 3070, 3029, 2922, 1680, 1658, 1581, 1486, 1262, 1001, 790 cm^{-1}.

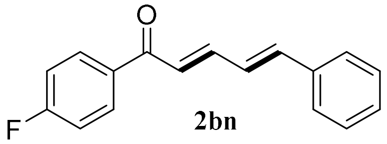

2bn

(2*E*, 4*E*)-1-(4-fluorophenyl)-5-phenylpenta-2,4-dien-1-one (2bn) (Polaquini et al., 2017) Prepared according to general procedure to afford as yellow solid (77% yield). R_f= 0.36 (EtOAc / hexanes 1 : 20); m. p. = 80 – 83 °C. ^{1}H NMR (400 MHz, $CDCl_3$): δ = 7.94 – 7.86 (m, 2H), 7.55 – 7.45 (m, 1H), 7.39 (d, J = 7.1 Hz, 2H), 7.31 – 7.18 (m, 3H), 7.08 – 7.02 (m, 2H), 6.96 (d, J = 14.9 Hz, 1H), 6.92 – 6.88 (m, 2H). ^{13}C NMR (100 MHz, $CDCl_3$): δ = 188.8, 165.6 (d, J = 254.2 Hz), 145.1, 142.3, 136.1, 134.6 (d, J = 3.0 Hz). 131.0 (d, J = 9.2 Hz), 129.4, 128.9, 127.4, 126.9, 124.9, 115.8 (d, J = 21.8 Hz). HRMS (ESI) for $C_{17}H_{13}FNaO$ [M+Na$^+$]: Calcd: 275.0843; Found: 275.0836. IR (KBr): 3067, 3024, 2959, 2924, 1652, 1602, 1502, 1250, 1002, 800, 619 cm^{-1}.

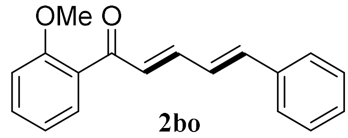

2bo

(2*E*, 4*E*)-1-(2-methoxyphenyl)-5-phenylpenta-2,4-dien-1-one (2bo) Prepared according to general procedure to afford as yellow gum (77% yield). R_f = 0.36 (EtOAc / hexanes 1 : 20); ^{1}H NMR (400 MHz, $CDCl_3$): δ = 7.58 (dd, J = 7.6, 1.7 Hz, 1H), 7.51 – 7.38 (m, 4H), 7.38 – 7.28 (m, 3H), 7.05 – 6.89 (m, 5H), 3.89 (s, 3H, OCH_3). ^{13}C NMR (100 MHz, $CDCl_3$): δ = 193.2, 158.1, 143.7, 141.3, 136.4, 132.8, 130.6, 130.4, 129.4, 129.2, 128.9, 127.3, 120.8, 111.7, 55.8. HRMS (ESI) for $C_{18}H_{16}NaO_2$ [M+Na$^+$]: Calcd: 287.1043; Found: 287.1051. IR (KBr): 3026, 3024, 2939, 1648, 1598, 1483, 1462, 1288, 1245, 1020, 755 cm^{-1}.

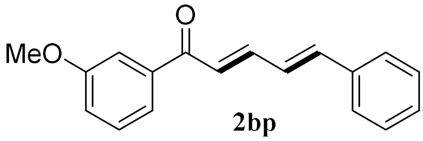

2bp

(2*E*, 4*E*)-1-(3-methoxyphenyl)-5-phenylpenta-2,4-dien-1-one (2bp) (Polaquini et al., 2017) Prepared according to general procedure to afford as yellow solid (76% yield). R_f= 0.46 (EtOAc / hexanes 1 : 10); m. p. = 71 – 75 °C. ^{1}H NMR (400 MHz, $CDCl_3$): δ = 7.64 – 7.49 (m, 3H), 7.49 – 7.42 (m, 2H), 7.40 – 7.25 (m, 4H), 7.08 (ddd, J = 8.4, 2.6, 0.8 Hz, 1H), 7.04 (d, J = 14.9 Hz, 1H), 7.02–6.91 (m, 2H), 3.82 (s, 3H, OCH_3). ^{13}C NMR (100 MHz, $CDCl_3$): δ = 190.0, 159.9, 144.8, 141.9, 139.6, 136.1, 129.5, 129.2, 128.8, 127.3, 126.9, 125.4, 120.9, 119.2, 112.7, 55.4. HRMS (ESI) for $C_{18}H_{16}NaO_2$ [M+Na$^+$]: Calcd: 287.1043; Found: 287.1051. IR (KBr): 3003, 2958, 2928, 1724, 1647, 1649, 1595, 1508, 1253, 1170, 1023, 998, 846, 691 cm^{-1}.

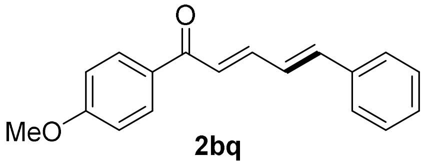

2bq

(2*E*, 4*E*)-1-(4-methoxyphenyl)-5-phenylpenta-2,4-dien-1-one (2bq) (Armstrong et al., 2010) Prepared according to general procedure to afford as pale yellow solid (77% yield). R_f= 0.55 (EtOAc / hexanes 1:10). m. p. = 55 – 58 °C. ^{1}H NMR (400 MHz, $CDCl_3$): δ = 8.00 (d, J = 8.8 Hz, 2H), 7.59 (ddd, J = 14.8, 9.0, 1.1 Hz, 1H), 7.48 (d, J

= 6.8 Hz, 2H), 7.39 – 7.26 (m, 3H), 7.10 (d, J = 14.9 Hz, 1H), 7.02 – 6.91 (m, 4H), 3.85 (s, 3H, OCH_3). ^{13}C NMR (100 MHz, $CDCl_3$): δ = 188.7, 163.4, 144.1, 141.5, 136.3, 131.2, 130.8, 129.2, 128.9, 127.3, 127.1, 125.3, 113.9, 55.5. HRMS (ESI) for $C_{18}H_{16}NaO_2$ [M+Na$^+$]: Calcd: 287.1043; Found: 287.1047. IR (KBr): 3052, 3003, 2958, 2929, 1597, 1356, 1257, 1023, 999, 845, 737 cm^{-1}.

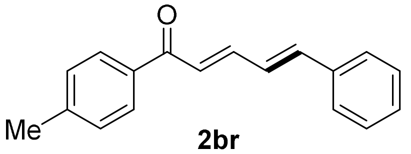

2br

(*2E*, *4E*)-5-phenyl-1-(p-tolyl)penta-2,4-dien-1-one (2br) (Polaquini et al., 2017) Prepared according to general procedure to afford as pale yellow solid (81% yield). R_f = 0.46 (EtOAc / hexanes 1 : 10); m. p. = 78 – 82 °C. ^1H NMR (400 MHz, $CDCl_3$): δ = 7.89 (d, J = 8.1 Hz, 2H), 7.58 (dd, J = 14.9, 9.5 Hz, 1H), 7.47 (d, J = 7.2 Hz, 2H), 7.39 – 7.28 (m, 3H), 7.26 (d, J = 8.0 Hz, 2H), 7.08 (d, J = 14.9 Hz, 1H), 7.04 – 6.91 (m, 2H), 2.39 (s, 3H, CH_3). ^{13}C NMR (100 MHz, $CDCl_3$): δ = 189.9, 144.4, 143.5, 141.7, 136.1, 135.6, 129.3, 129.2, 128.9, 128.6, 127.3, 127.0, 125.4, 21.7. HRMS (ESI) for $C_{18}H_{16}NaO$ [M+Na$^+$]: Calcd: 271.1093; Found: 271.1091. IR (KBr): 2921, 2856, 2732, 1673, 1444, 1251, 997, 802, 688 cm^{-1}.

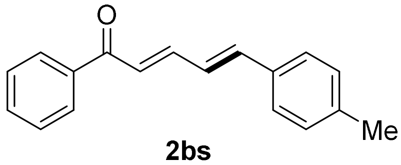

2bs

(*2E*, *4E*)-1-phenyl-5-(p-tolyl)penta-2,4-dien-1-one (2bs) (Armstrong et al., 2010) Prepared according to general procedure to afford as as yellow solid (81% yield). R_f= 0.47 (EtOAc / hexanes 1 : 20). m. p. = 62 – 65 °C. ^1H NMR (400 MHz, $CDCl_3$): δ = 7.97 (dd, J = 8.3, 1.2 Hz, 2H), 7.63 – 7.52 (m, 2H), 7.50 – 7.43 (m, 2H), 7.39 (d, J = 8.1 Hz, 2H), 7.17 (d, J = 7.9 Hz, 2H), 7.06 (d, J = 14.9 Hz, 1H), 7.00 – 6.95 (m, 2H), 2.35 (s, 3H, CH_3). ^{13}C NMR (100 MHz, $CDCl_3$) δ = 190.6, 145.3, 142.2, 139.6, 138.4, 133.5, 132.7, 130.0, 129.7, 128.7, 128.5, 127.4, 126.1, 125.0, 21.5. HRMS (ESI) for $C_{18}H_{17}O$ [M+ H$^+$]: Calcd: 249.1274; Found: 249.1282. IR (KBr): 3026, 2923, 2862, 2295, 1721, 1650, 1575, 1447, 1252, 1005, 842, 808, 692 cm^{-1}.

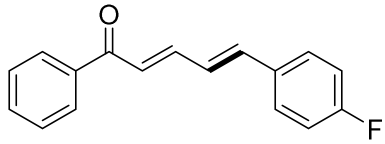

2bt

(*2E*, *4E*)-5-(4-fluorophenyl)-1-phenylpenta-2,4-dien-1-one (2bt) (Armstrong et al., 2010) Prepared according to general procedure to afford as as yellow solid (83% yield). R_f= 0.44 (EtOAc / hexanes 1 : 20). m. p. = 58 – 60 °C. ^{1}H NMR (400 MHz, $CDCl_3$): δ = 7.99 – 7.93 (m, 2H), 7.60 – 7.50 (m, 2H), 7.48 – 7.39 (m, 4H), 7.09 – 6.98 (m, 3H), 6.93 – 6.85 (m, 2H). ^{13}C NMR (100 MHz, $CDCl_3$): δ = 190.2, 163.2 (d, J = 250.0 Hz), 144.6, 140.5, 138.2, 132.7, 132.4 (d, J = 3.4 Hz), 129.0 (d, J = 8.2 Hz), 128.6, 128.4, 126.7 (d, J = 2.5 Hz), 125.4 (d, J = 0.9 Hz), 115.9 (d, J = 21.8 Hz). HRMS (ESI) for $C_{17}H_{14}FO$ [M+H$^+$]: Calcd: 253.1023; Found: 253.1013. IR (KBr): 3061, 2958, 2929, 2595, 1893, 1721, 1655, 1580, 1508, 1449, 1284, 1250, 1201, 1155, 1011, 1011, 844, 824, 694 cm^{-1}. Crystallographic data of **2bt** is available free of charge from the Cambridge Crystallographic Data Centre under accession number CCDC-1892057.

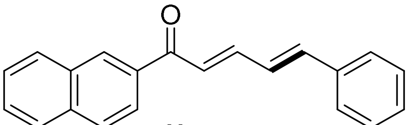

2bu

(*2E*, *4E*)-1-(naphthalen-2-yl)-5-phenylpenta-2,4-dien-1-one (2bu) (Polaquini et al., 2017) Prepared according to general procedure to afford as as yellow solid (78% yield). R_f= 0.45 (EtOAc / hexanes 1 : 20). m. p. = 104 – 106 °C. ^{1}H NMR (400 MHz, $CDCl_3$): δ = 8.50 (s, 1H), 8.07 (dd, J = 8.6, 1.7 Hz, 1H), 8.02 – 7.84 (m, 3H), 7.72 – 7.49 (m, 5H), 7.44 – 7.30 (m, 3H), 7.15 – 6.99 (m, 2H). ^{13}C NMR (100 MHz, $CDCl_3$) : δ = 190.4, 144.9, 142.1, 136.3, 135.7, 135.6, 132.7, 129.9, 129.7, 129.4, 129.0, 128.7, 128.5, 128.0, 127.5, 127.2, 126.9, 125.6, 124.6. HRMS (ESI) for $C_{21}H_{17}O$ [M+H$^+$]: Calcd: 285.1274; Found: 285.1261. IR (KBr): 3056, 3025, 2957, 2864, 1725, 1655, 1622, 1580, 1496, 1463, 1360, 1285,1149, 1000, 747, 693, cm^{-1}.

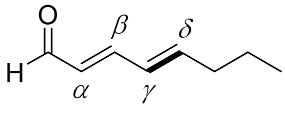

4aa

(2*E*, 4*E*)-octa-2,4-dienal (4aa) Prepared according to general procedure to afford as colourless oil liquid (81% yield). R_f= 0.49 (EtOAc / hexanes 1 : 30); ^{1}H NMR (400 MHz, $CDCl_3$): δ = 9.54 (d, J = 8.0 Hz, 1H, CHO), 7.09 (dd, J = 15.3, 9.7 Hz, 1H, β-H), 6.39 – 6.21 (m, 2H, γ-H and δ-H), 6.08 (dd, J = 15.4, 8.0 Hz, 1H, α-H), 2.25 – 2.15 (m, 2H, CH_2), 1.55 – 1.44 (m, 2H, CH_2), 0.95 (t, J = 7.4 Hz, 3H, CH_3). ^{13}C NMR (100 MHz, $CDCl_3$): δ = 194.2, 153.1, 147.3, 130.2, 128.9, 35.3, 21.9, 13.8. HRMS (ESI) for $C_8H_{12}NaO$ [M+Na$^+$]: Calcd: 147.0780; Found: 147.0789. IR (KBr): 2923, 2854, 1681, 1459, 1374, 1116, 792 cm^{-1}.

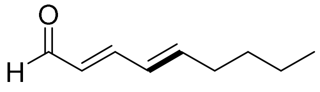

4ab

(2*E*, 4*E*)-nona-2,4-dienal (2ab) (Kelly et al., 2015) Prepared according to general procedure to afford as colourless oil liquid (83% yield). R_f= 0.47 (EtOAc / hexanes 1 : 30). ^{1}H NMR (400 MHz, $CDCl_3$): δ = 9.53 (d, J = 8.0 Hz, 1H, CHO), 7.10 (dd, J = 15.2, 10.0 Hz, 1H, β-H), 6.36 – 6.24 (m, 2H, γ-H and δ-H), 6.08 (dd, J = 15.3, 8.0 Hz, 1H, α-H), 2.25 – 2.17 (m, 2H, CH_2), 1.50 – 1.40 (m, 2H, CH_2), 1.40 – 1.30 (m, 2H, CH_2), 0.92 (t, J = 7.2 Hz, 3H, CH_3). ^{13}C NMR (100 MHz, $CDCl_3$) δ = 194.1, 153.0, 147.5, 130.1, 128.8, 33.0, 30.8, 22.4, 14.0. HRMS (ESI) for $C_9H_{14}NaO$ [M+Na$^+$]: Calcd: 161.0937; Found: 161.0945. IR (KBr): 2929, 2865, 1687, 1637, 1164, 1118, 987, 734 cm^{-1}.

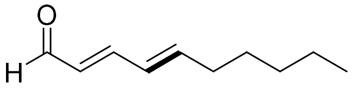

4ac

(2*E*, 4*E*)-deca-2,4-dienal (4ac) (Holan et al., 2014) Prepared according to general procedure to afford as colorless oil (82% yield). R_f= 0.55 (EtOAc / hexanes 1 : 30). ^{1}H NMR (400 MHz, $CDCl_3$): δ = 9.54 (d, J = 8.0 Hz, 1H, CHO), 7.09 (dd, J = 15.2, 9.6 Hz, 1H, β-H), 6.44 – 6.22 (m, 2H, γ-H and δ-H), 6.08 (dd, J = 15.3, 8.0 Hz, 1H, α-H), 2.24 – 2.16 (m, 2H, CH_2), 1.51 – 1.42 (m, 2H, CH_2), 1.39 – 1.18 (m, 4H, $2CH_2$), 0.90 (t, J = 7.0 Hz, 3H, CH_3). ^{13}C NMR (100 MHz, $CDCl_3$): δ = 194.1, 153.1, 147.6, 130.2, 128.8, 33.3, 31.5, 28.4, 22.6, 14.1. HRMS (ESI) for $C_{10}H_{17}O$ [M+H$^+$]: Calcd:

153.1274; Found: 153.1280. IR (KBr): 2923, 2856, 1671, 1085, 624 cm^{-1}.

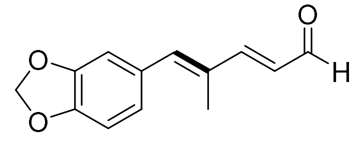

4ae

(*2E*, *4E*)-5-(benzo[d][1,3]dioxol-5-yl)-4-methylpenta-2,4-dienal (4ae) (Riveira et al., 2012) Prepared according to general procedure to afford as yellow solid (85% yield). R_f= 0.51 (EtOAc / hexanes 1 : 4). m. p. = 70 – 71 °C. ^{1}H NMR (400 MHz, $CDCl_3$): δ = 9.53 (d, J = 7.8 Hz, 1H, CHO), 7.17 (d, J = 15.5, 1H, β-H), 6.83 – 6.66 (m, 4H, Ar-H and δ-H), 6.15 (dd, J = 15.5, 7.8 Hz, 1H, α-H), 5.91 (s, 2H, CH_2), 1.99 (s, 3H, CH_3). ^{13}C NMR (100 MHz, $CDCl_3$) : δ = 194.0, 158.4, 147.8, 147.82, 140.78, 133.1, 130.4, 127.7, 124.7, 109.5, 108.5, 101.4, 14.0. HRMS (ESI) for $C_{13}H_{13}O_3$ [M+H$^+$]: Calcd: 217.0859; Found: 217.0862. IR (KBr): 2913, 1673,1599, 1495, 1444, 1359, 1296,1253, 1195,1154, 1035, 983, 798 cm^{-1}.

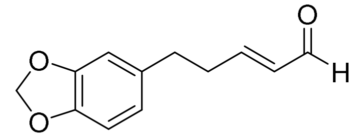

3ad

(*E*)-5-(benzo[d][1,3]dioxol-5-yl)pent-2-enal (3ad) R_f = 0.51 (EtOAc / hexanes 1 : 10); ^{1}H NMR (400 MHz, $CDCl_3$): δ = 9.49 (d, J = 7.8 Hz, 1H, CHO), 6.90 – 6.78 (m, 1H), 6.77 – 6.59 (m, 3H), 6.12 (dd, J = 15.6, 7.8 Hz, 1H), 5.94 (s, 2H, OCH_2O), 2.85 – 2.70 (m, 2H, CH_2), 2.70 – 2.52 (m, 2H, CH_2). ^{13}C NMR (100 MHz, $CDCl_3$): δ = 194.1, 157.4, 147.9, 146.1, 134.1, 133.5, 121.3, 108.8, 108.4, 101.0, 34.6, 33.9. HRMS (ESI) for $C_{12}H_{12}O_3Na$ [M+Na$^+$]: Calcd: 227.0679; Found: 227.0683. IR KBr): 2362, 1681, 1488, 1239, 1117, 1032, 919, 804, 742 cm^{-1}.

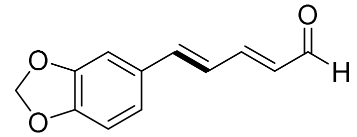

4ad

(*2E*,*4E*)-5-(benzo[d][1,3]dioxol-5-yl)penta-2,4-dienal (4ad) (Chandrasekhar et al., 2000) Prepared according to general procedure to afford as yellow solid (91% yield). R_f= 0.45 (EtOAc / hexanes 1 : 10). m. p. = 77 – 78 °C. ^{1}H NMR (400 MHz, $CDCl_3$): δ

= 9.59 (d, J = 8.0 Hz, 1H, CHO), 7.24 (dd, J = 14.9, 10.4 Hz, 1H, β-H), 7.08 – 6.75 (m, 5H, Ar-H and γ-H and δ-H), 6.23 (dd, J = 15.1, 8.0 Hz, 1H, α-H), 6.01 (s, 2H, OCH_2O). ^{13}C NMR (100 MHz, $CDCl_3$) : δ = 193.7, 152.5, 149.3, 148.5, 142.4, 131.0, 130.2, 124.6, 123.8, 108.8, 106.2, 101.7. HRMS (ESI) for $C_{12}H_{10}O_3Na$ [M+Na$^+$]: Calcd: 225.0522; Found: 225.0516. IR (KBr): 2914, 1675, 1599, 1495, 1444, 1359, 1296, 1253, 1195, 1154, 1035, 983, 797 cm^{-1}.

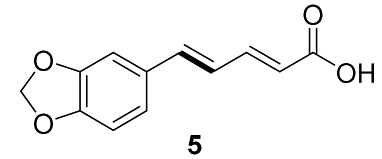

5

(*2E*,*4E*)-5-(benzo[d][1,3]dioxol-5-yl)penta-2,4-dienoic acid (5) Prepared according to previous literature's procedure (Chandrasekhar et al., 2000) to afford as yellow solid (83% yield). R_f= 0.40 (EtOAc / hexanes 1 : 4). m. p. = 194 – 195 °C. ^1H NMR (400 MHz, DMSO-d_6): δ = 12.29 (br, 1H, COOH), 7.31 (ddd, J = 15.2, 6.6, 3.7 Hz, 1H, β-H), 7.24 (d, J = 1.2 Hz, 1H, Ar-H), 7.05 – 6.88 (m, 4H, Ar-H and γ-H and δ-H), 6.06 (s, 2H, CH_2), 5.93 (d, J = 15.2 Hz, 1H, α-H). ^{13}C NMR (100 MHz, DMSO-d_6) : δ = 168.1, 148.6, 148.5, 145.1, 140.3, 131.0, 125.3, 123.6, 121.6, 109.0, 106.2, 101.8. HRMS (ESI) for $C_{12}H_{10}O_4Na$ [M+Na$^+$]: Calcd: 241.0471; Found: 241.0482. IR (KBr): 2921, 2544, 1679, 1601, 1459, 1449, 1368, 1309, 1257, 1193, 1148, 1104, 1035, 998, 930, 851, 797, 607 cm^{-1}.

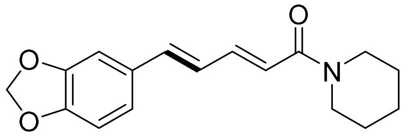

Piperine

Piperine Prepared according to previous literature's procedure (Chandrasekhar et al., 2000) to afford as pale yellow solid (71% yield). R_f= 0.42 (EtOAc / hexanes 1 : 10). m. p. = 121 – 122 °C. ^1H NMR (400 MHz, $CDCl_3$): δ = 7.40 (ddd, J = 14.6, 7.3, 2.9 Hz, 1H, β-H), 6.97 (s, 1H, Ar-H), 6.88 (d, J = 8.0 Hz, 1H, Ar-H), 6.81 – 6.71 (m, 3H, Ar-H and γ-H and δ-H), 6.44 (d, J = 14.7 Hz, 1H, α-H), 5.96 (s, 2H, CH_2), 3.57 (d, J = 44.1 Hz, 4H, $2CH_2$), 1.62 (dd, J = 28.2, 4.5 Hz, 6H, $3CH_2$). ^{13}C NMR (100 MHz, $CDCl_3$) : δ = 165.4, 148.2, 148.1, 142.5, 138.2, 131.0, 125.4, 122.5, 120.1, 108.5,

105.7, 101.3, 46.9, 43.3, 26.8, 25.7, 24.7. HRMS (ESI) for $C_{17}H_{20}NO_3$ [$M+H^+$]: Calcd: 286.1438; Found: 286.1447. IR (KBr): 3007, 2938, 1634, 1583, 1489, 1444, 1363, 1252, 1194, 1132, 1027, 926, 850 cm^{-1}.

3. General Procedure for the Mechanistic Experiments

3.1. Parallel experiments for k_H / k_D

Deuterated-substratep reparation: Synthesis of [5,5-d_2] (*E*)-6-phenylhex-3-en-2-one (**S1-*d*2**)

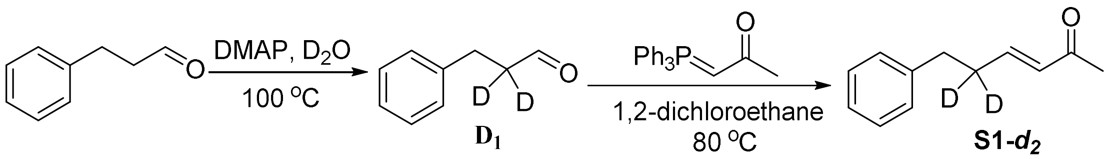

Figure S1 Synthesis of substrate **S1-*d*2**, related to **Scheme 4**

[2,2-*d*2]-3-phenylpropanal (D_1). (Ariza et al., 2010) Hydrocinnamaldehyde (1 mL) with D_2O (1 mL) was heated to 100 °C in the presence of 4-(N,N-dimethylamino)pyridine (4-DMAP, 13 mg) for 1 h in a septum-sealed flask. Thus, CH_2Cl_2 (4 mL) and 1M aq. HCl (1 mL) were added to the mixture at room temperature. The organic layer was then washed with aq. $NaHCO_3$ and brine.The organic layer was dried ($MgSO_4$) and the solvent was carefully removed. The crude product was shown to be 99 % deuterated material, and was distilled in vacuo to yield **D_1** (98% yield, 99% D) as a colorless oil. ^{1}H NMR (400 MHz, $CDCl_3$) : δ = 9.85 (s, 1H, CHO), 7.39 – 7.34 (m, 2H, Ar-H), 7.30 – 7.23 (m, 3H, Ar-H), 3.00 (s, 2H). ^{13}C NMR (100 MHz, $CDCl_3$) : δ = 201.9, 140.4, 128.7, 128.4, 126.4, 45.1 – 44.4 (m), 28.0. HRMS (ESI) for $C_9H_9D_2NaO$ [M+Na$^+$]: Calcd: 159.0749; Found: 159.0755. IR (KBr): 3060, 3027, 2927, 2859, 2723, 1722, 1495, 1450, 745, 700 cm^{-1}.

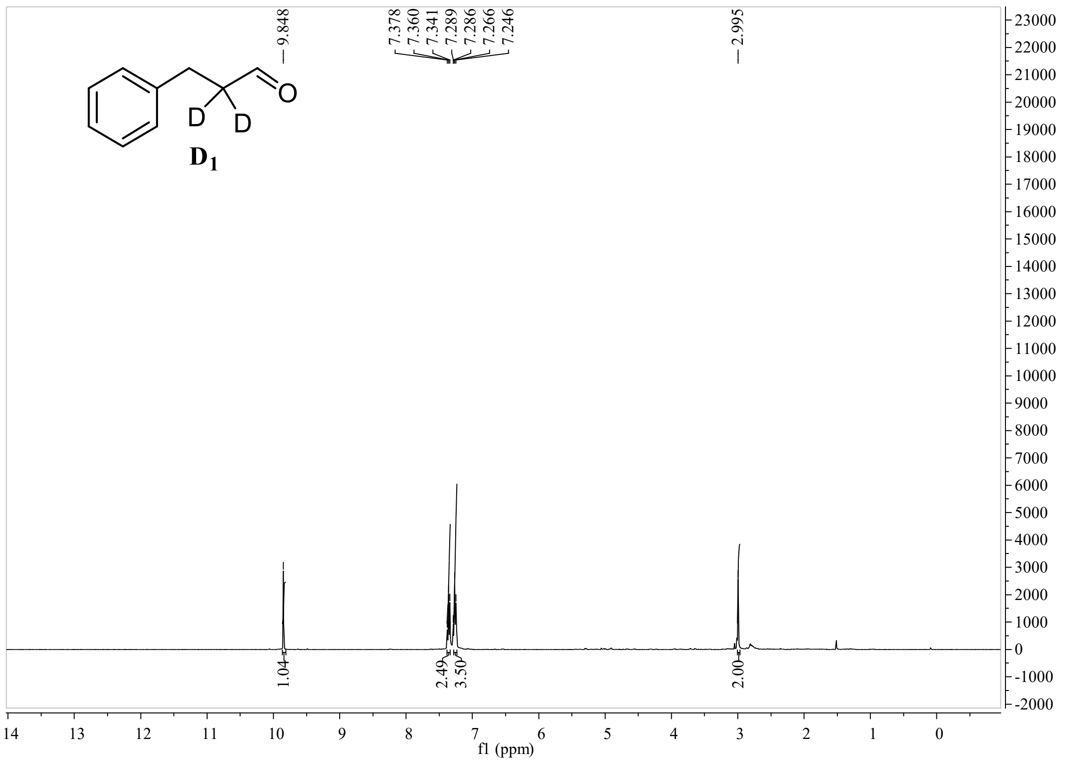

Figure S2 ^{1}H NMR spectrum of compound $\mathbf{D_1}$, related to **Scheme 4**

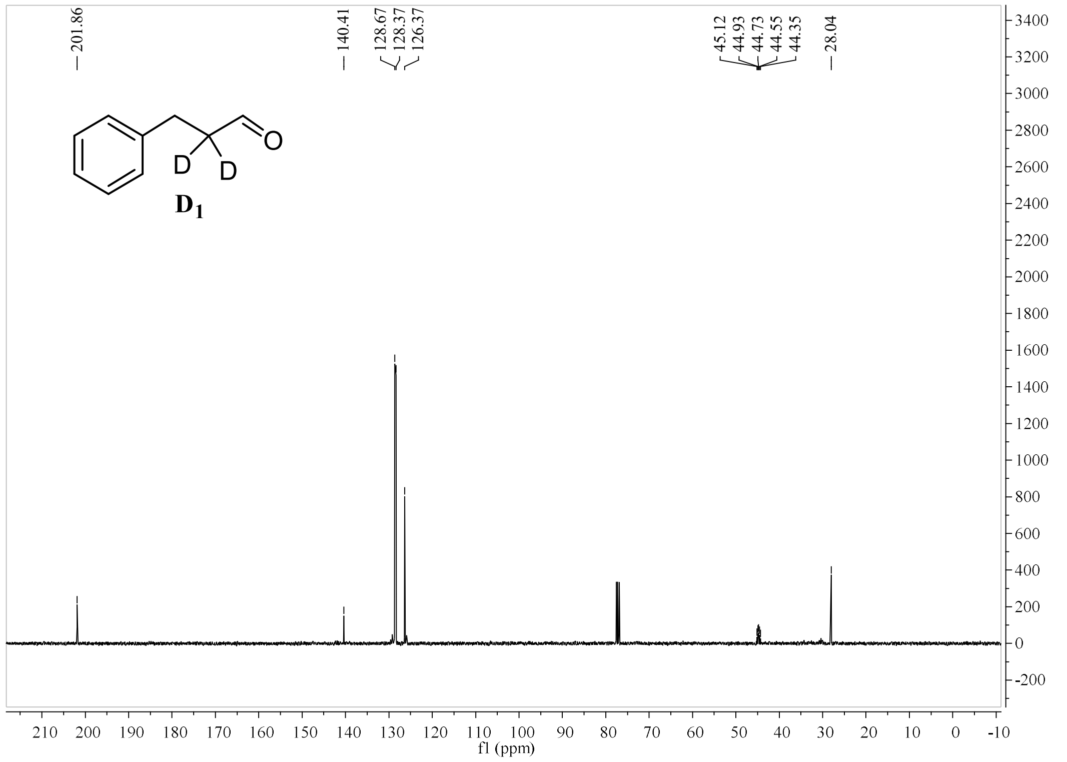

Figure S3 ^{13}C NMR spectrum of compound $\mathbf{D_1}$, related to **Scheme 4**

[5,5-d$_2$]-(E)-6-phenylhex-3-en-2-one (S1-*d$_2$*). (Shih et al., 2015) A mixture of (acetylmethylene)Triphenylphosphorane (1 g, 318, 3.3 mmol) and **D1** (408 mg, 3.0

mmol) was heated at 80 °C in a two necked round bottom flask using 1,2-dichloroethane (10.0 mL) as solvent for 16 hour. The completion of the reaction was monitored by TLC. Then solvent was evaporated in rotary evaporator under reduced pressure to obtain the residue. The residue was purified by flash column chromatography on silica gel to isolate the product. The product was obtained colorless liquid. (83% yield, 99% D). ^{1}H NMR (400 MHz, $CDCl_3$) : δ = 7.30 – 7.22 (m, 2H, Ar-H), 7.21 – 7.11 (m, 3H, Ar-H), 6.78 (d, J = 16.0 Hz, 1H, β-H), 6.06 (d, J = 16.0 Hz, 1H, α-H), 2.74 (s, 2H, CH_2), 2.19 (s, 3H, CH_3). ^{13}C NMR (100 MHz, $CDCl_3$) : δ = 198.4, 146.9, 140.5, 131.7, 128.4, 128.2, 126.1, 34.1, 34.0 – 33.1 (m), 26.7. HRMS (ESI) for $C_{12}H_{12}D_2NaO$ [M+Na$^+$]: Calcd: 199.1062; Found: 199.1057. IR (KBr): 3309, 3059, 3027, 2926, 2859, 1673, 1626, 1360, 1257, 988, 743, 701 cm^{-1}.

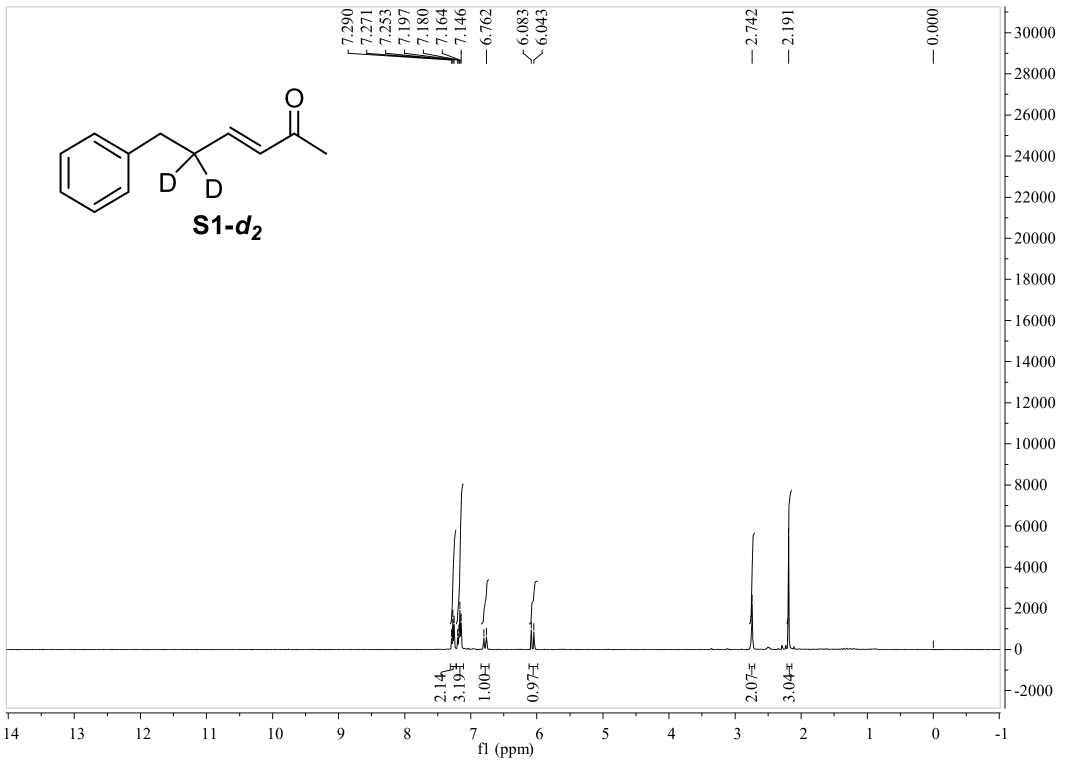

Figure S4 ^{1}H NMR spectrum of compound **S1-d_2**, related to **Scheme 4**

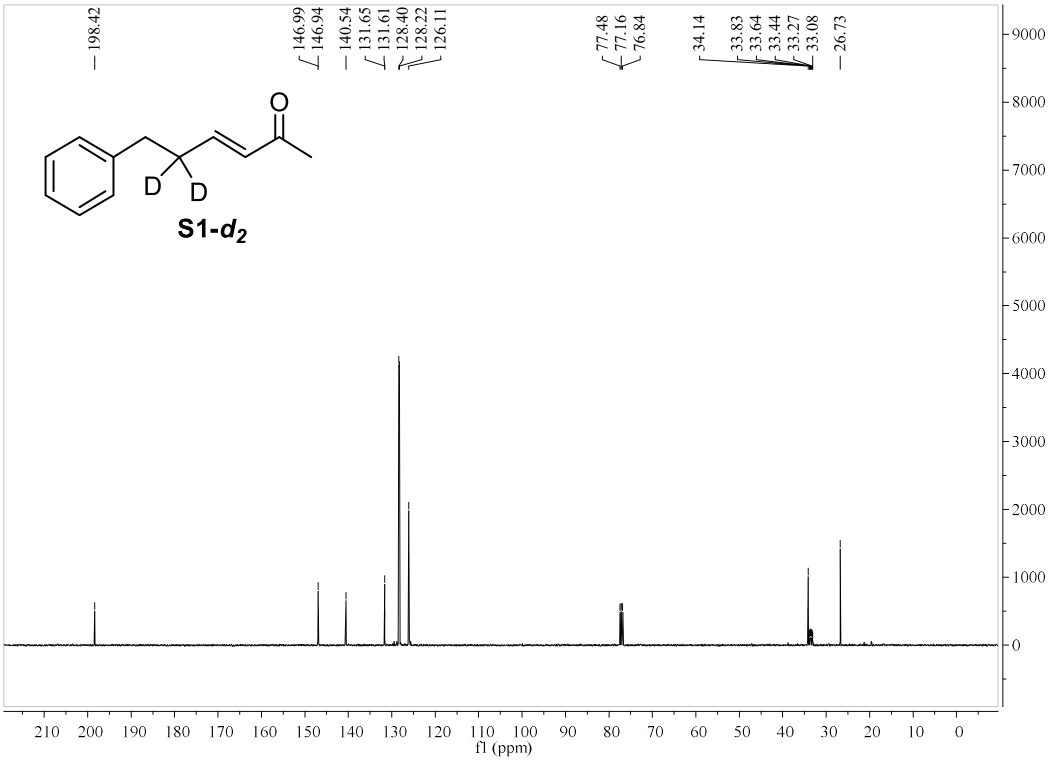

Figure S5 ^{1}H NMR spectrum of compound **S1-*d₂***, related to **Scheme 4**

KIE experiment: We use **1an** and deuterated **S1-*d_2*** as starting materials and two parallel sets reactions at standard conditions. The conversion was measured by ^{1}H NMR analysis for five times (30 min, 60 min, 90 min, 120 min, 150 min) to compare the initial reaction rates.

Table S1. Conversion (%) of the reaction of **1an** (*α*-H_2 and *α*-D_2), related to **Scheme 4**

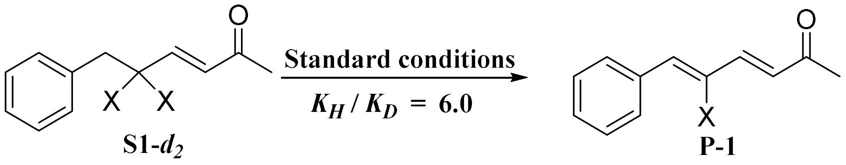

reaction time (min)	H-Conversion (%)	D-Conversion (%)
30	12.8%	2.9%
60	18.9%	3.8%
90	26.2%	6.0%
120	35.8%	6.5%
150	40.2%	7.7%

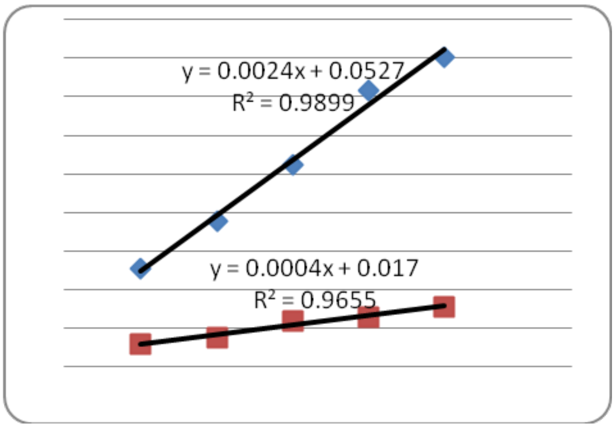

Figure S6. Conversion (%) versus Time (min), related to **Scheme 4**

3.2. Intramolecular competitive reaction for K_H / K_D

Deuterated-substrate preparation (Diao et al., 2012)**:** Synthesis of [6-d] (*E*)-6-phenylhex-3-en-2-one (**S2-*d₁***)

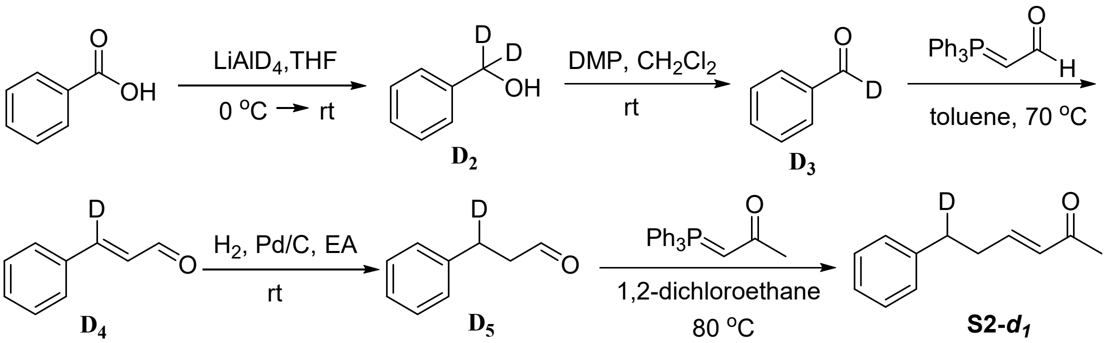

Figure S7. Synthesis of substrate **S2-*d₁***, related to **Scheme 4**

[1,1-*d₂*]-Benzyl alcohol (D_2). (Diao et al., 2012) An oven-dried 250 mL round bottom flask was equipped with a stir bar. After purging with N_2, the flask was charged with $LiAlD_4$ (513 mg, 13.5 mmol, 1.1 equiv) and 50 mL THF and stirred at 0 °C. A oven-dried 100 mL round bottom flask equipped with stir bar and septum was purged with N_2. A solution of benzoic acid (1.50 g, 12.3 mmol, 1 equiv) in 50 mL THF were added to the 100 mL flask and stirred at 0 °C. Above benzoic acid solution were added dropwise to the $LiAlD_4$ suspension at 0 °C using a cannula. The reaction mixture was slowly warmed to room temperature and allowed stirring for overnight. The mixture was diluted to 2 × its original volume with ethyl acetate and quenched by dropwise addition of water at 0 °C. The mixture was extracted with diethyl ether (3 × 50 mL), and the combined organic layers were washed with brine (2 × 50 mL), dried

over Na_2SO_4, and concentrated by evaporation. Distillation in vacuo yielded **D_2** (45%, 99% D) as a colorless oil. ^1H NMR data match previously reported data. ^1H NMR (400 MHz, $CDCl_3$) : δ = 7.41 – 7.16 (m, 5H, Ar-H), 3.74 (br, 1H, OH). ^{13}C NMR (100 MHz, $CDCl_3$) : δ = 140.7, 128.6, 127.7, 127.1, 64.4-60.6 (m). HRMS (ESI) for $C_7H_6D_2NaO$ [M+Na$^+$]: Calcd: 133.0593; Found: 133.0600. IR (KBr): 3325, 3027, 2197, 2135, 2085, 1494, 1447, 1227, 1095, 1058, 1024, 965, 921, 716 cm^{-1}.

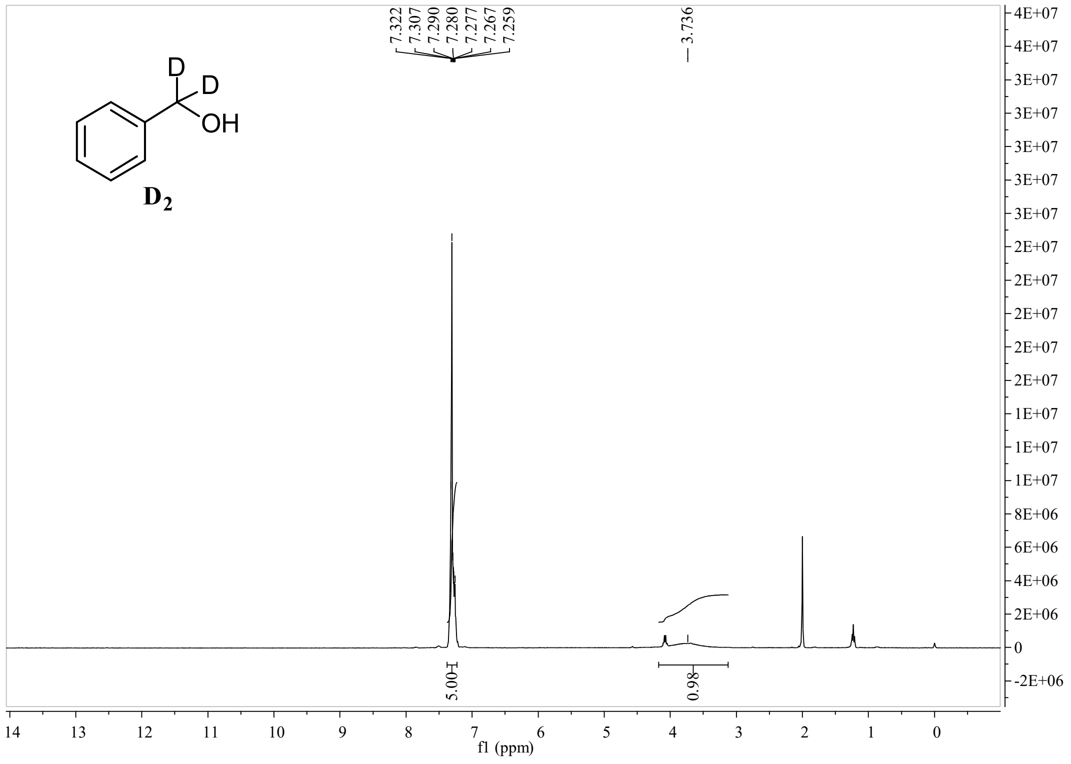

Figure S8 ^1H NMR spectrum of compound **D_2**, related to **Scheme 4**

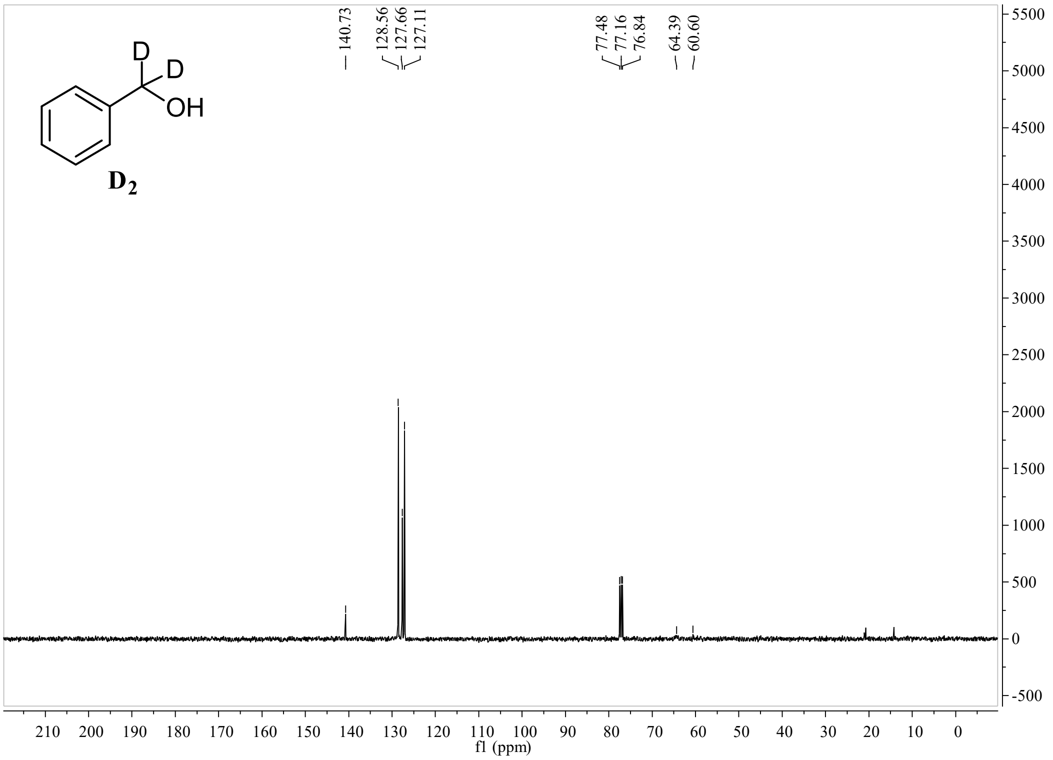

Figure S9 ^{13}C NMR spectrum of compound **D$_2$**, related to **Scheme 4**

[1-*d$_1$*]-Benzaldhyde (D$_3$) (Diao et al., 2012) A 100 mL round bottom flask equipped with a stir bar was charged with 20 mL CH_2Cl_2 and **D$_2$** (380 mg, 3.44 mmol). Then DMP (1,1,1-Triacetoxy-1,1-Dihydro-1,2-Benziodoxol-3(1H)-One (2.19 g, 5.16 mmol, 1.5 equiv.) were added with stirring. The solution was stirred at room temperature and consumption of starting material was monitored by TLC. Upon reaction completion, the reaction mixture was filtered through a plug of silica. Removal of solvent afforded **D$_3$** (90% yield, 99% D) as a colorless oil. ^{1}H NMR data match previously reported data. ^{1}H NMR (400 MHz, $CDCl_3$) : δ = 7.89 (d, J = 8.1 Hz, 2H, Ar-H), 7.67 – 7.57 (m, 1H, Ar-H), 7.56 – 7.47 (m, 2H, Ar-H). ^{13}C NMR (100 MHz, $CDCl_3$) : δ =187.4 (t, J = 26 Hz), 131.5, 129.7, 125.0, 124.2. HRMS (ESI) for C_7H_6DO [M+H$^+$]: Calcd: 108.0554; Found: 108.0553. IR (KBr): 3061, 3030, 2989, 2937, 2827, 2595, 1687, 1450, 1266, 1113, 1083, 1031, 736, 697 cm^{-1}.

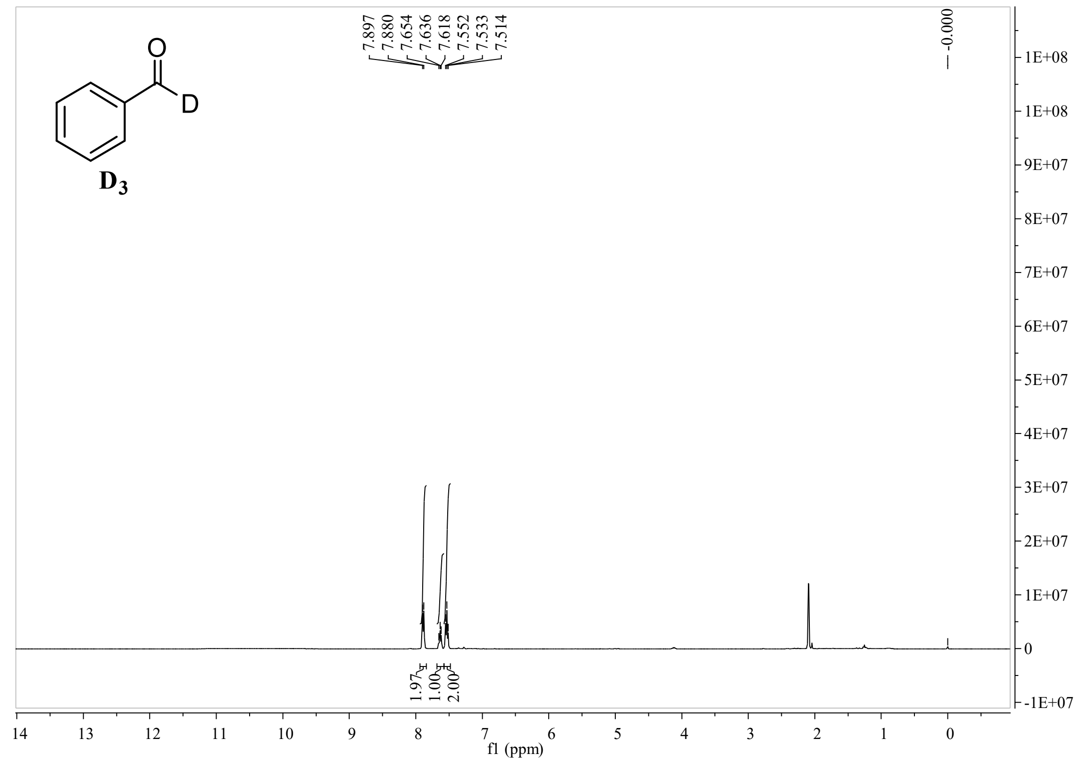

Figure S10 ^{1}H NMR spectrum of compound **D$_3$**, related to **Scheme 4**

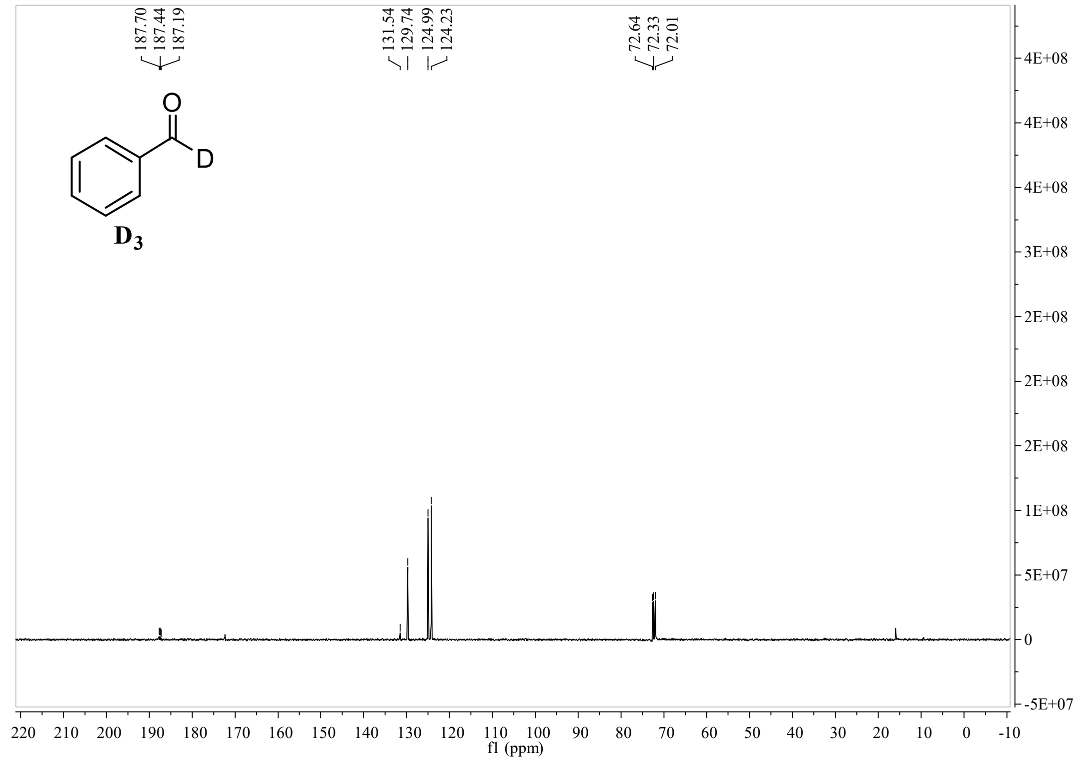

Figure S11 ^{13}C NMR spectrum of compound **D$_3$**, related to **Scheme 4**

[3-d_1]-cinnamaldehyde (D$_4$) (Diao et al., 2012) A mixture of (acetaldehyde)Triphenylphosphoranylidene (1 g, 3.3 mmol) and **D$_3$** (321 mg, 3.0

mmol) was heated at 80 °C in a two necked round bottom flask using 1,2-dichloroethane (10.0 mL) as solvent for 16 hour. The completion of the reaction was monitored by TLC. Then solvent was evaporated in rotary evaporator under reduced pressure to obtain the residue. The residue was purified by flash column chromatography on silica gel to isolate the product. The product was obtained colorless liquid (52% Yield, 99% D). ^{1}H NMR (400 MHz, $CDCl_3$) : δ = 9.71 (d, *J* = 7.7 Hz, 1H, CHO), 7.60 – 7.54 (m, 2H, Ar-H), 7.47 – 7.41 (m, 3H, Ar-H), 6.78 – 6.68 (m, 1H, *α*-H). ^{13}C NMR (100 MHz, $CDCl_3$) : δ = 193.8, 152.5 (t, *J* = 23.0 Hz), 134.0, 131.3, 129.1 (2C), 128.5 (3C). HRMS (ESI) for C_9H_7DNaO [M+Na^+]: Calcd: 156.0530; Found: 156.0535. IR (KBr): 3058, 3026, 2970, 2828, 2727, 2614, 1676, 1612, 1149, 891, 691 cm^{-1}.

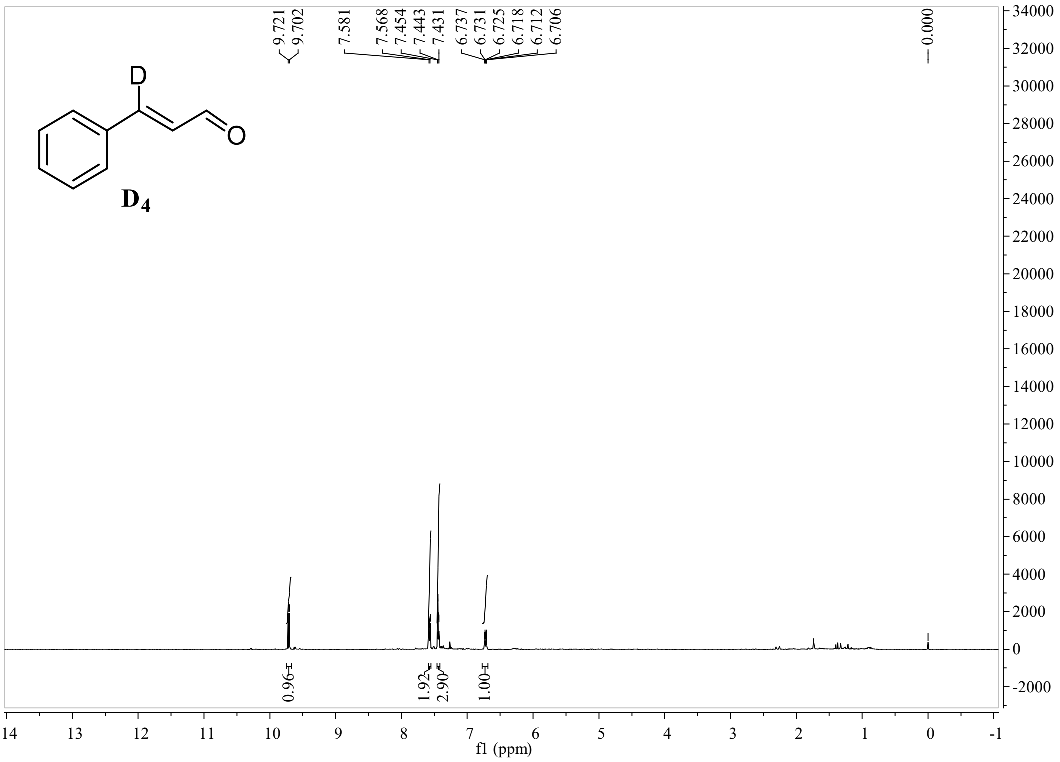

Figure S12 ^{1}H NMR spectrum of compound **D$_4$**, related to **Scheme 4**

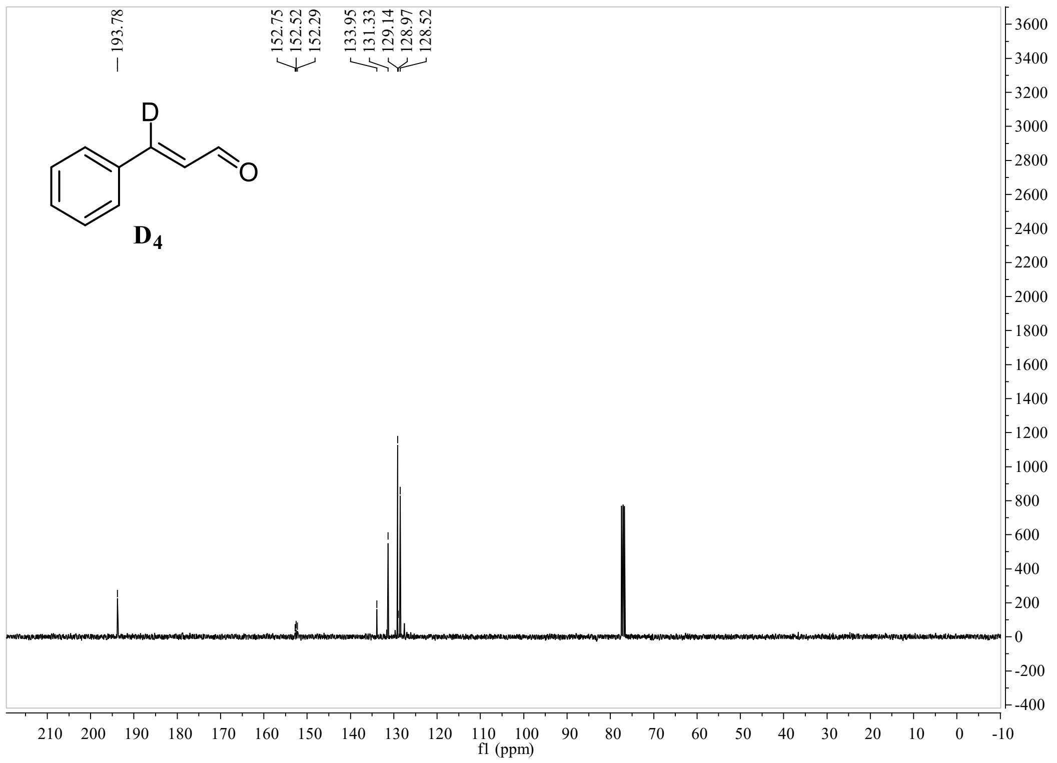

Figure S13 ^{13}C NMR spectrum of compound **D_4**, related to **Scheme 4**

[3-d_1]-3-phenylpropanal (D_5) (Diao et al., 2012) A 50 mL round bottom flask equipped with stir bar and septum was charged with EtOAc (10 mL), **D_4** (211.5 mg, 1.5 mmol), and 5% Pd/C (10 wt % of the substrate). A balloon was purged three times with H_2, and introduced into the reaction via a needle. The reaction was allowed to stir at room temperature under H_2 (1 atm) for 2 h. Filtration through a plug of celite and the solvent was removed in vacuo and the crude material was purified by column chromatography using a 10% EtOAc in hexane gradient to afford **D_5** as a colorless liquid (82% yield, 99% D). ^{1}H NMR (400 MHz, $CDCl_3$) : δ = 9.79 (s, 1H, CHO), 7.32 – 7.24 (m, 2H, Ar-H), 7.23 – 7.17 (m, 3H, Ar-H), 2.92 (t, J = 7.4 Hz, 1H, β-H), 2.75 (d, J = 7.6 Hz, 2H, α-H). ^{13}C NMR (100 MHz, $CDCl_3$) : δ = 201.6, 140.4, 128.6, 128.3, 126.3, 45.2, 27.8 (t, J = 20.0 Hz). HRMS (ESI) for C_9H_9DNaO [M+Na$^+$]: Calcd: 158.0687; Found: 158.0683. IR (KBr): 3060, 3027, 2929, 2724, 1710, 1495, 1286, 792, 700 cm^{-1}.

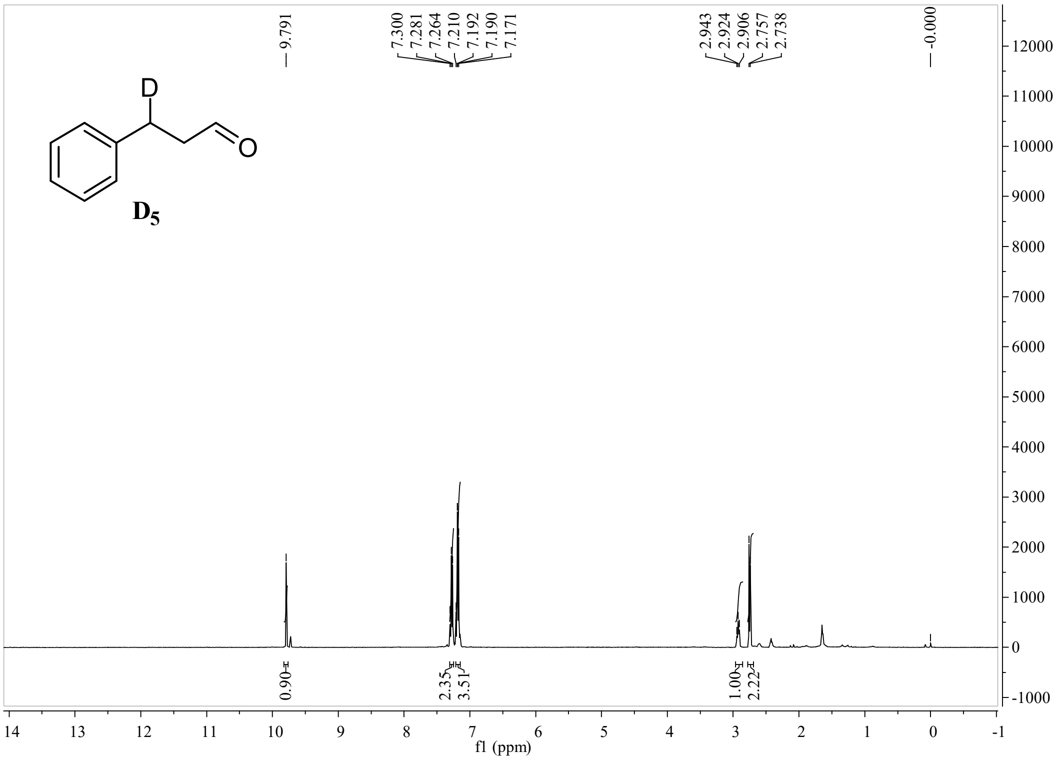

Figure S14 ^{1}H NMR spectrum of compound **D_5**, related to **Scheme 4**

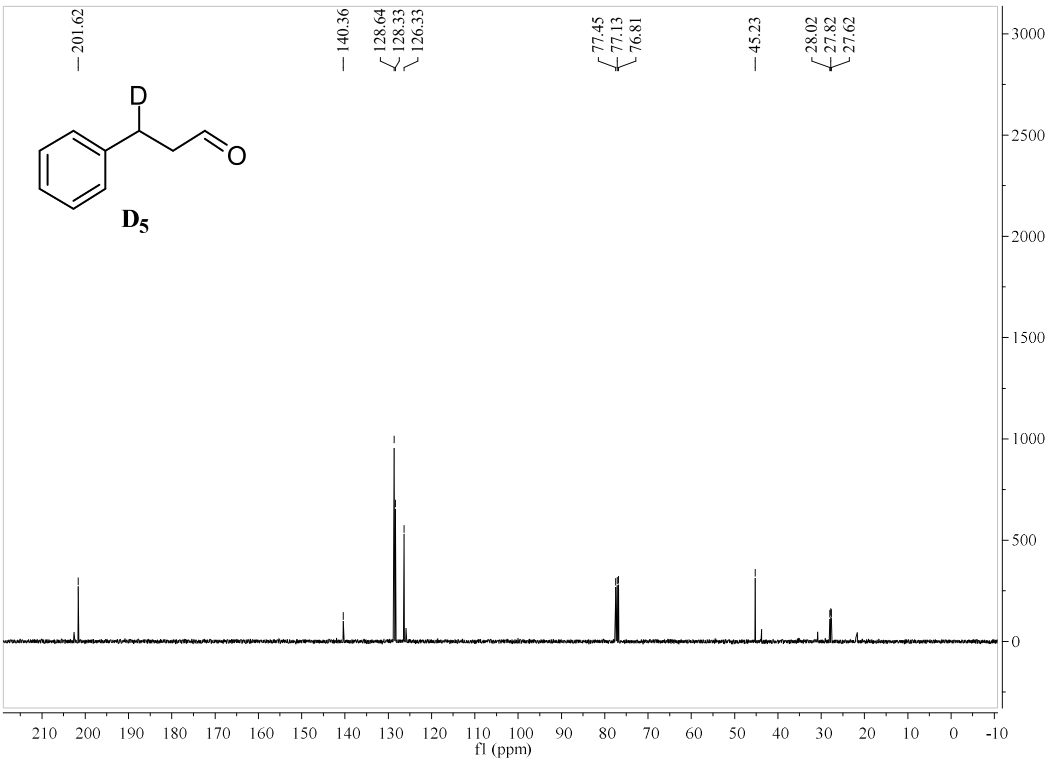

Figure S15 ^{1}H NMR spectrum of compound **D_5**, related to **Scheme 4**

[6-*d_1*]-(*E*)-6-phenylhex-3-en-2-one (S2-*d_1*) (Diao et al., 2012) A mixture of (acetylmethylene)Triphenylphosphorane (1 g, 3.3 mmol) and **D_3** (321 mg, 3.0 mmol)

was heated at 80 °C in a two necked round bottom flask using 1,2-dichloroethane (10.0 mL) as solvent for 16 hour. The completion of the reaction was monitored by TLC. Then solvent was evaporated in rotary evaporator under reduced pressure to obtain the residue. The residue was purified by flash column chromatography on silica gel to isolate the product. The product was obtained colorless liquid. (92% Yield, 99% D). ^{1}H NMR (400 MHz, $CDCl_3$) : δ = 7.33 – 7.24 (m, 2H, Ar-H), 7.23 – 7.12 (m, 3H, Ar-H), 6.81 (dt, *J* = 15.6, 6.8 Hz, 1H, *β*-H), 6.08 (d, *J* = 15.9 Hz, 1H, *α*-H), 2.75 (t, *J* = 7.4 Hz, 1H, CHD), 2.56 – 2.47 (m, 2H, CH_2), 2.21 (s, 3H, CH_3). ^{13}C NMR (100 MHz, $CDCl_3$) : δ = 198.6, 147.1, 140.6, 131.7, 128.5, 128.3, 126.2, 34.3 – 33.7 (m, 2C), 26.9. HRMS (ESI) for $C_{12}H_{13}DNaO$ [M+Na$^+$]: Calcd: 198.1000; Found: 198.0996. IR (KBr): 3313, 3058, 3026, 2926, 1673, 1628, 1360, 1255, 979, 741, 700 cm^{-1}.

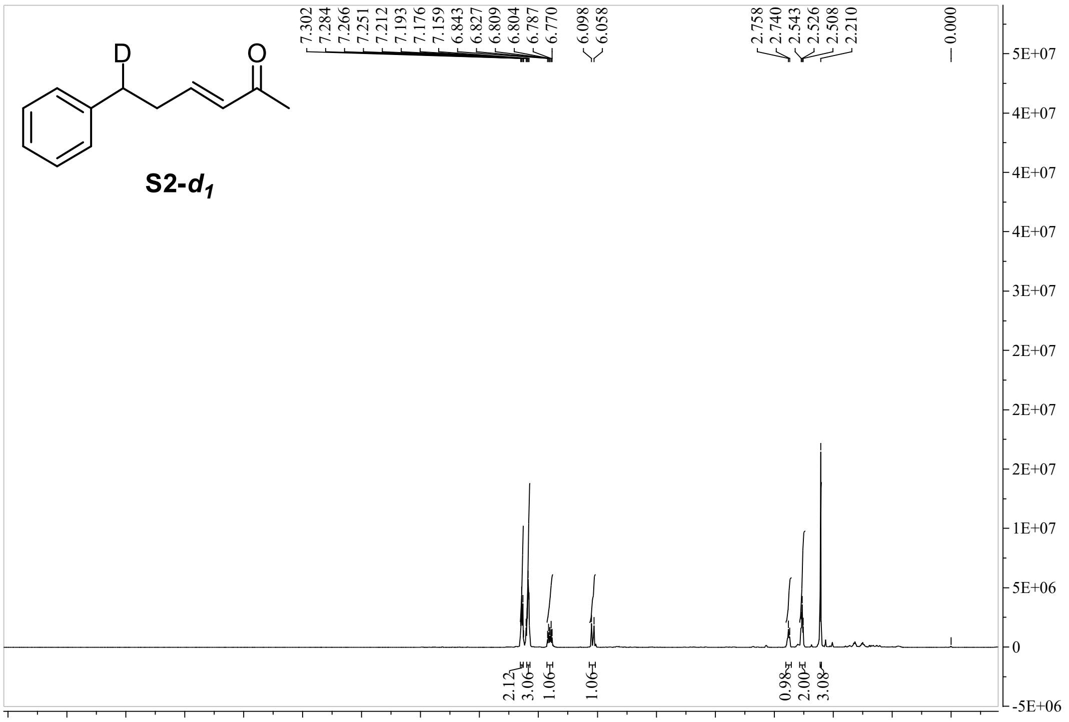

Figure S16 ^{1}H NMR spectrum of compound **S2-*d*$_1$**, related to **Scheme 4**

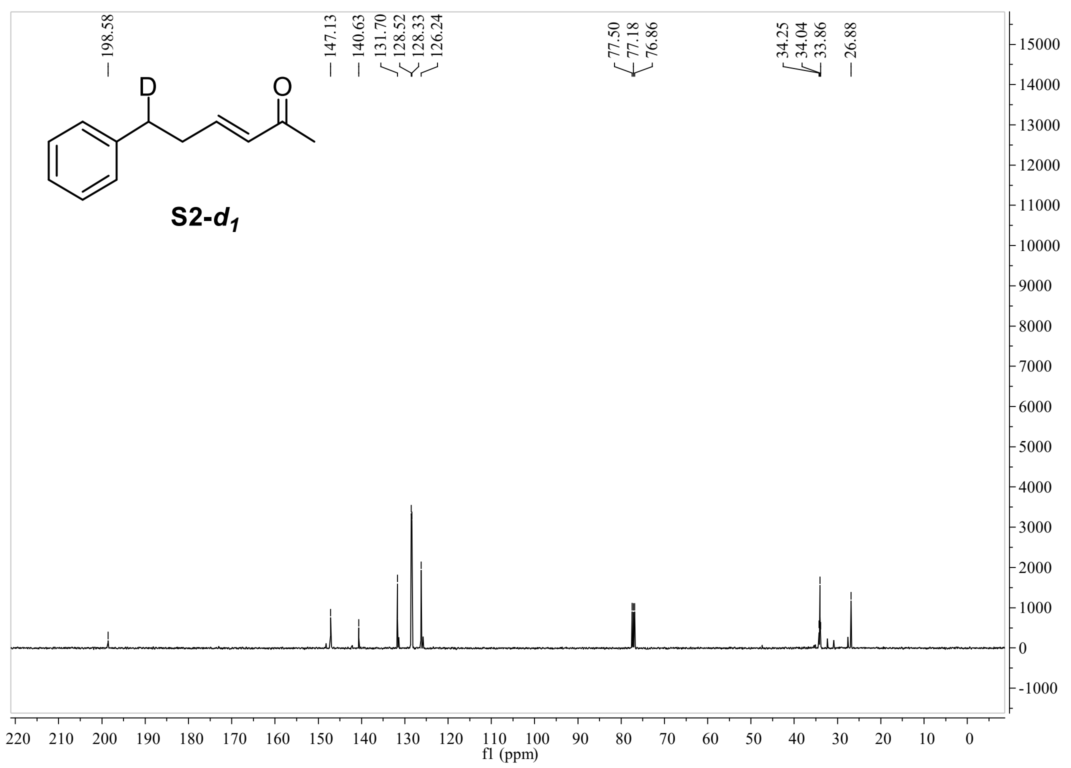

Figure S17 ^{13}C NMR spectrum of compound **S2-*d*$_1$**, related to **Scheme 4**

KIE experiment: We use deuterated **S2-*d*$_1$** as starting materials to compare the initial reaction rates.

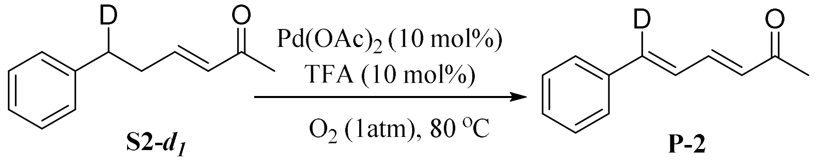

Figure S18 KIE experiment, related to **Scheme 4**

[6-*d*]-(3*E*,5*E*)-6-phenylhexa-3,5-dien-2-one. A seal tube containing **S2-*d*$_1$** (100%-D, 0.5 mmol) and Pd(OAc)$_2$ (10 mol%), was evacuated and filled with dioxygen gas using an oxygen containing balloon. Then, Dry DMSO (5 mL), trifluoroacetic acid (TFA) (1.0 mmol) were sequentially added to the system via syringe under an oxygen atmosphere. The reaction mixture was stirred at 80 °C until completion of the reaction (TLC). Then the reaction was cooled to RT and partitioned between water and ethyl acetate. The layers were separated and the organic layer was washed with aqueous saturated brine solution, dried over Na_2SO_4, filtered and concentrated under reduced pressure. ^{1}H NMR specture was recorded without Further purification. (90% yield, 56% D). **k_H / k_D = 1.2 was also obtained by calculation of the ratio of two products.**

The ^{1}H NMR specture as follows:

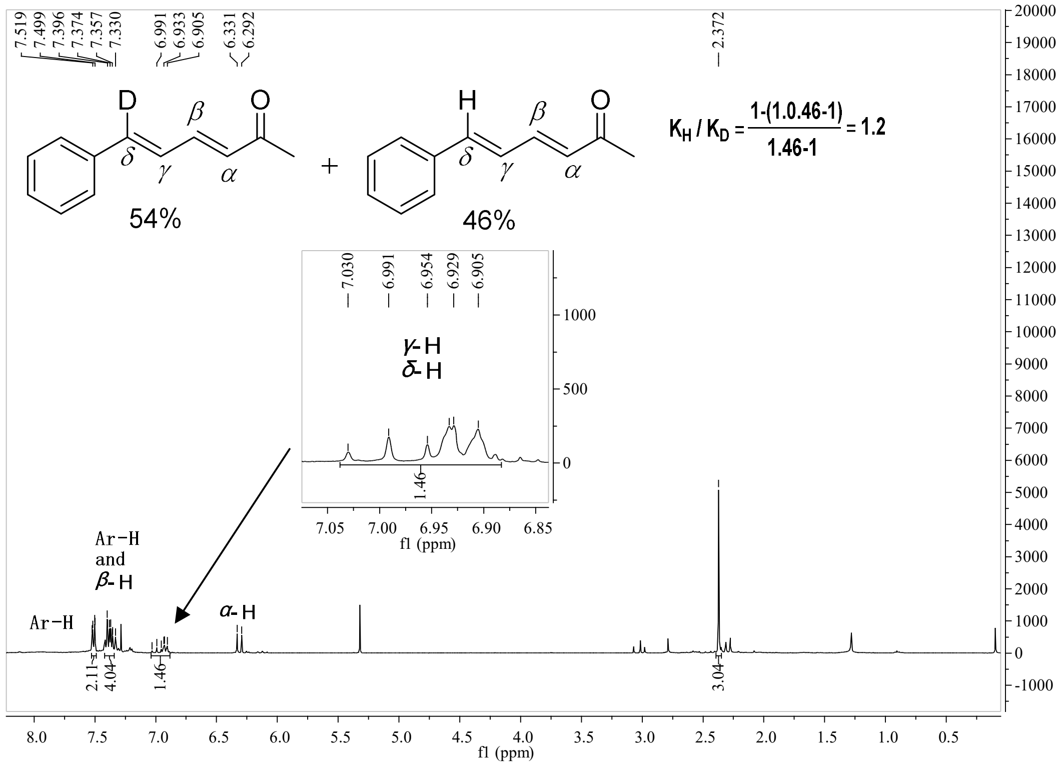

Figure S19 ^{1}H NMR spectrum for the KIE value, related to **Scheme 4**

4. Supplemental references

Ma, D., Yu, Y. and Lu, X. (1989). Highly stereoselective isomerization of ynones to conjugated dienones catalyzed by transition-metal complexes. J. Org. Chem. *54*, 1105–1109.

Kim, H. Y. and Oh, K. (2015). 1,3-Dienones and 2H-Pyran-2-ones from Soft *α*-Vinyl Enolization of *β*-Chlorovinyl Ketones: Defined Roles of Brönsted and Lewis Base. Org. Lett. *17*, 6254–6257.

Armstrong, A., Pullin, R. D. C., Jenner, C. R. and Scutt, J. N. (2010). Amine-Promoted Synthesis of Vinyl Aziridines. J. Org. Chem. **75**, 3499–3502.

Yoo, K. S., Yoon, C. H. and Jung, K. W. (2006). Oxidative Palladium(II) Catalysis: A Highly Efficient and Chemoselective Cross-Coupling Method for Carbon−Carbon Bond Formation under Base-Free and Nitrogenous-Ligand Conditions. J. Am. Chem. Soc. *128*, 16384–16393.

Zou, Y., Garayalde, D., Wang, Q., Nevado, C. and Goeke, A. (2008). Gold-Catalyzed Cycloisomerization of Cyclopropyl Alkynyl Acetates: A Versatile Approach to 5-, 6-, and 7-Membered Carbocycles. Angew. Chem. Int. Ed. *47*, 10110–10113.

Wu, X., Xie, F., Ling Z., Tang, L. and Zhang, W. (2016). Regio- and Enantioselective Copper-Catalyzed 1,4-Conjugate Addition of Trimethylaluminium to Linear *α,β,γ,δ*-Unsaturated Alkyl Ketones. Adv. Synth. Catal. *358*, 2510–2518.

Polaquini, C. R., Torrezan, G. S.; Santos, V. R., Nazaré, A. C., Campos, D. L.,

Almeida, L. A., Silva, I. C., Ferreira, H., Pavan, F. R., Duque, C. and Regasini, L. O. (2017). Antibacterial and Antitubercular Activities of Cinnamylideneacetophenones. Molecules *22*, 1685–1696.

Kelly, C. B., Ovian, J. M., Cywar, R. M., Gosselin, T. R., Wiles, R. J. and Leadbeater, N. E. (2015). Oxidative cleavage of allyl ethers by an oxoammonium salt. Org. Biomol. Chem. 13, 4255–4259.

Holan, M. and Jahn, U. (2014). Anaerobic Nitroxide-Catalyzed Oxidation of Alcohols Using the NO+/NO· Redox Pair. Org. Lett. *16*, 58–61.

Riveira, M. J. and Mischne, M. P. (2012). One-Pot Organocatalytic Tandem Aldol/Polycyclization Reactions between 1,3-Dicarbonyl Compounds and *α,β,γ,δ*-Unsaturated Aldehydes for the Straightforward Assembly of Cyclopenta[b]furan-Type Derivatives: New Insight into the Knoevenagel Reaction. Chem.-Eur. J. *18*, 2382–2388.

Chandrasekhar, S., Venkat Reddy, M. K. Reddy, S. and Ramarao, C. (2000). Tetrahedron Lett. 41, 2667–2670.

Ariza, X., Asins, G., Garcia, J.; Hegardt, F. G. Makowski, K., Serra, D. and Velasco, J. (2010). J. Label Compd. Radiopharm. *53*, 556–558.

Shih, J. L., Nguyen, T. S. and May, J. A. (2015). Organocatalyzed Asymmetric Conjugate Addition of Heteroaryl and Aryl Trifluoroborates: a Synthetic Strategy for Discoipyrrole D. Angew. Chem. Int. Ed, *54*, 9931–9935

Diao, T., Wadzinski, T. J. and Stahl, S. S. (2012). Direct aerobic α,β-dehydrogenation of aldehydes and ketones with a $Pd(TFA)_2$ / 4,5-diazafluorenone catalyst. Chem. Sci. *3*, 887–891.

Peart, P. C., McCook, K. P., Russell, F. A., Reynolds, W. F. and Reese, P. B. (2011). Hydroxylation of steroids by Fusarium oxysporum, Exophiala jeanselmei and Ceratocystis paradoxa. *Steroids 76*, 1317–1330.

5. Supplemental Figures for ^{1}H and ^{13}C NMR spectra

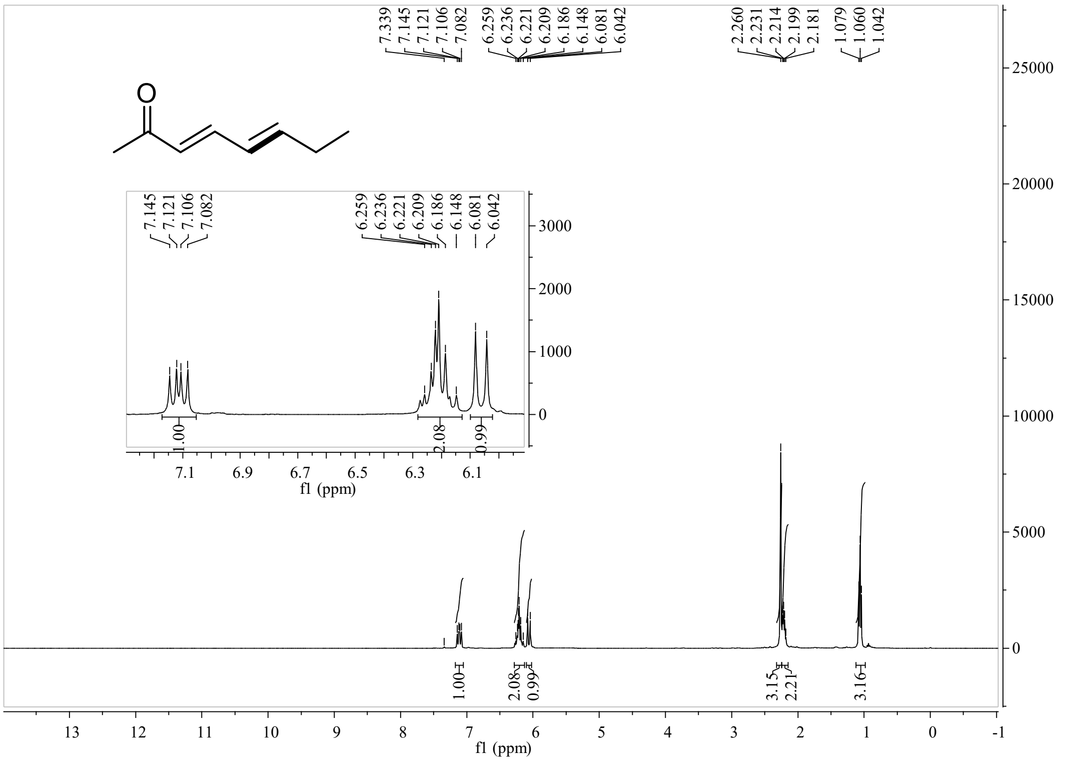

Figure S20 ^{1}H NMR spectrum of compound **2aa**, related to **Scheme 2**

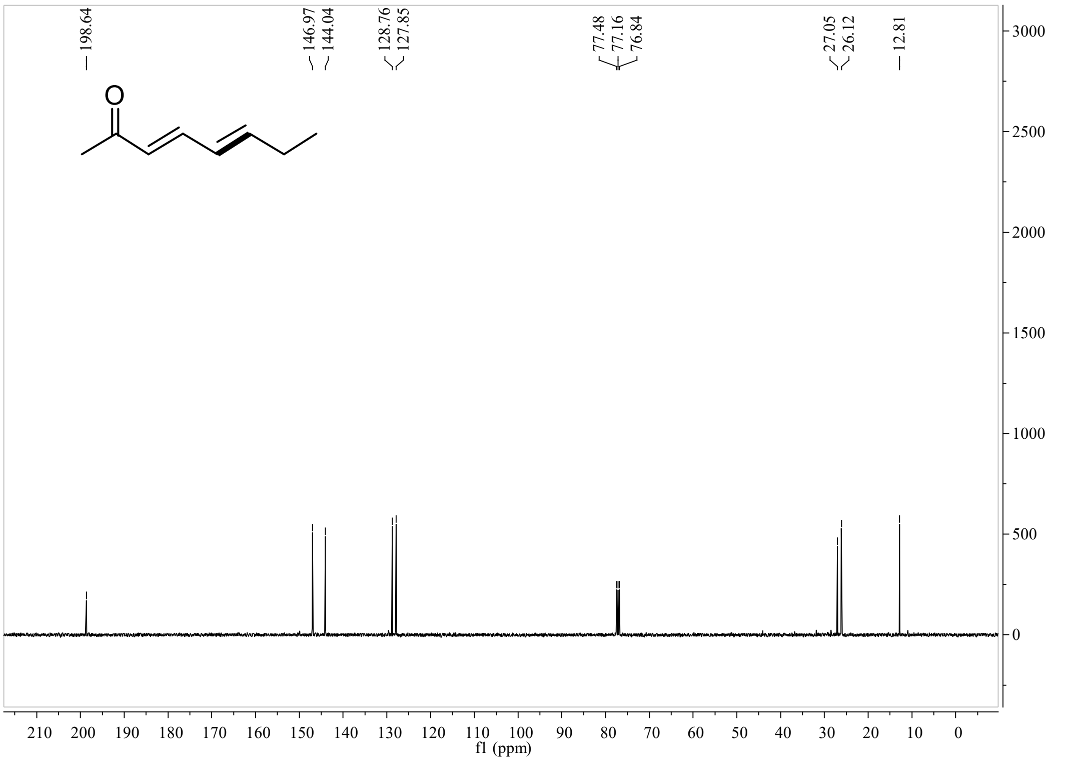

Figure S21 ^{13}C NMR spectrum of compound **2aa**, related to **Scheme 2**

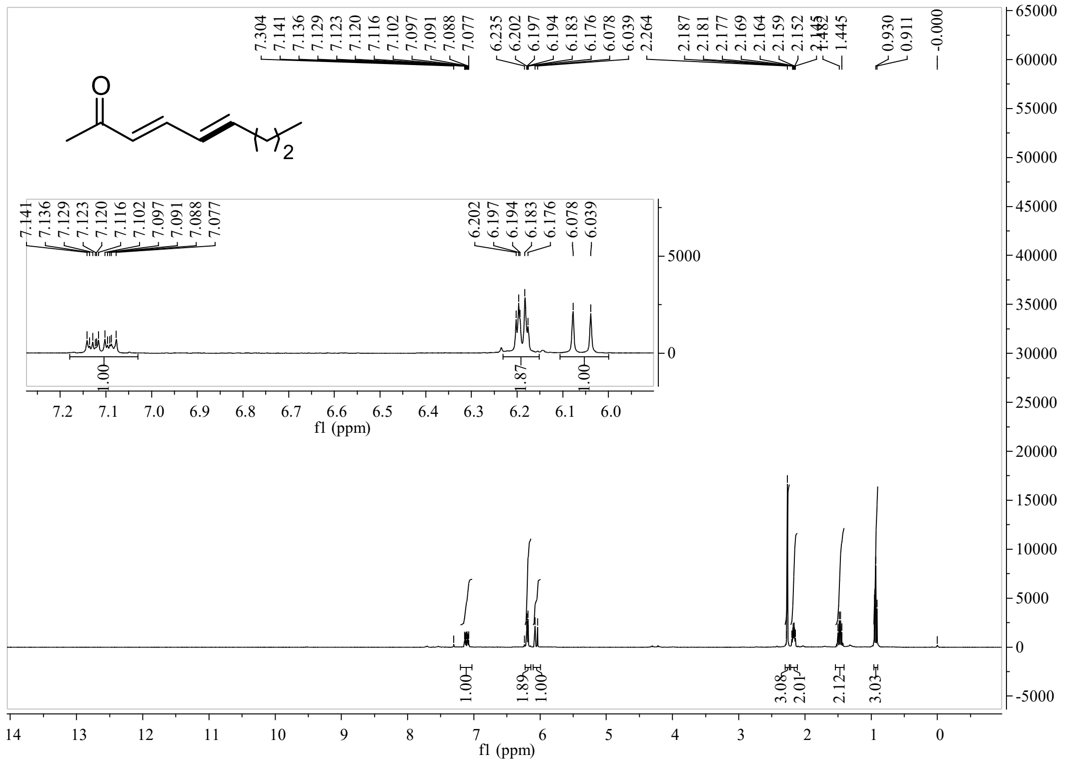

Figure S22 ^{1}H NMR spectrum of compound **2ab**, related to **Scheme 2**

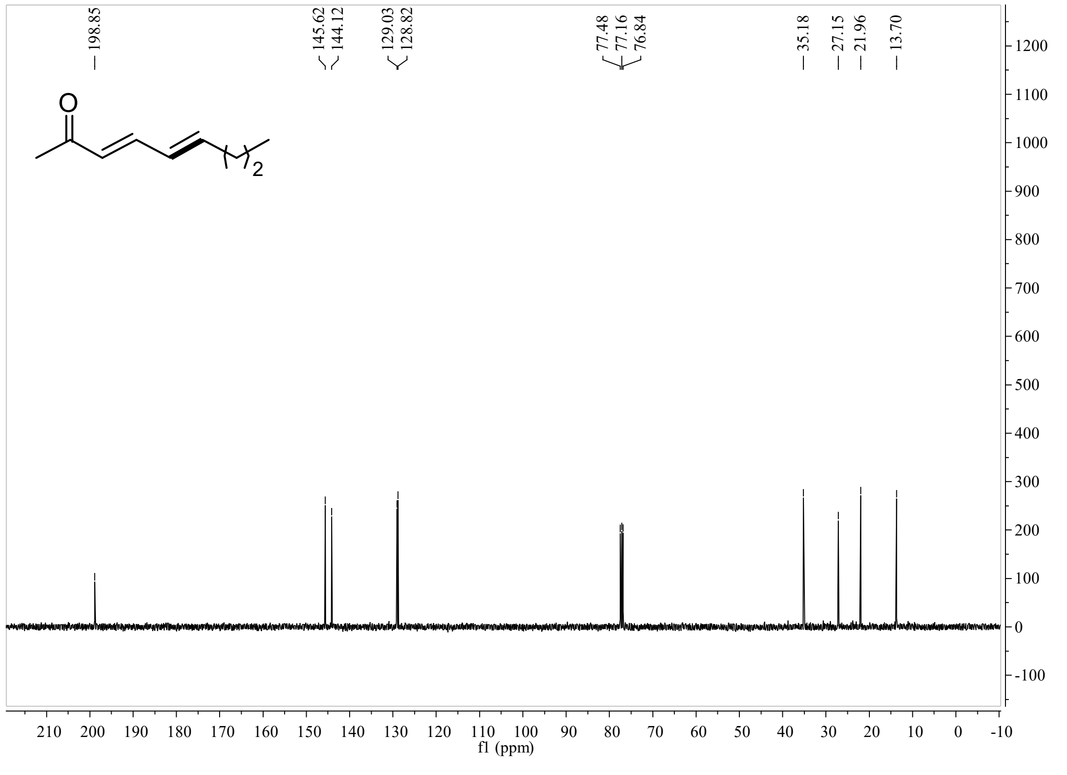

Figure S23 ^{13}C NMR spectrum of compound **2ab**, related to **Scheme 2**

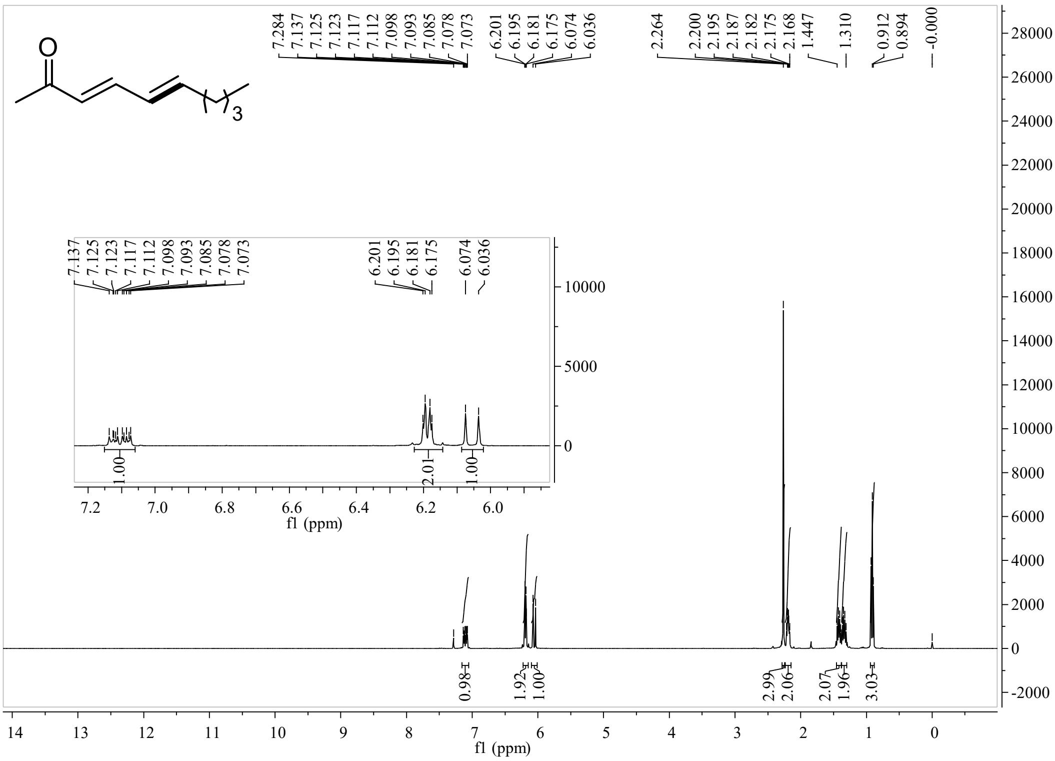

Figure S24 ^{1}H NMR spectrum of compound **2ac**, related to **Scheme 2**

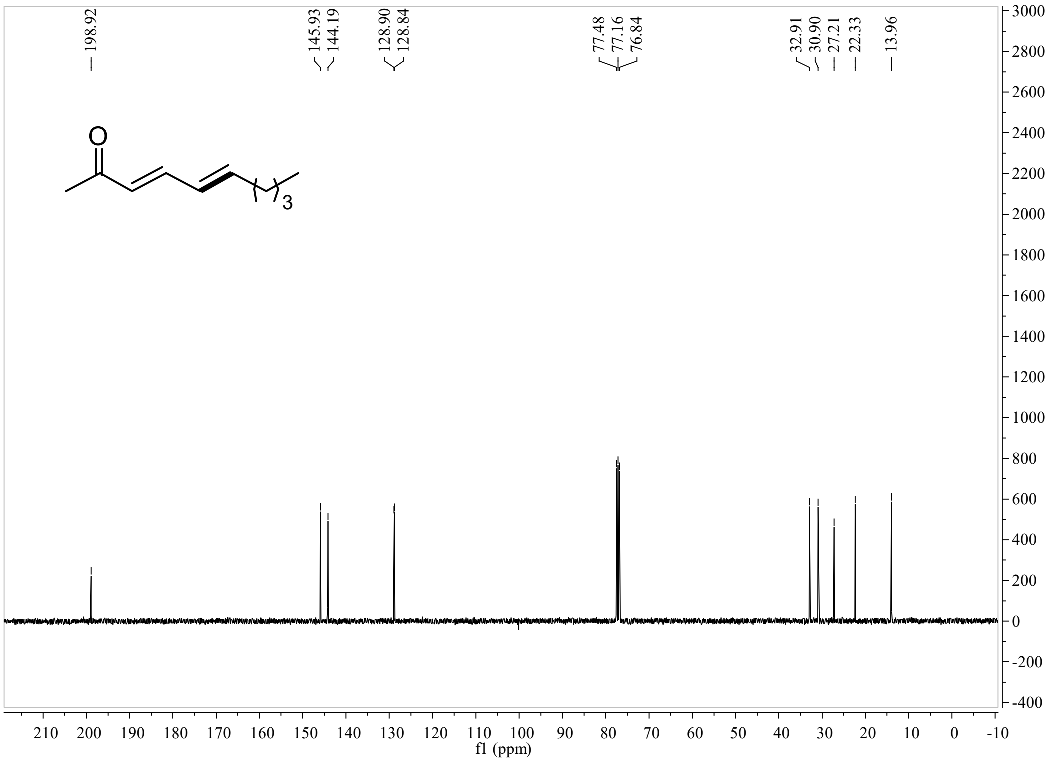

Figure S25 ^{13}C NMR spectrum of compound **2ac**, related to **Scheme 2**

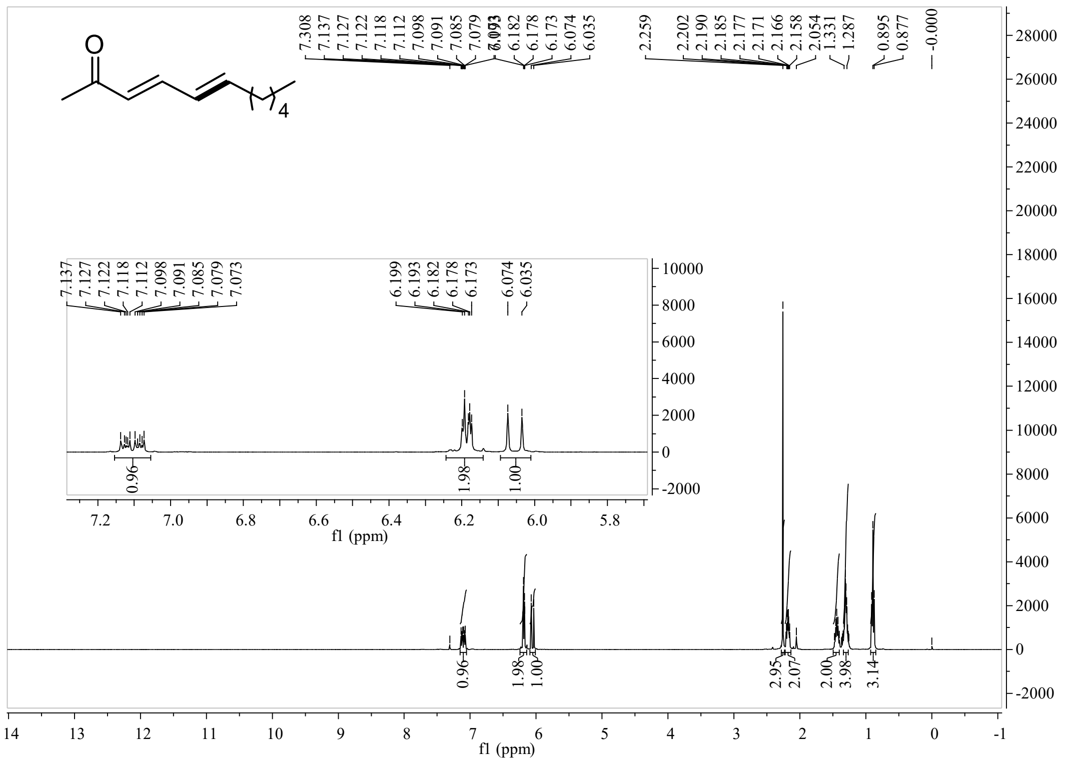

Figure S26 ^{1}H NMR spectrum of compound **2ad**, related to **Scheme 2**

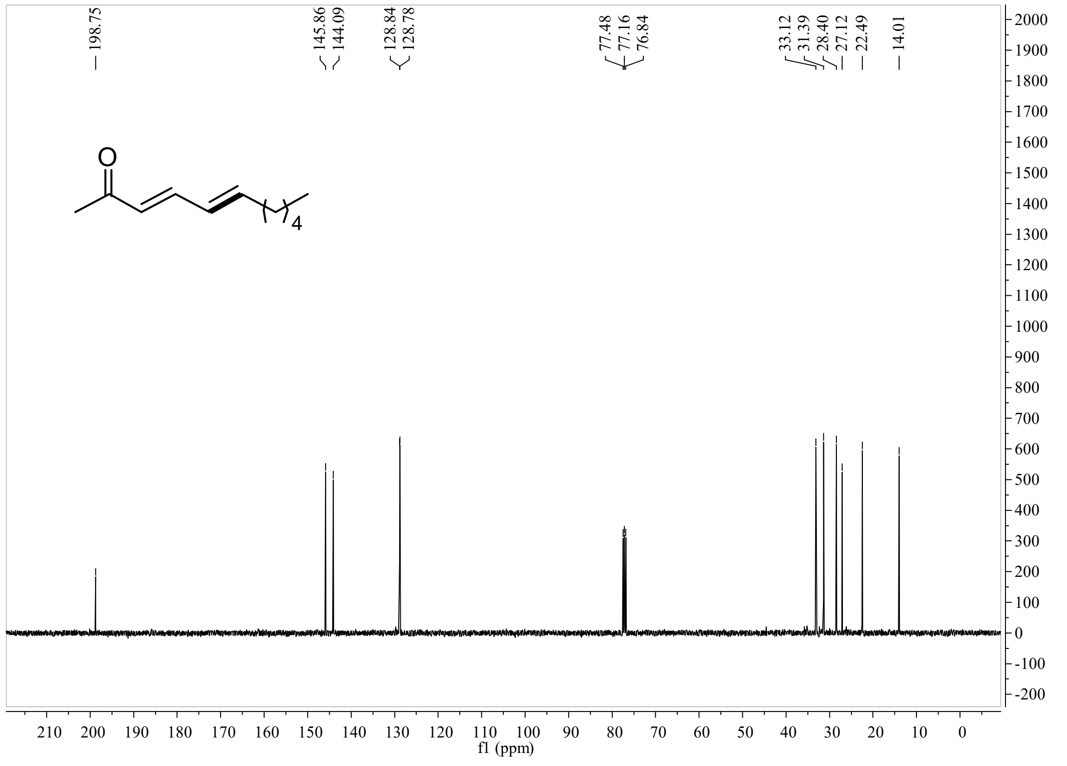

Figure S27 ^{13}C NMR spectrum of compound **2ad**, related to **Scheme 2**

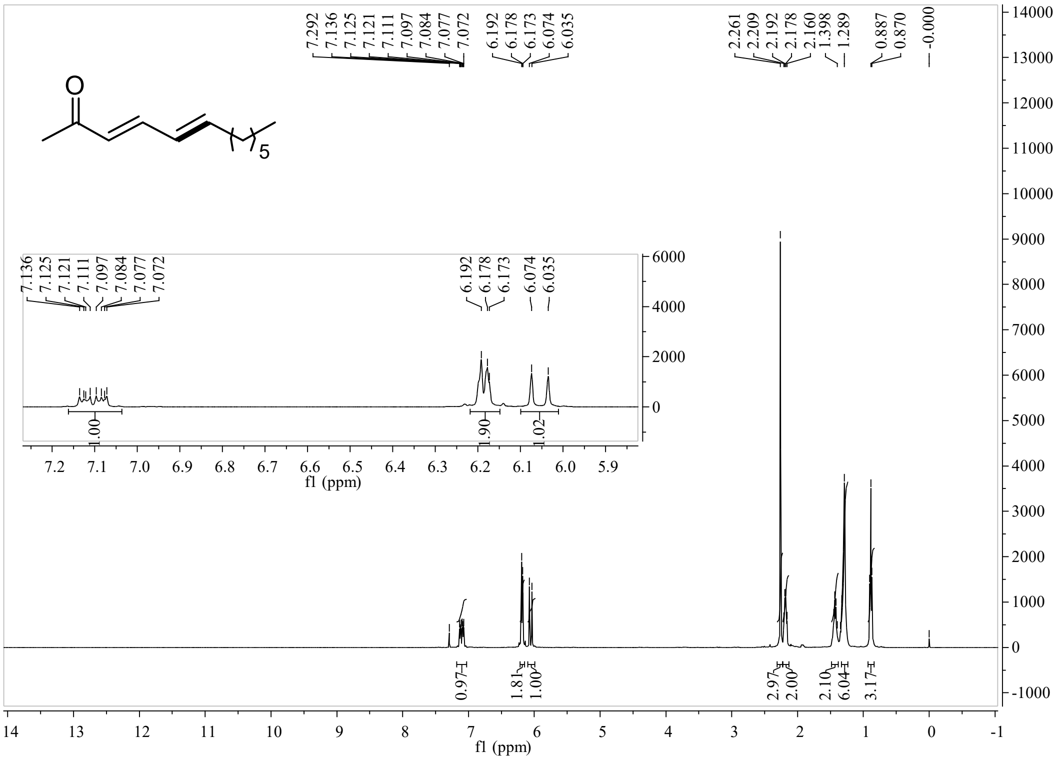

Figure S28 ^{1}H NMR spectrum of compound **2ae**, related to **Scheme 2**

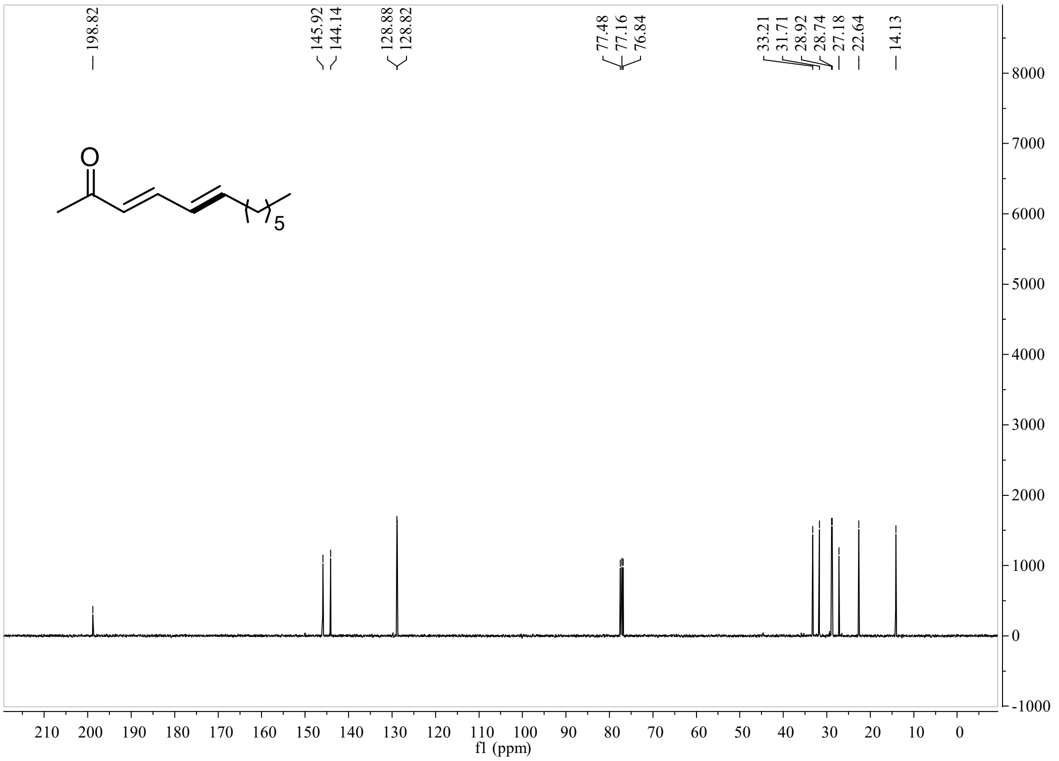

Figure S29 ^{13}C NMR spectrum of compound **2ae**, related to **Scheme 2**

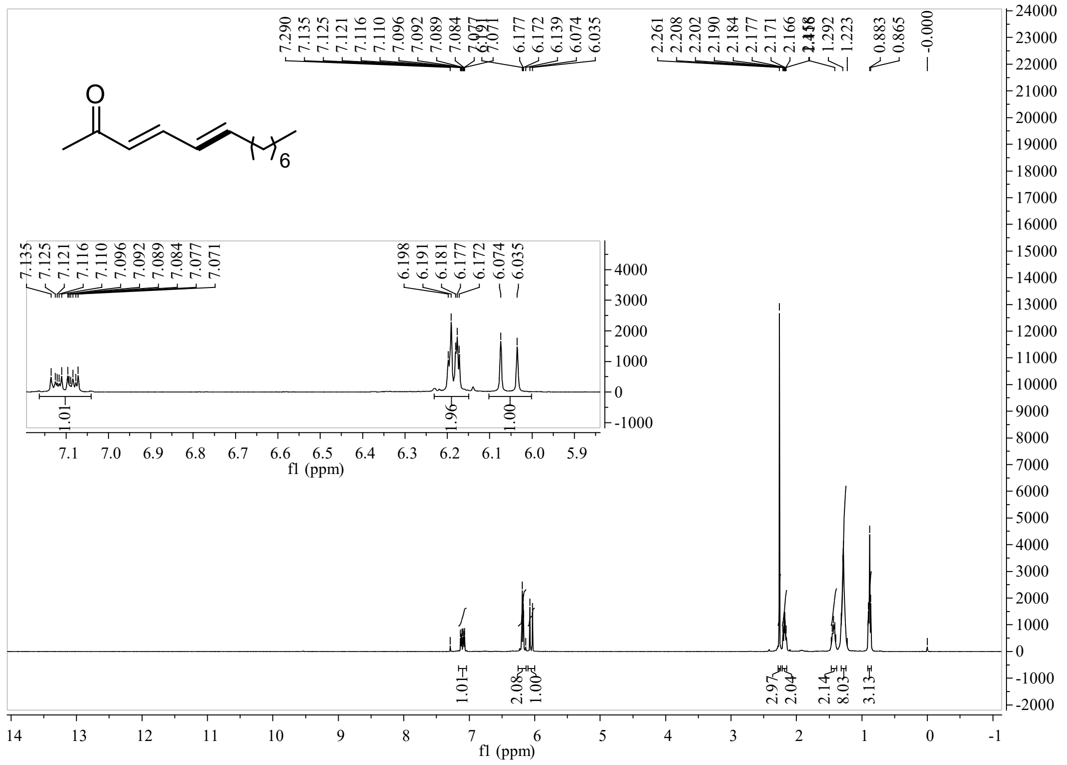

Figure S30 ^{1}H NMR spectrum of compound **2af**, related to **Scheme 2**

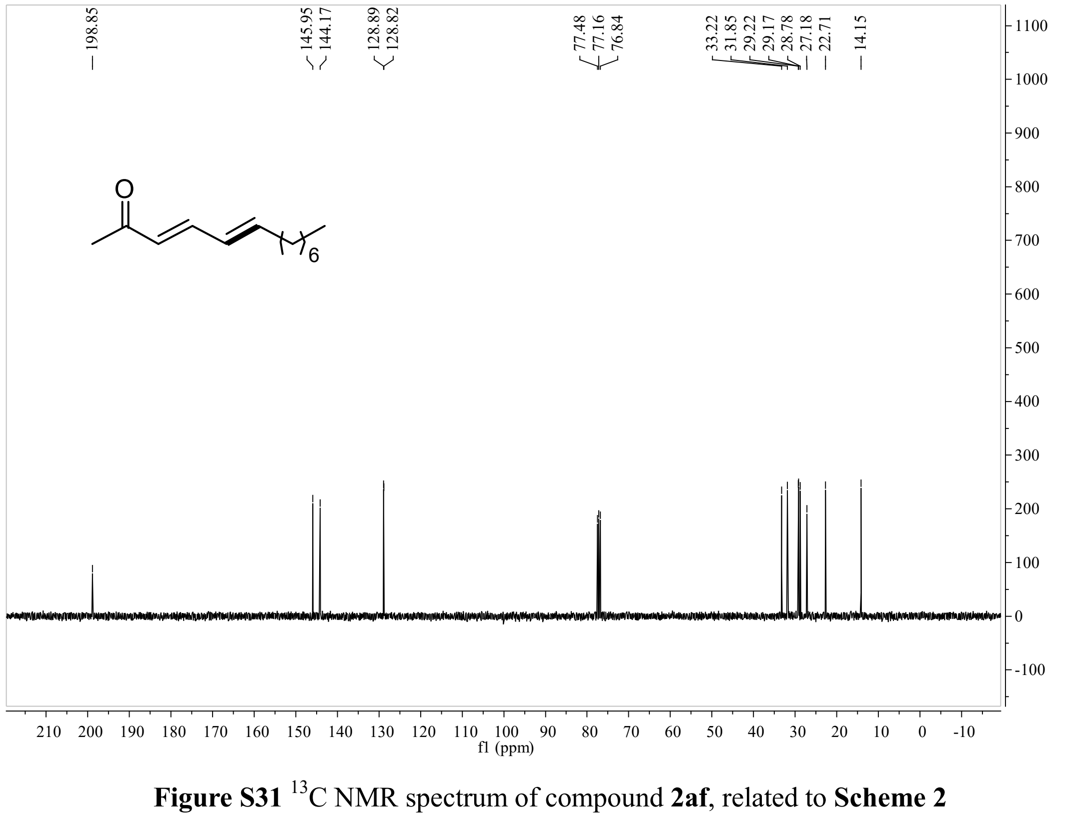

Figure S31 ^{13}C NMR spectrum of compound **2af**, related to **Scheme 2**

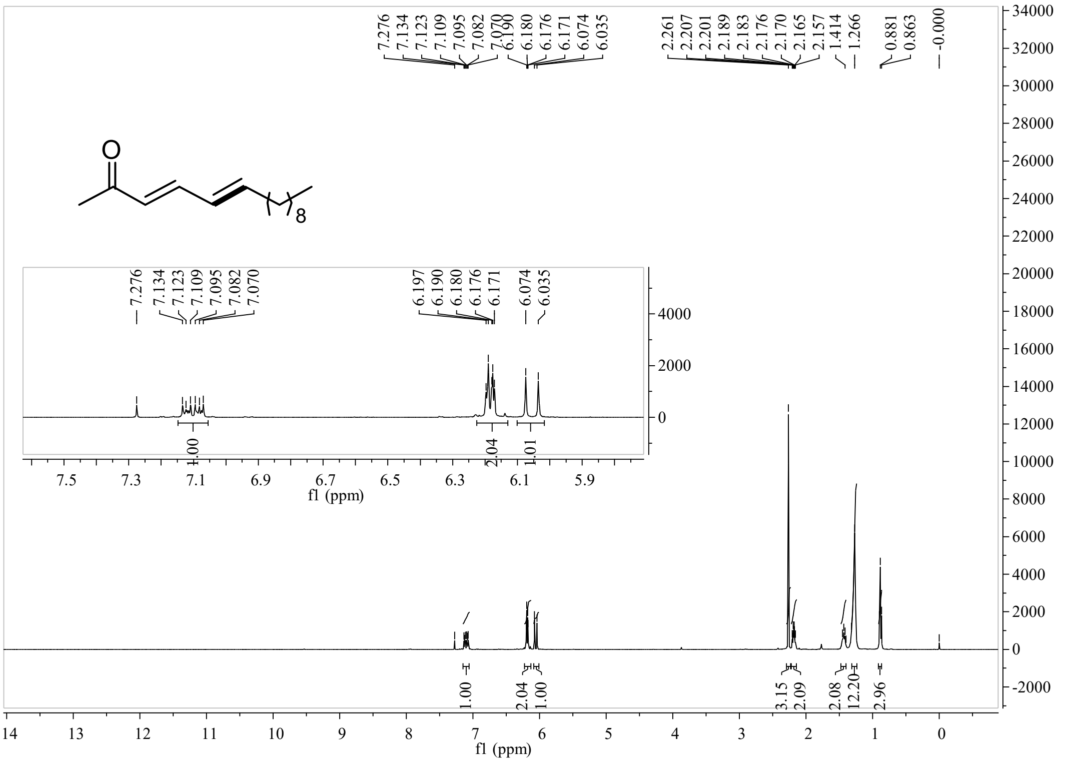

Figure S32 ^{1}H NMR spectrum of compound **2ag**, related to **Scheme 2**

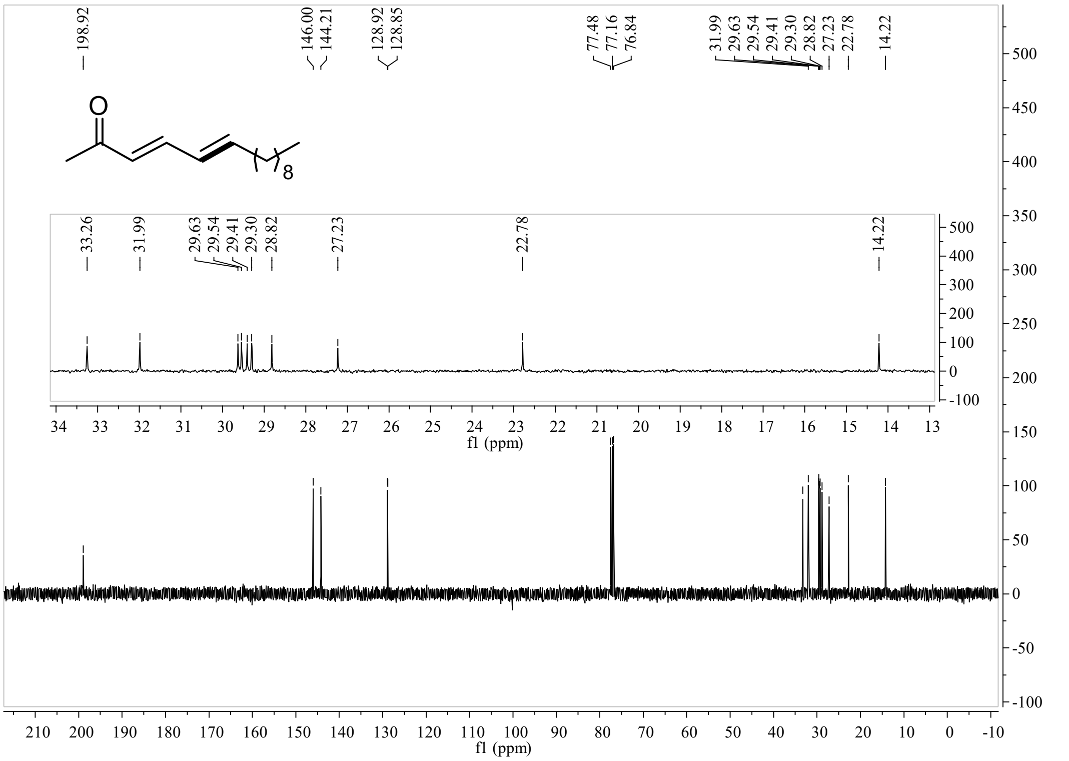

Figure S33 ^{13}C NMR spectrum of compound **2ag**, related to **Scheme 2**

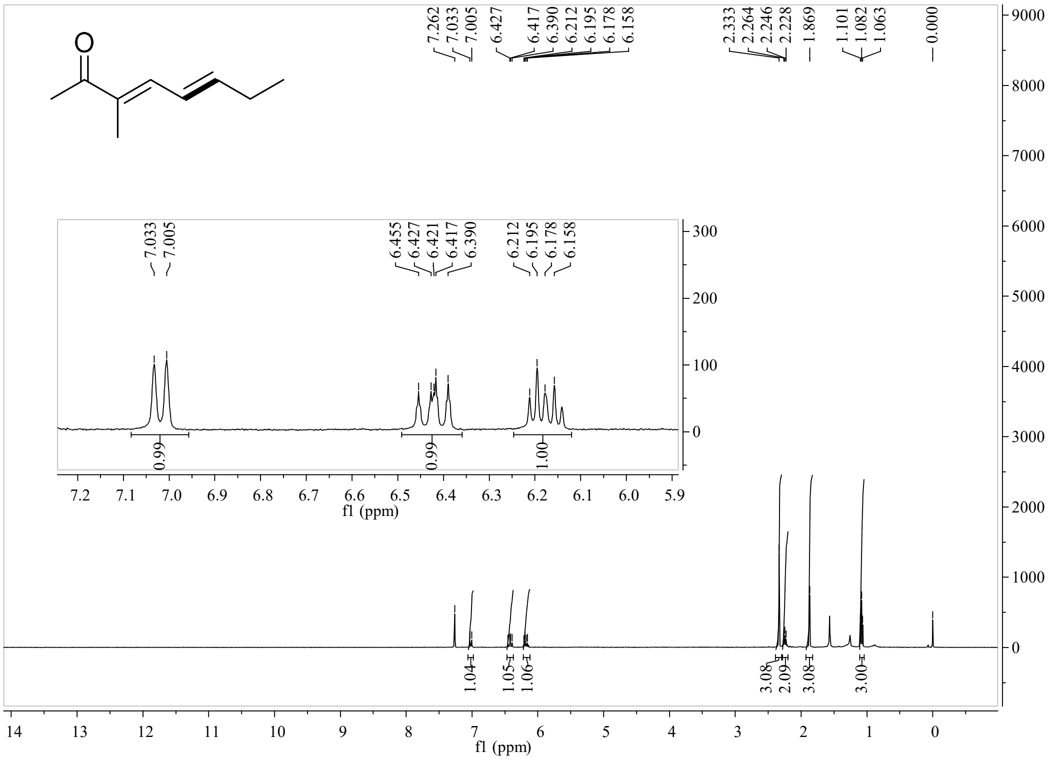

Figure S34 ^{1}H NMR spectrum of compound **2ah**, related to **Scheme 2**

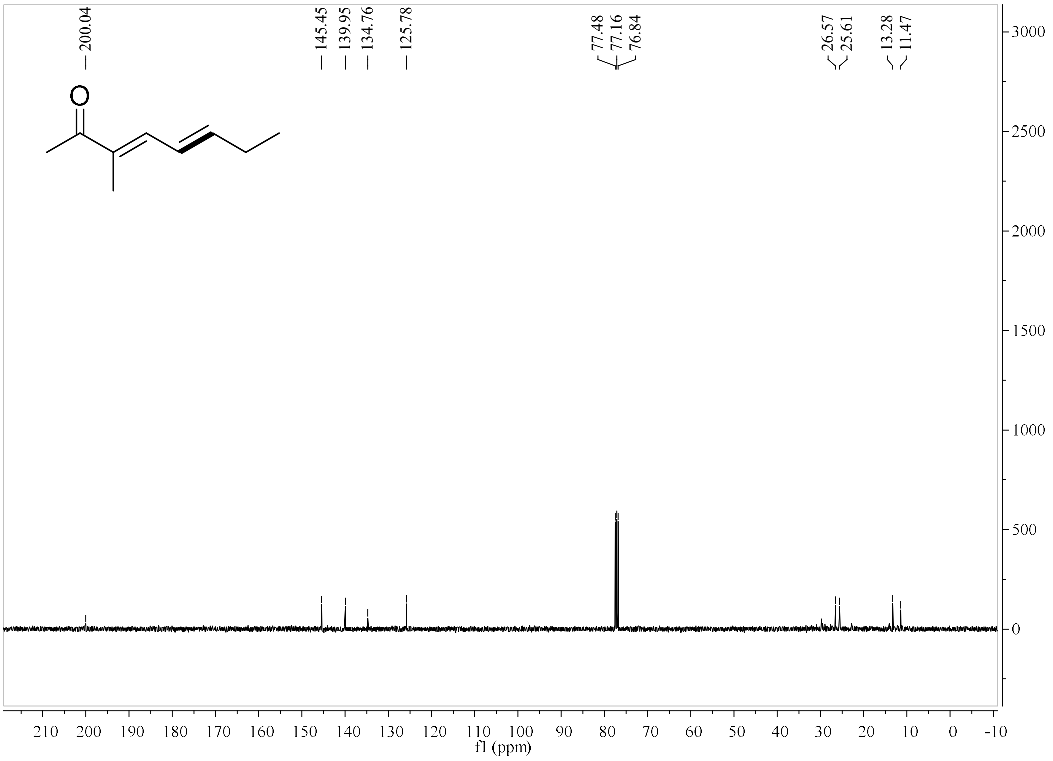

Figure S35 ^{13}C NMR spectrum of compound **2ah**, related to **Scheme 2**

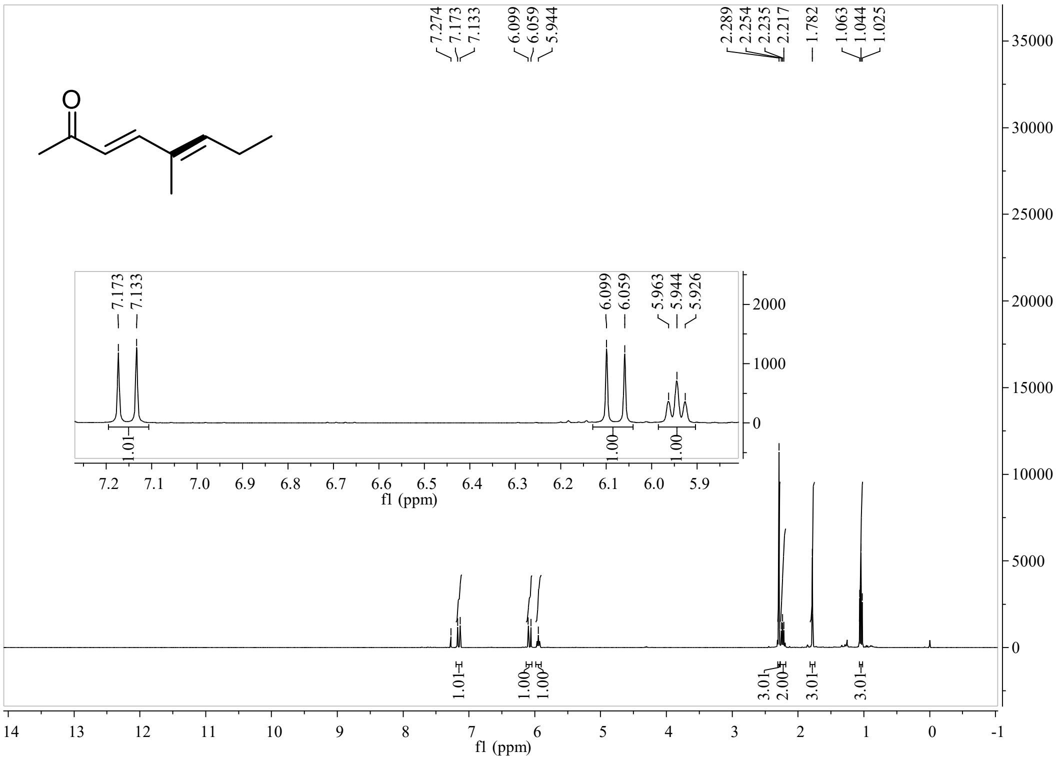

Figure S36 ^{1}H NMR spectrum of compound **2ai**, related to **Scheme 2**

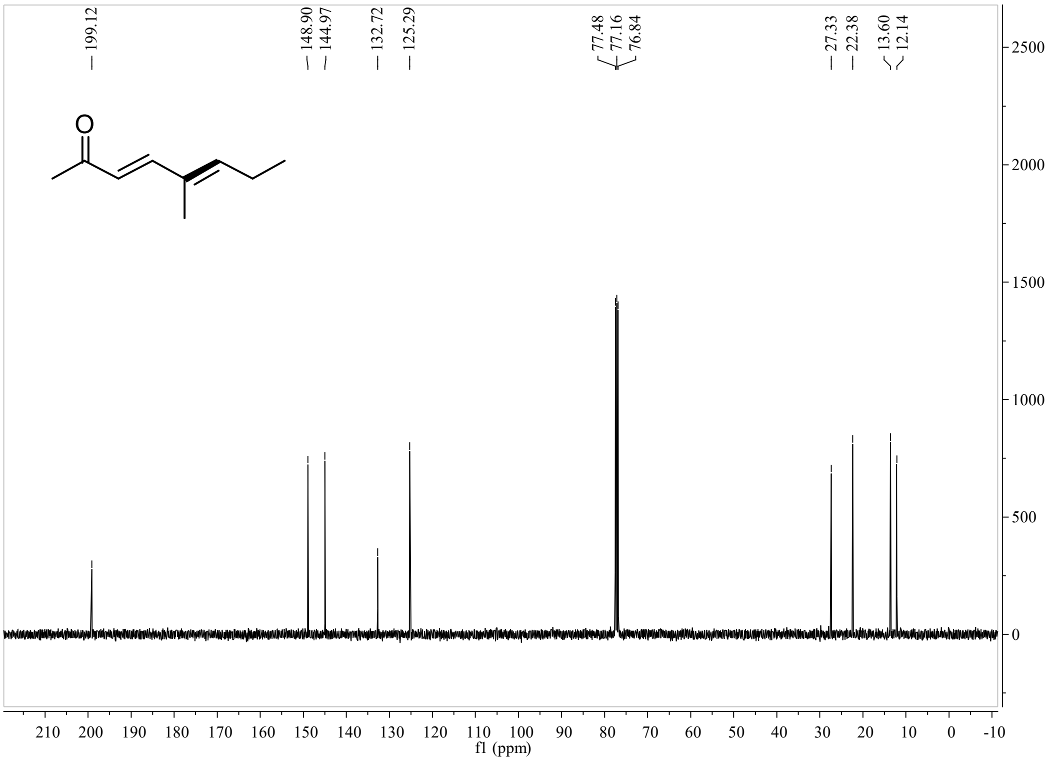

Figure S37 ^{13}C NMR spectrum of compound **2ai**, related to **Scheme 2**

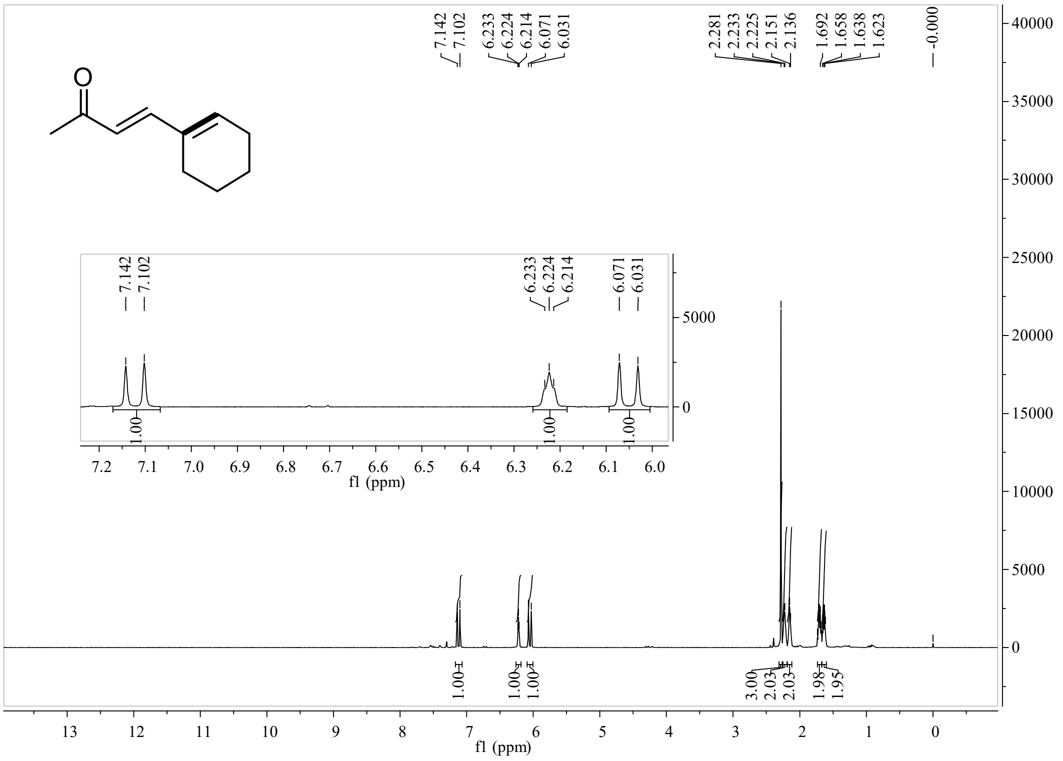

Figure S38 ^{1}H NMR spectrum of compound **2aj**, related to **Scheme 2**

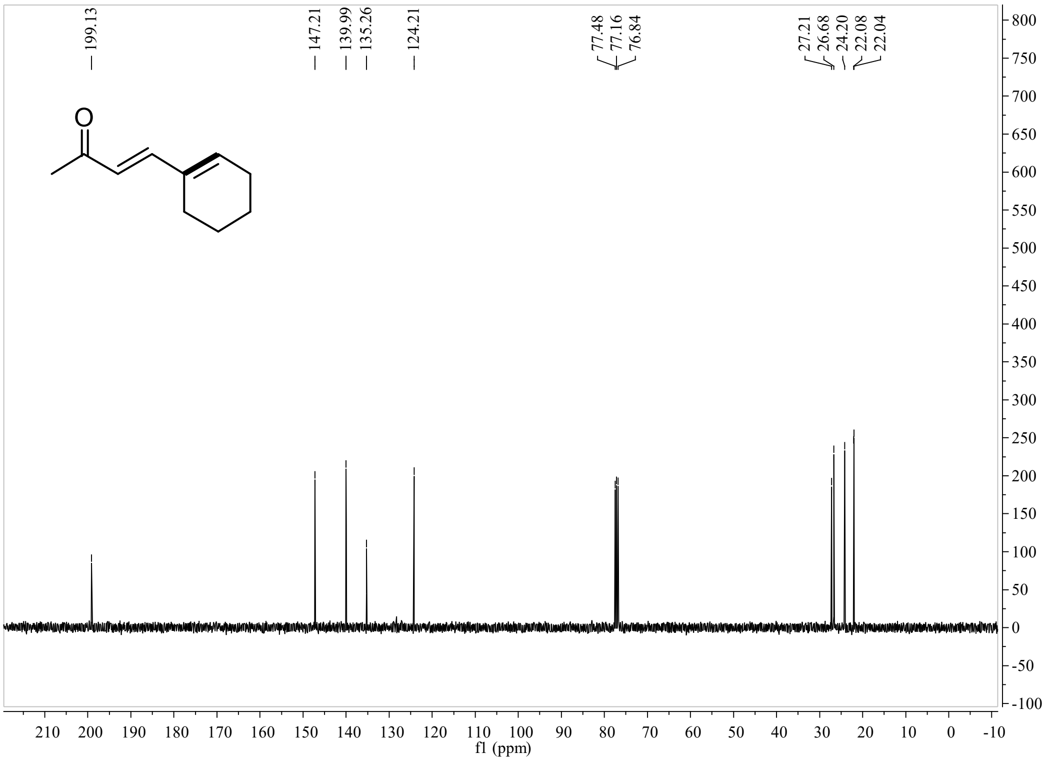

Figure S39 ^{13}C NMR spectrum of compound **2aj**, related to **Scheme 2**

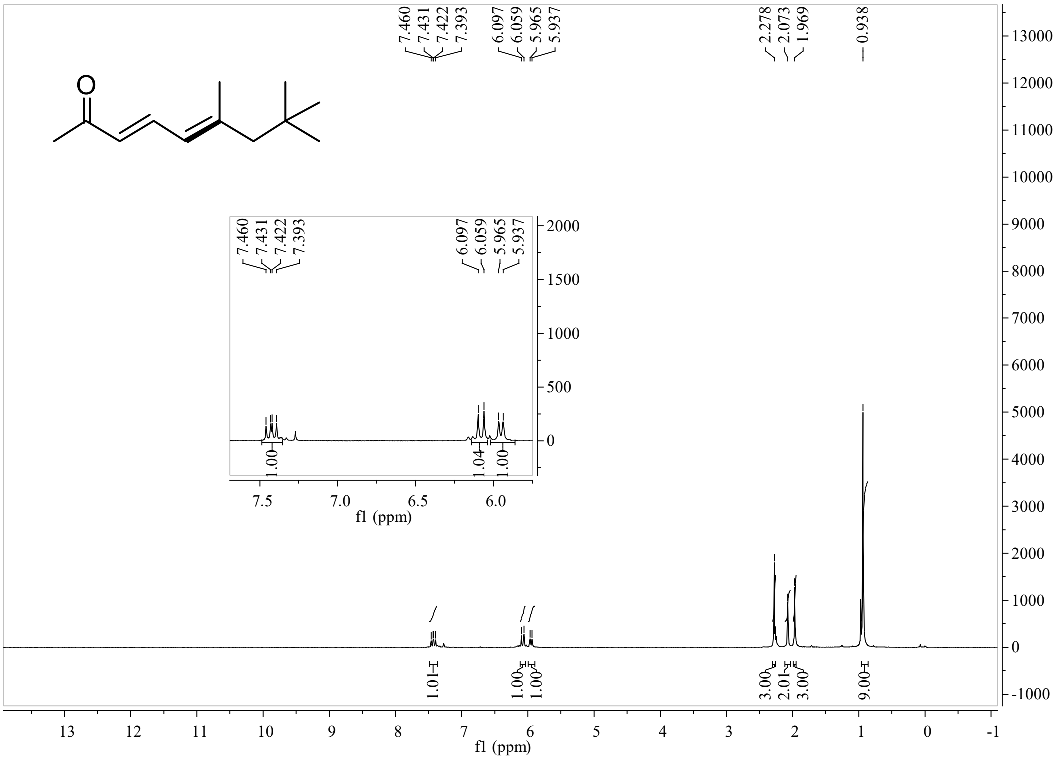

Figure S40 ^{1}H NMR spectrum of compound **2ak**, related to **Scheme 2**

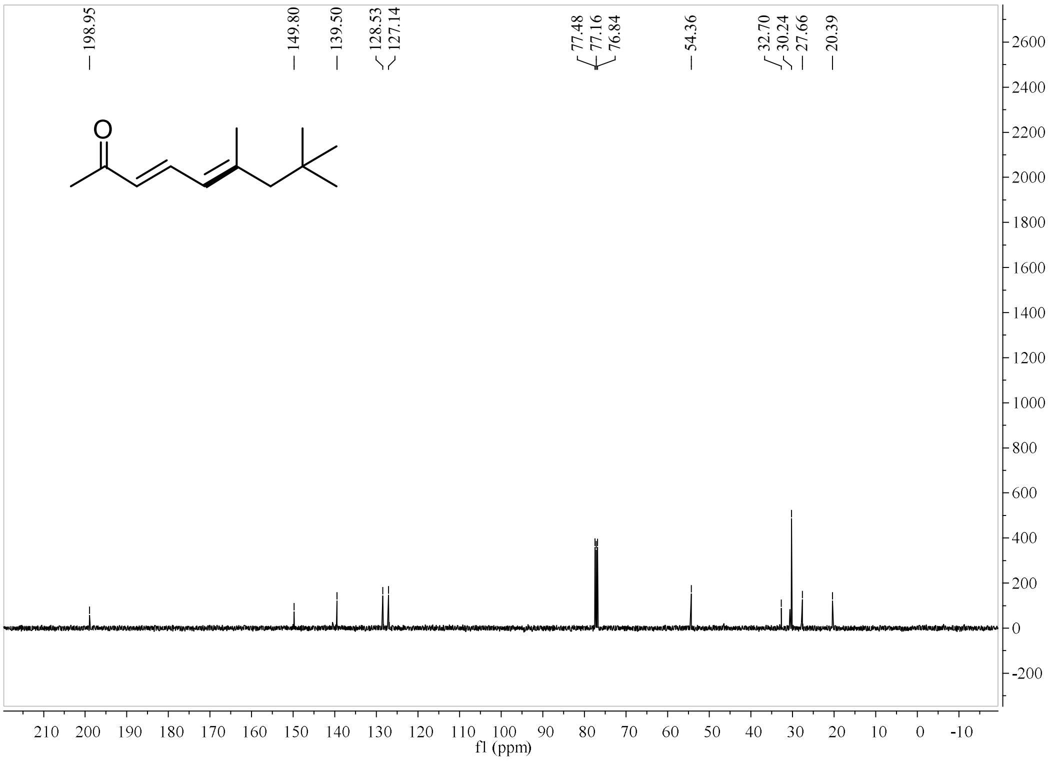

Figure S41 ^{13}C NMR spectrum of compound **2ak**, related to **Scheme 2**

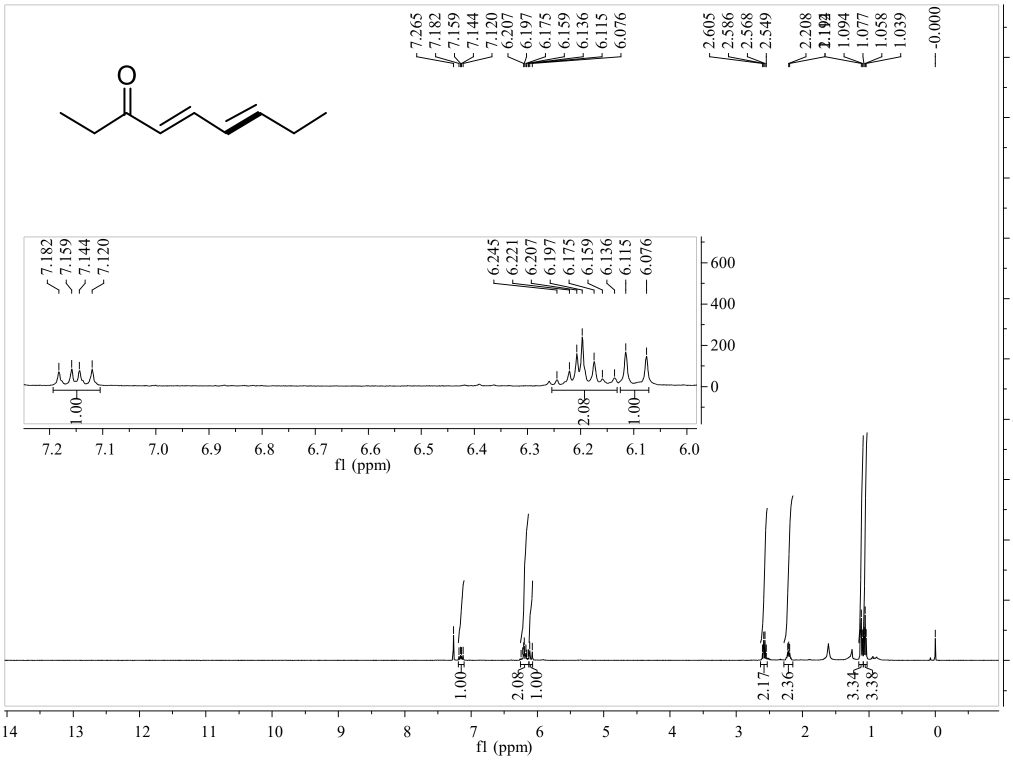

Figure S42 ^{1}H NMR spectrum of compound **2al**, related to **Scheme 2**

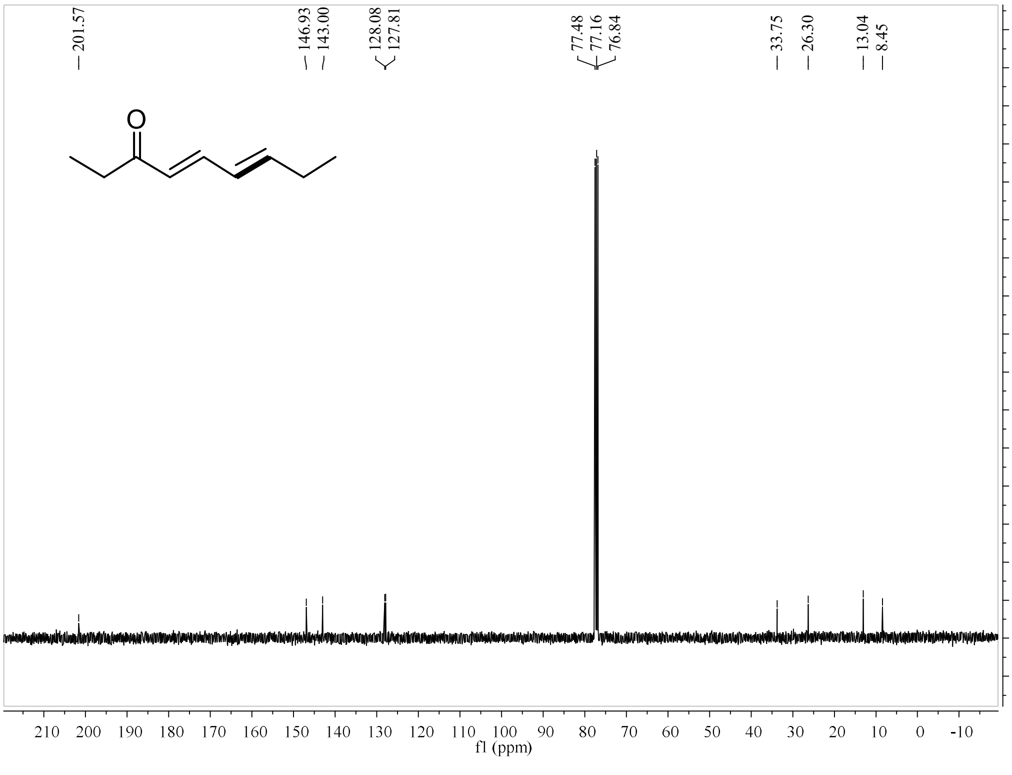

Figure S43 ^{13}C NMR spectrum of compound **2al**, related to **Scheme 2**

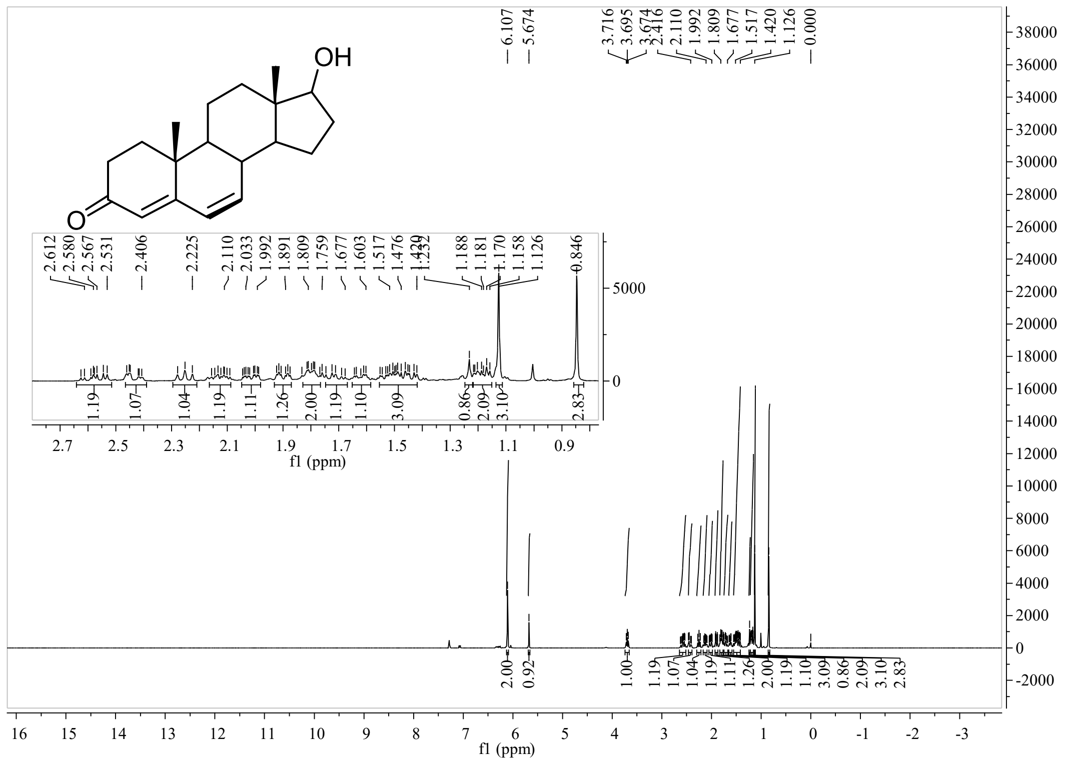

Figure S44 ^{1}H NMR spectrum of compound **2am**, related to **Scheme 2**

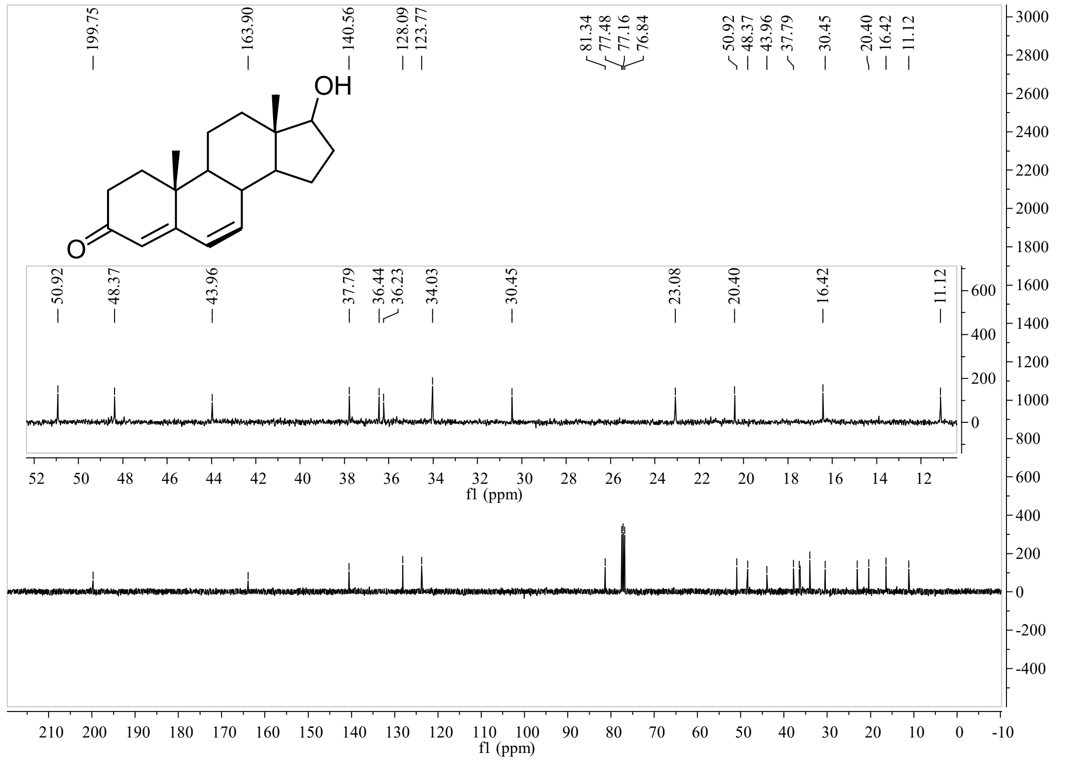

Figure S45 ^{13}C NMR spectrum of compound **2am**, related to **Scheme 2**

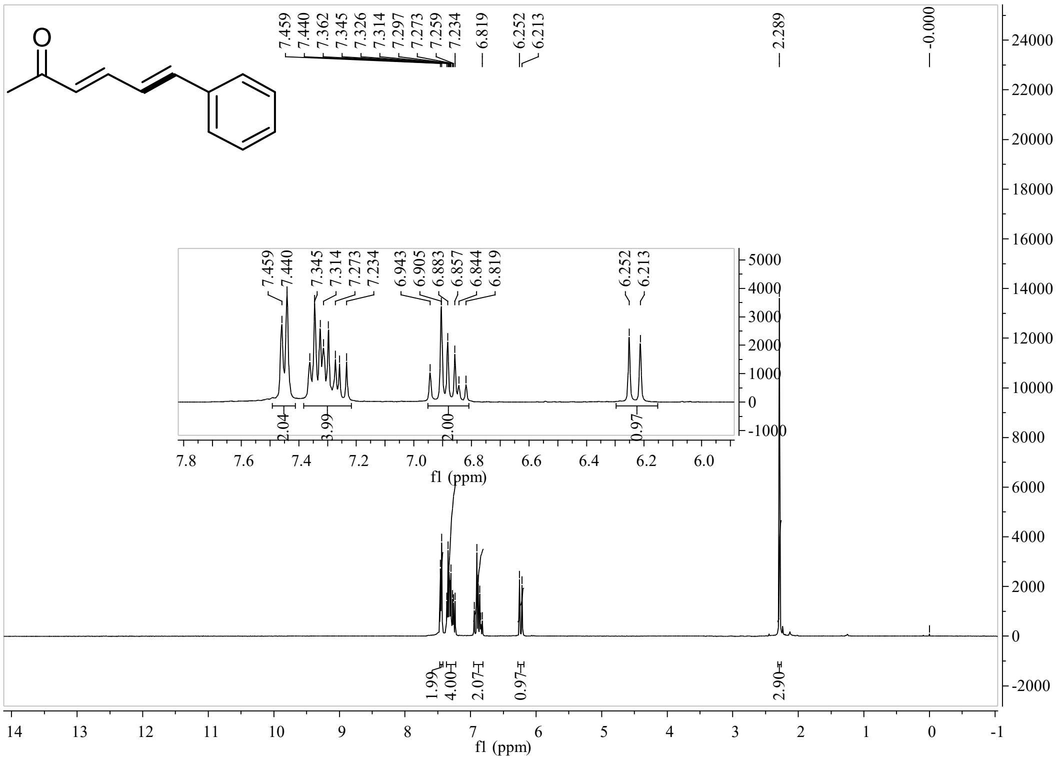

Figure S46 ^{1}H NMR spectrum of compound **2an**, related to **Scheme 2**

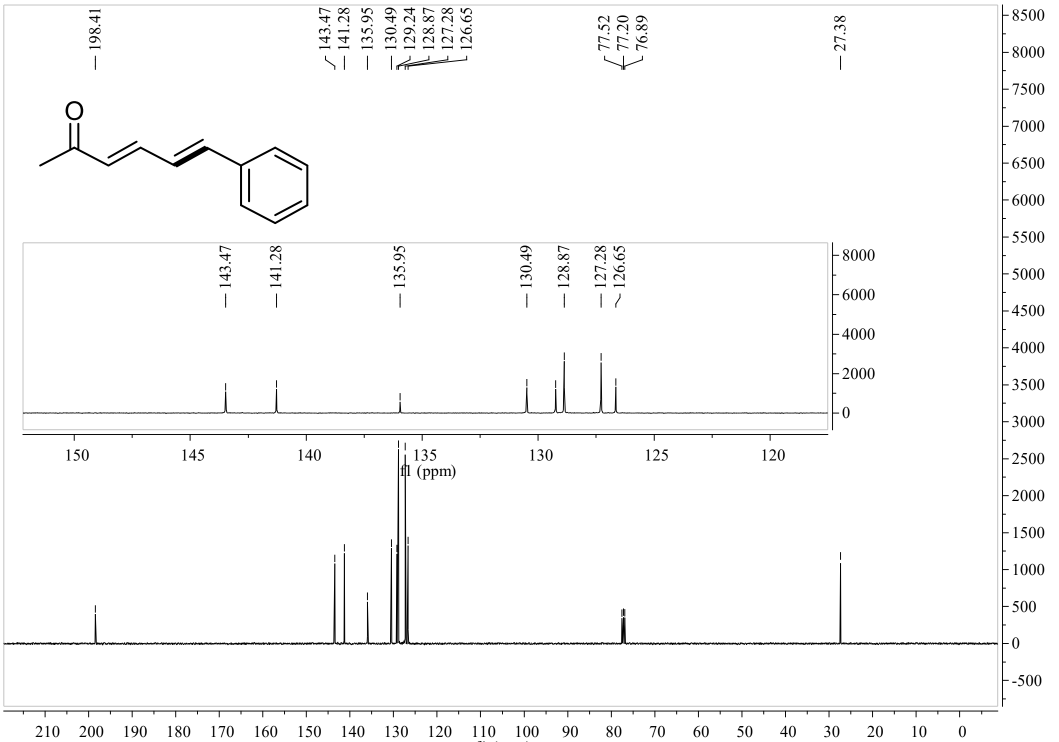

Figure S47 ^{13}C NMR spectrum of compound **2an**, related to **Scheme 2**

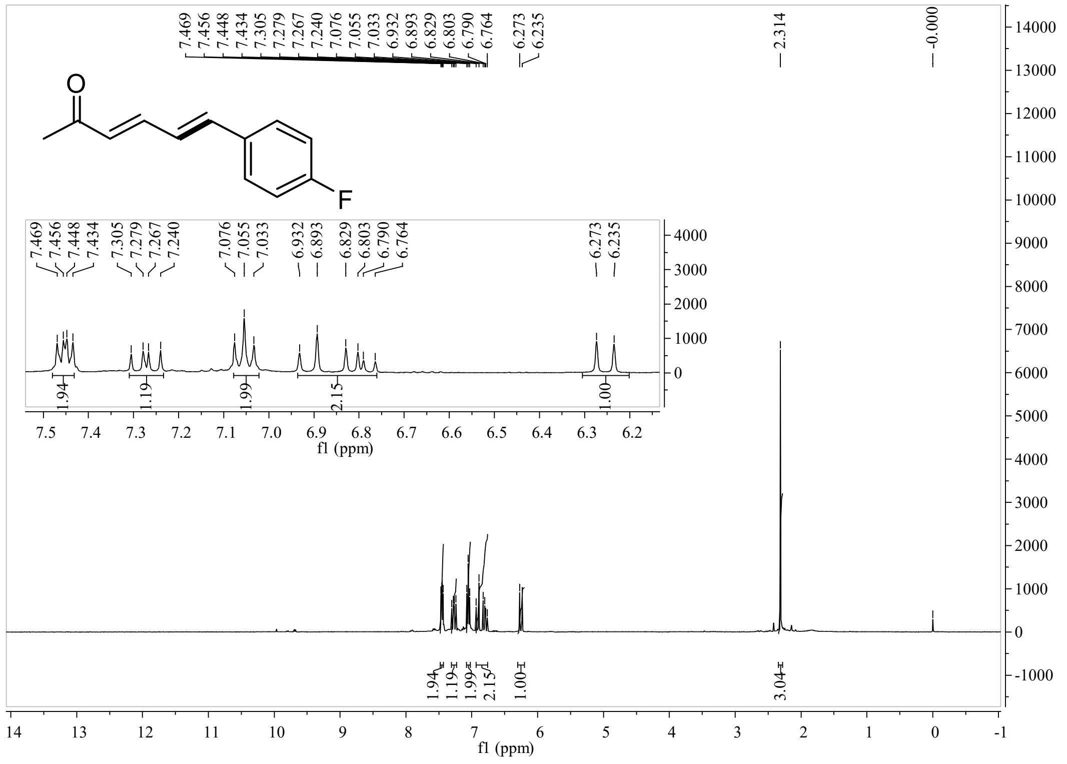

Figure S48 ^{1}H NMR spectrum of compound **2ao**, related to **Scheme 2**

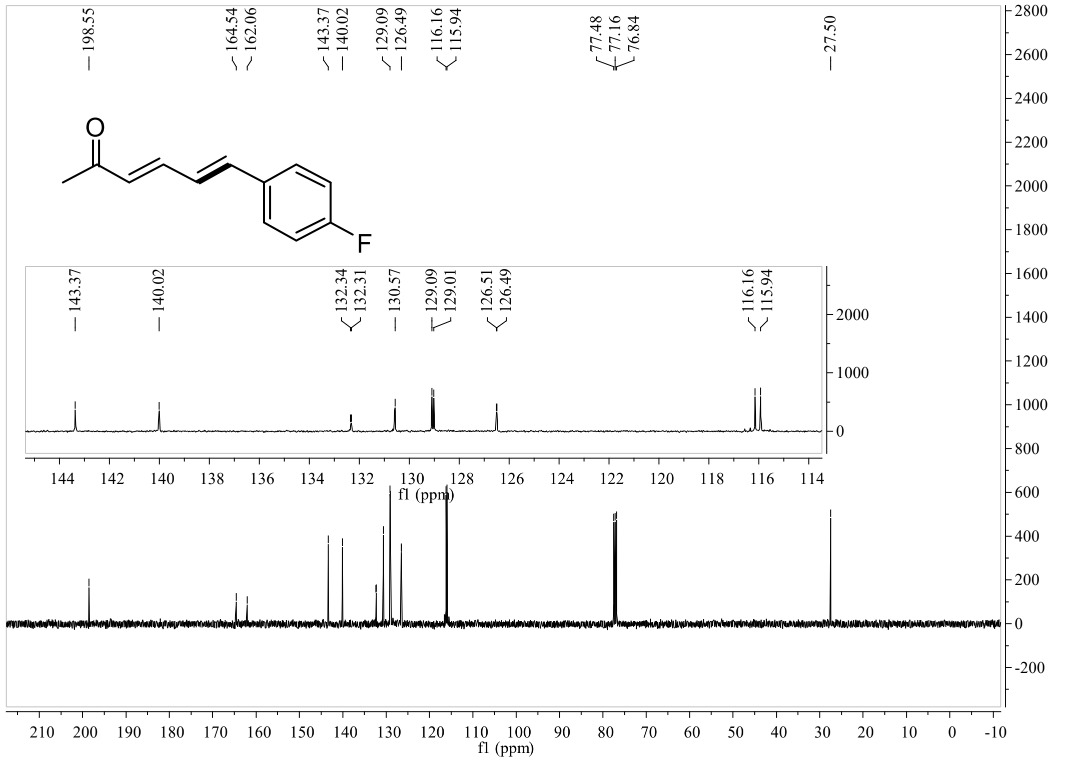

Figure S49 ^{13}C NMR spectrum of compound **2ao**, related to **Scheme 2**

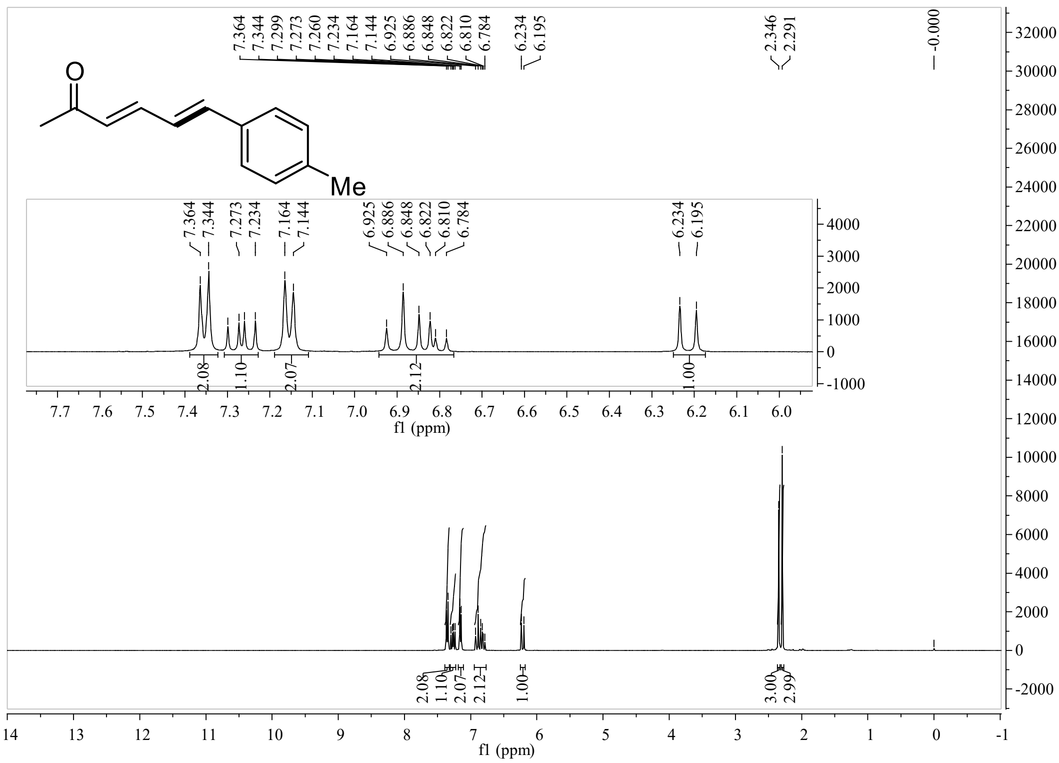

Figure S50 ^{1}H NMR spectrum of compound **2ap**, related to **Scheme 2**

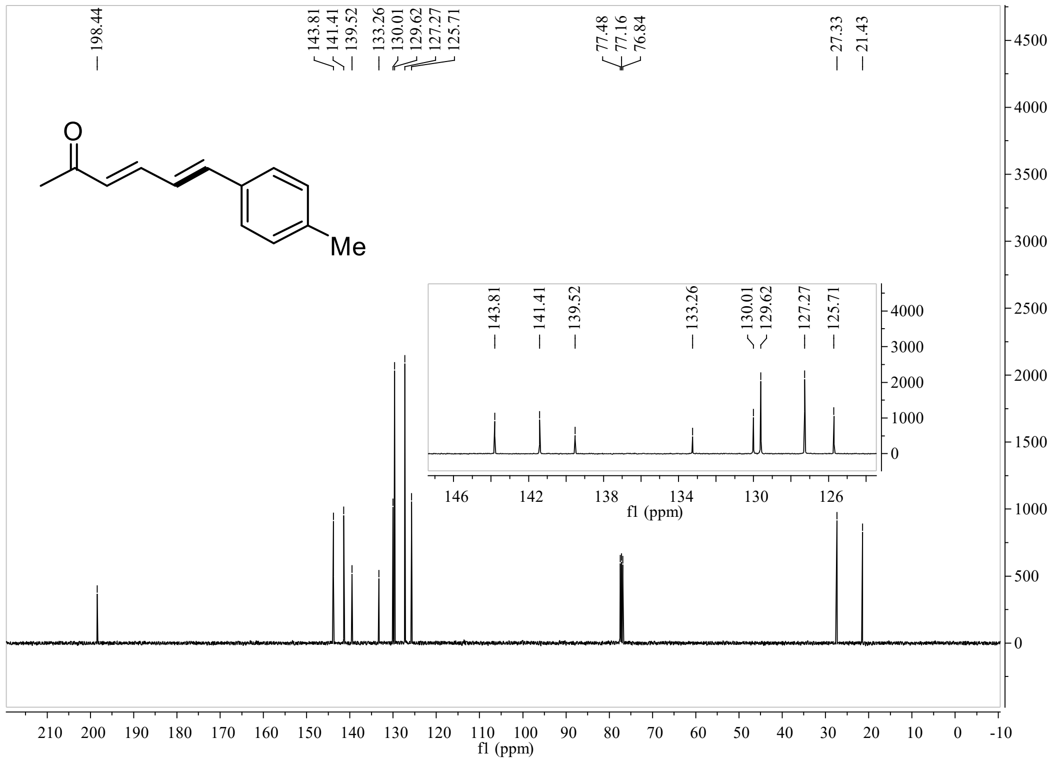

Figure S51 ^{13}C NMR spectrum of compound **2ap**, related to **Scheme 2**

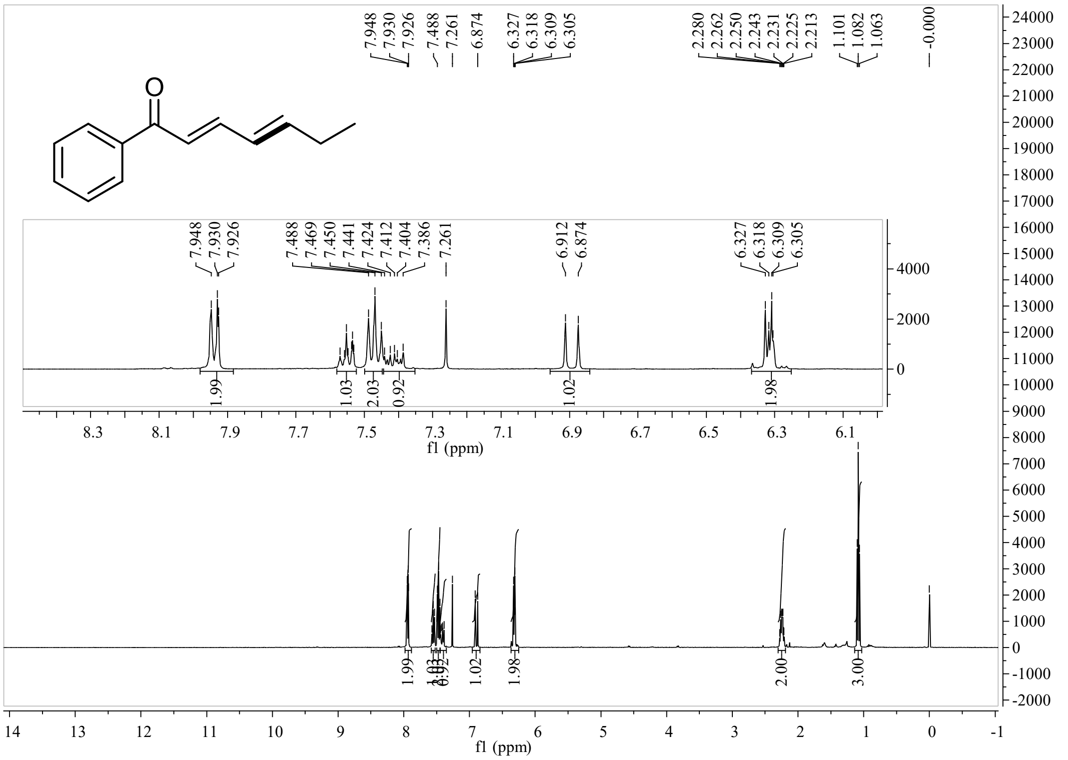

Figure S52 ^{1}H NMR spectrum of compound **2ba**, related to **Scheme 2**

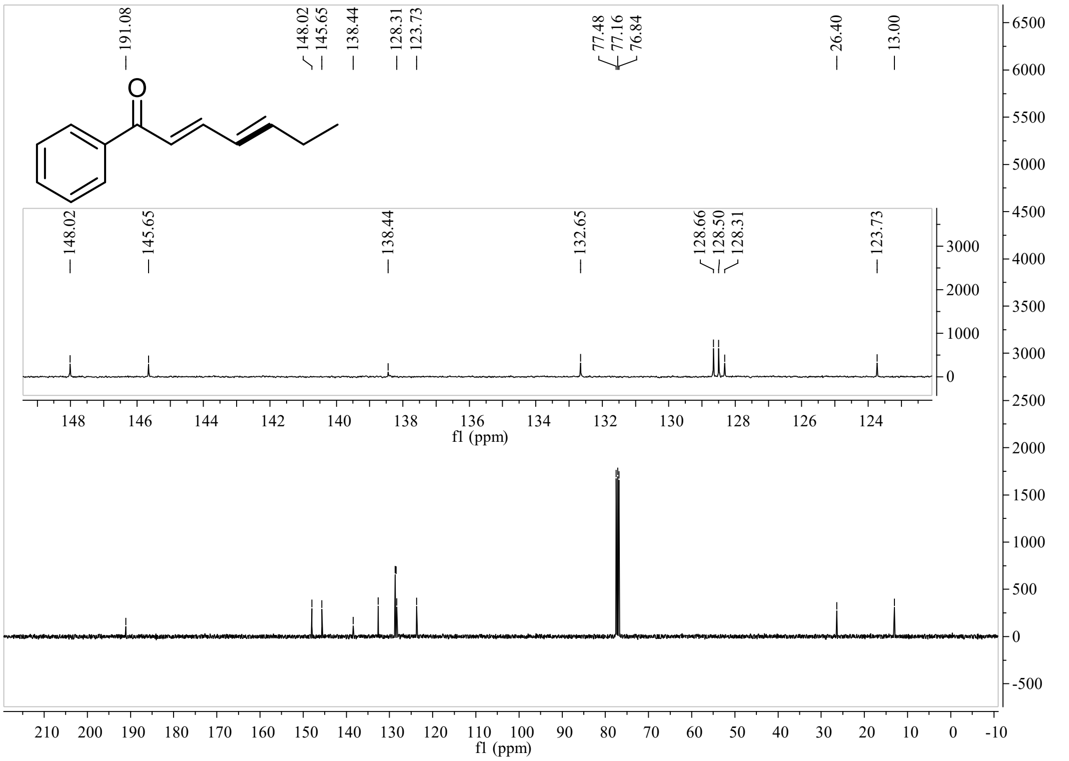

Figure S53 ^{13}C NMR spectrum of compound **2ba**, related to **Scheme 2**

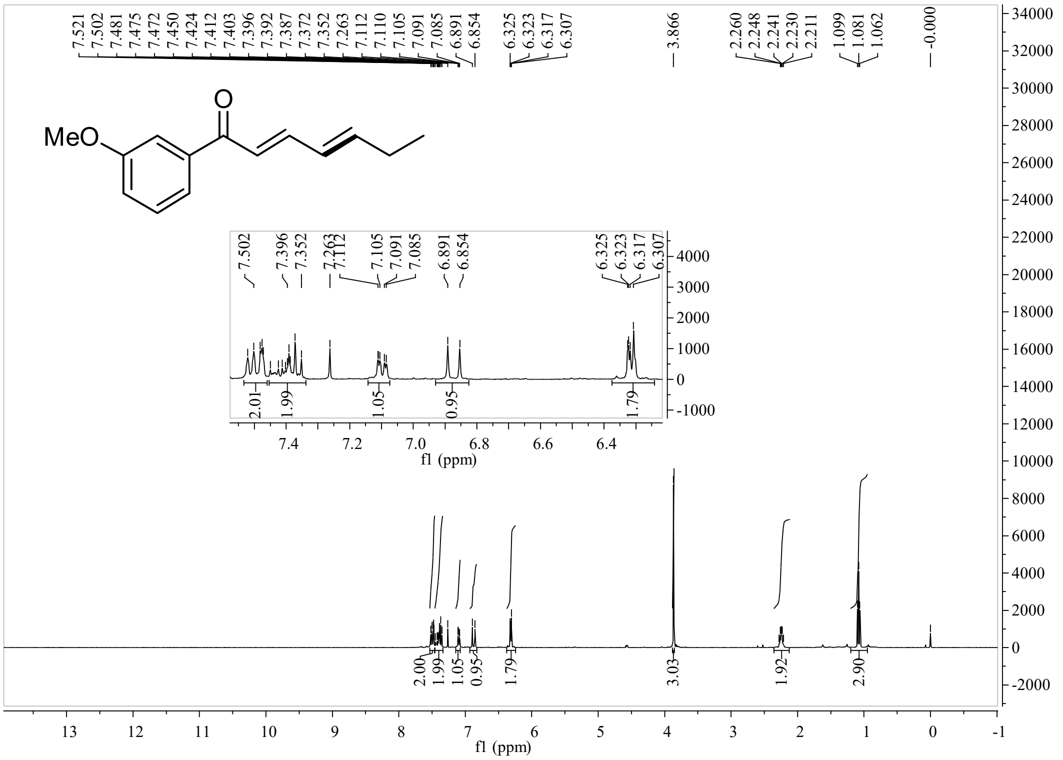

Figure S54 ^{1}H NMR spectrum of compound **2bb**, related to **Scheme 2**

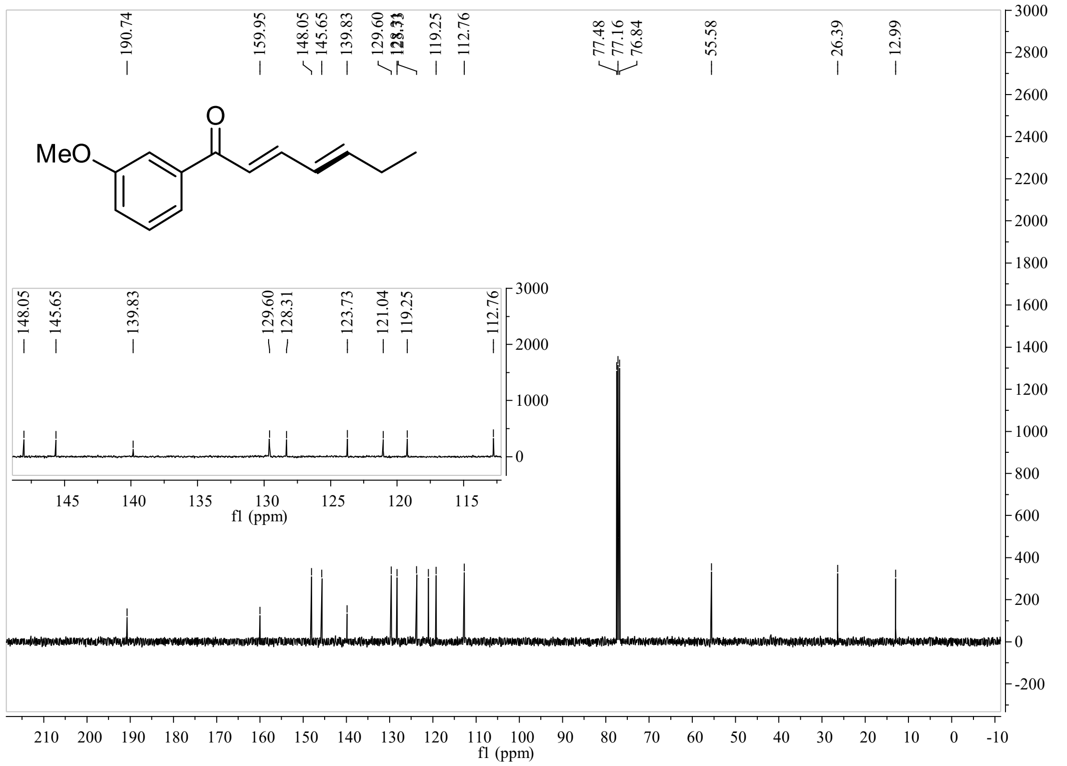

Figure S55 ^{13}C NMR spectrum of compound **2bb**, related to **Scheme 2**

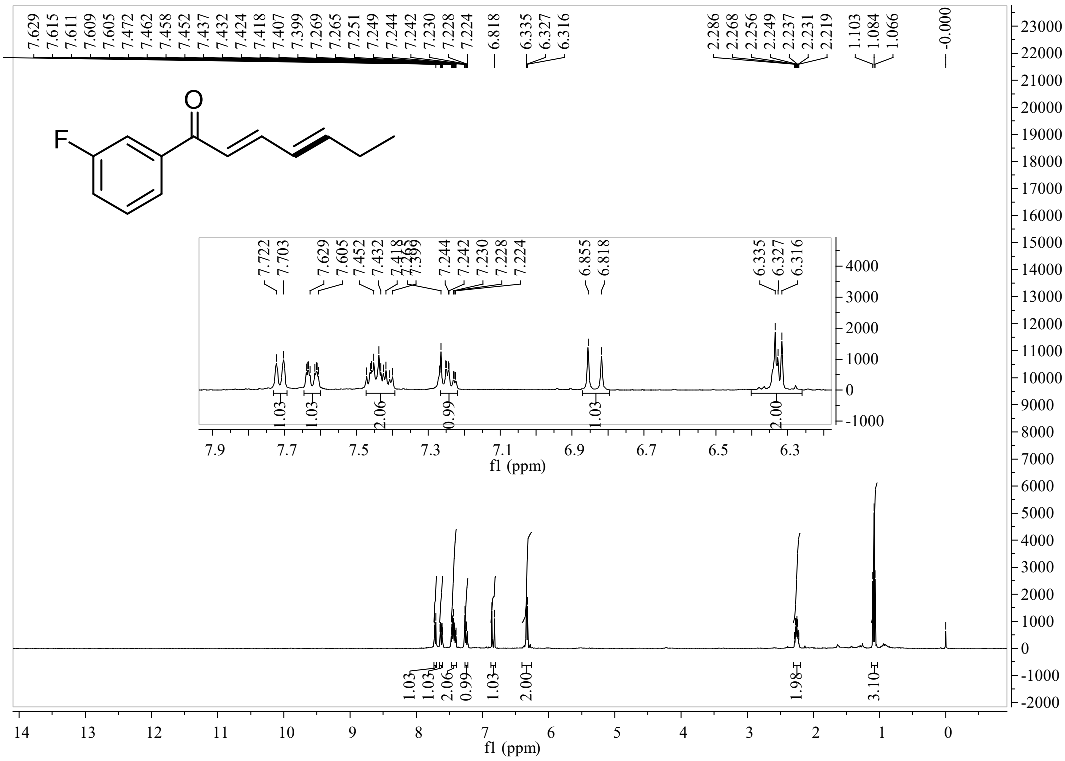

Figure S56 ^{1}H NMR spectrum of compound **2bc**, related to **Scheme 2**

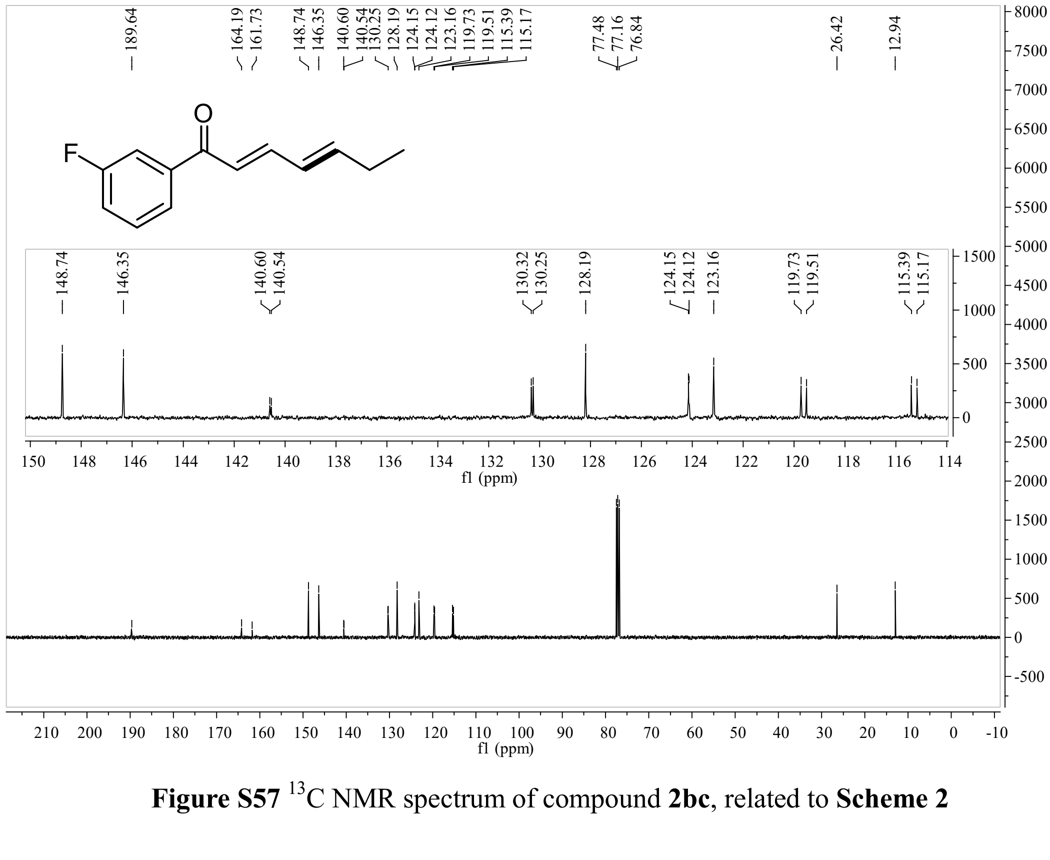

Figure S57 ^{13}C NMR spectrum of compound **2bc**, related to **Scheme 2**

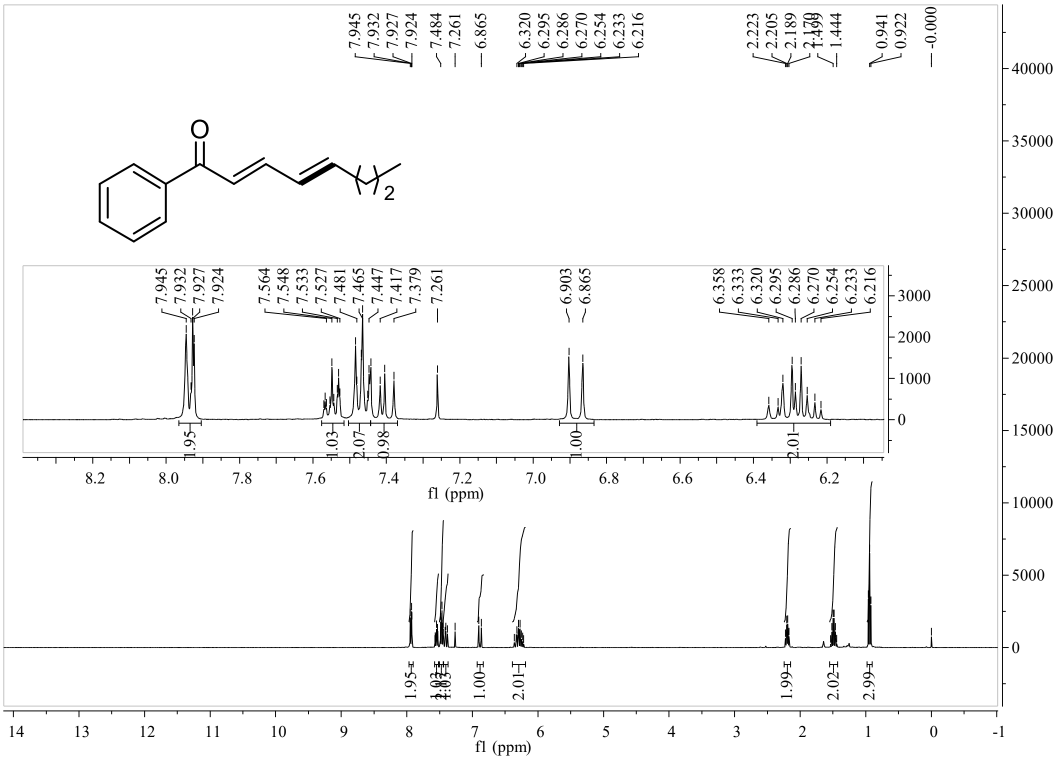

Figure S58 ^{1}H NMR spectrum of compound **2bd**, related to **Scheme 2**

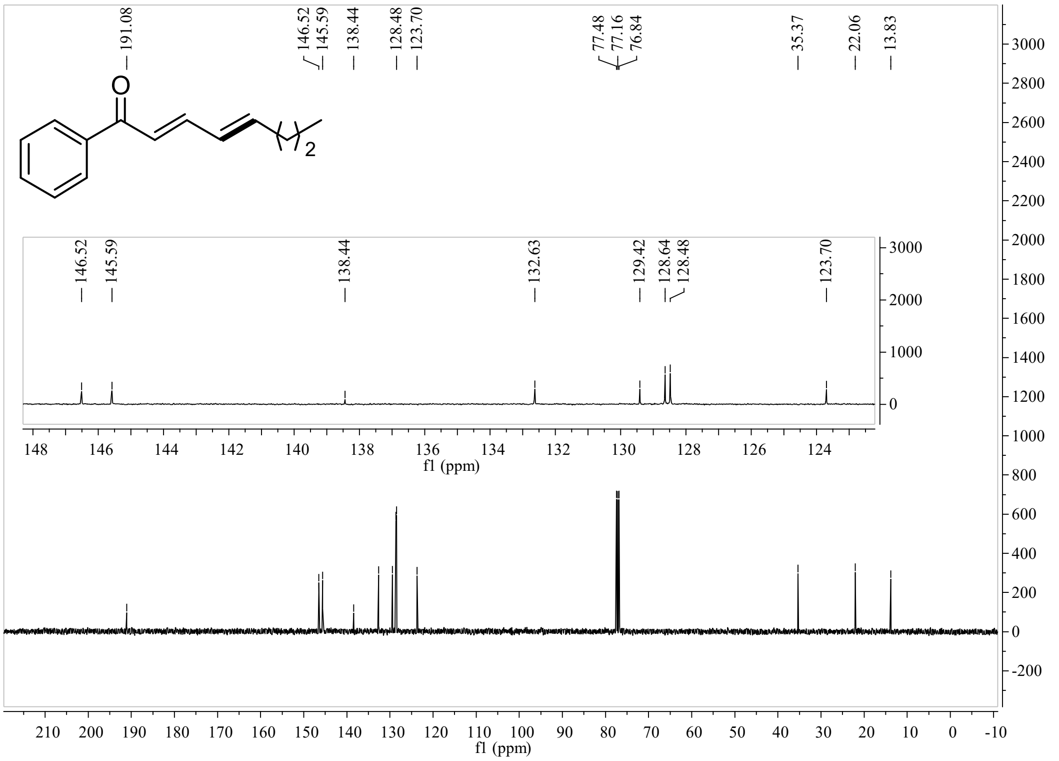

Figure S59 ^{13}C NMR spectrum of compound **2bd**, related to **Scheme 2**

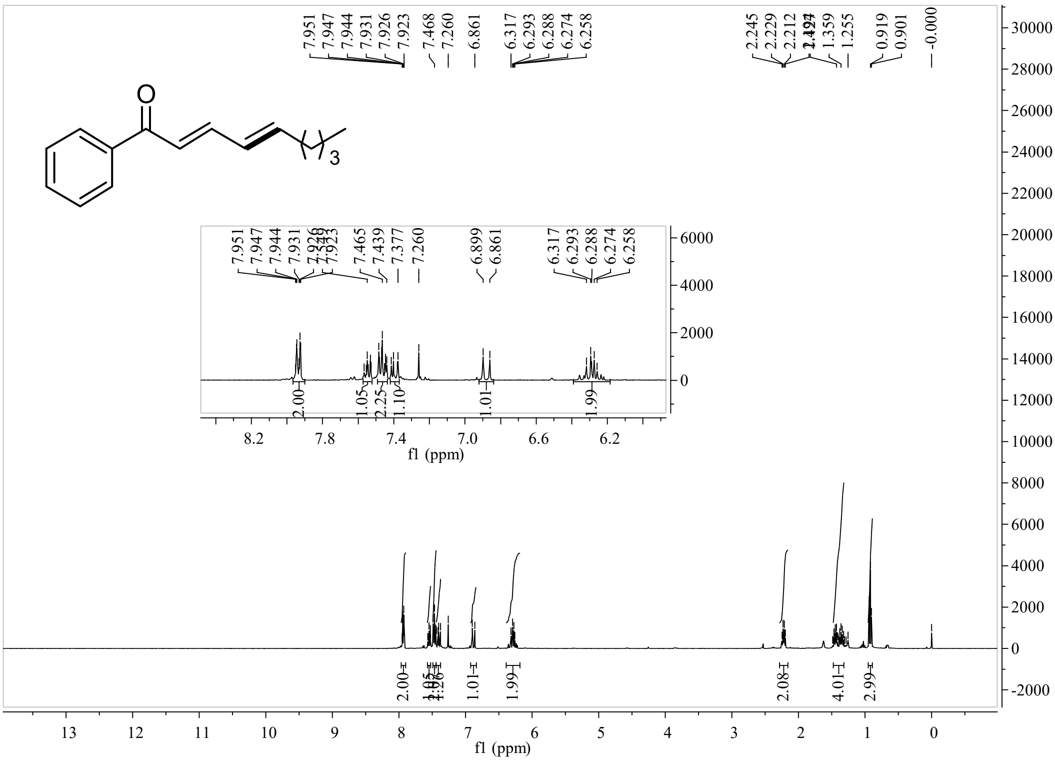

Figure S60 ^{1}H NMR spectrum of compound **2be**, related to **Scheme 2**

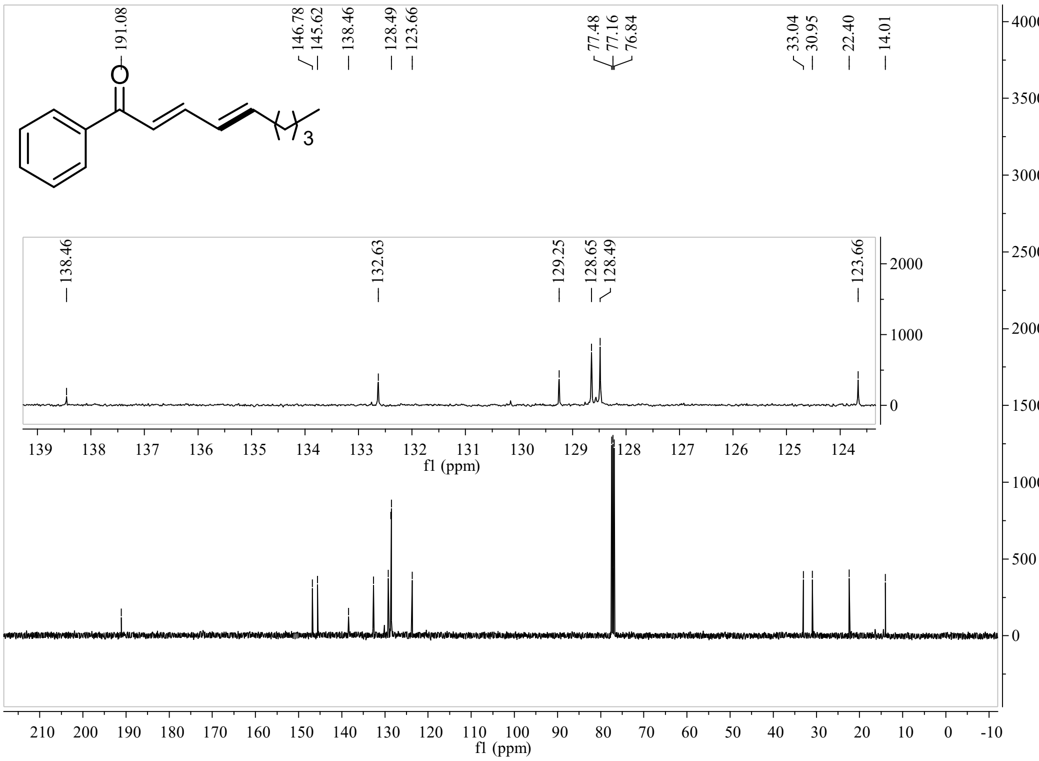

Figure S61 ^{13}C NMR spectrum of compound **2be**, related to **Scheme 2**

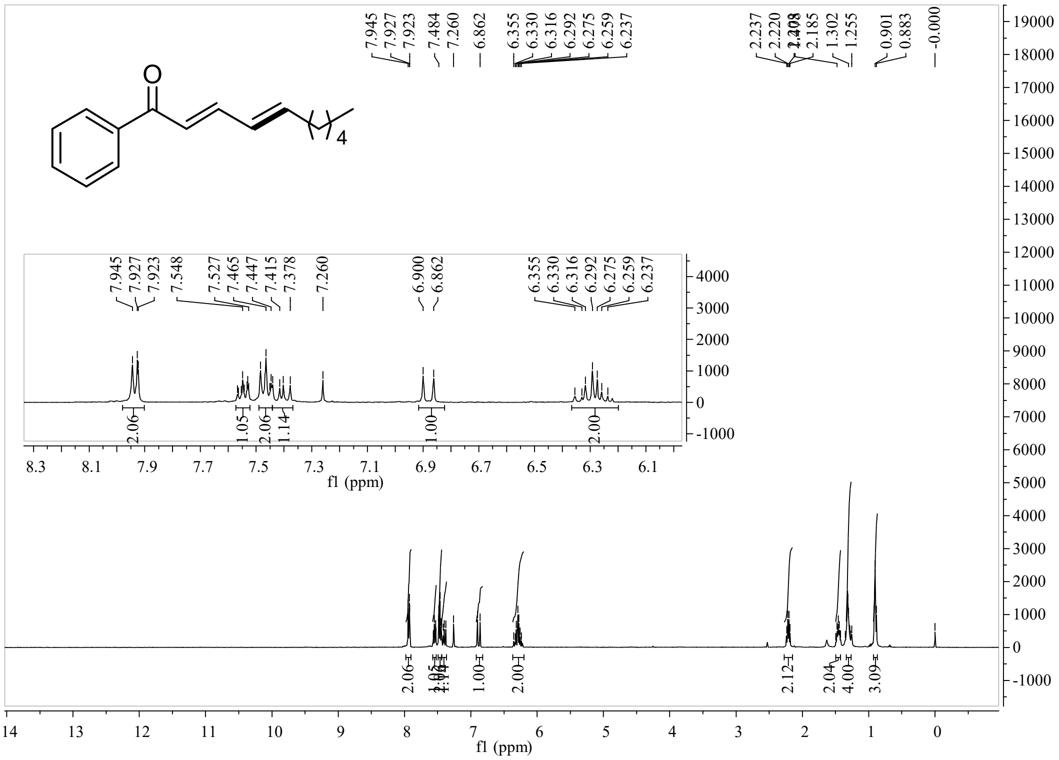

Figure S62 ^{1}H NMR spectrum of compound **2bf**, related to **Scheme 2**

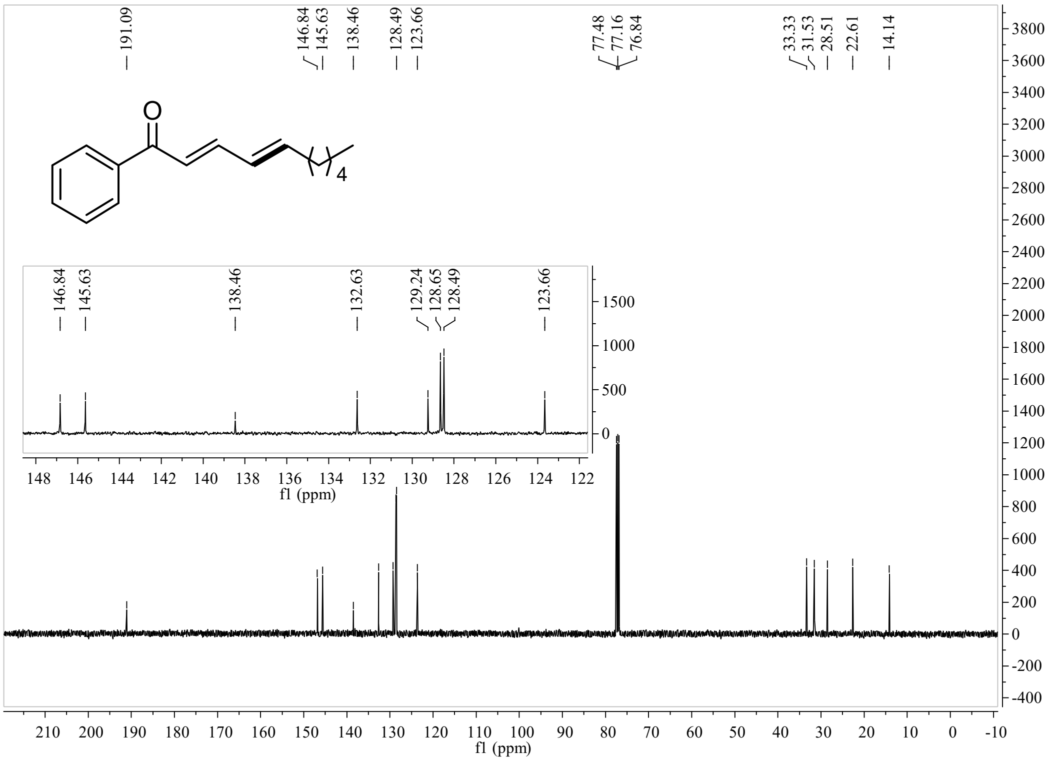

Figure S63 ^{13}C NMR spectrum of compound **2bf**, related to **Scheme 2**

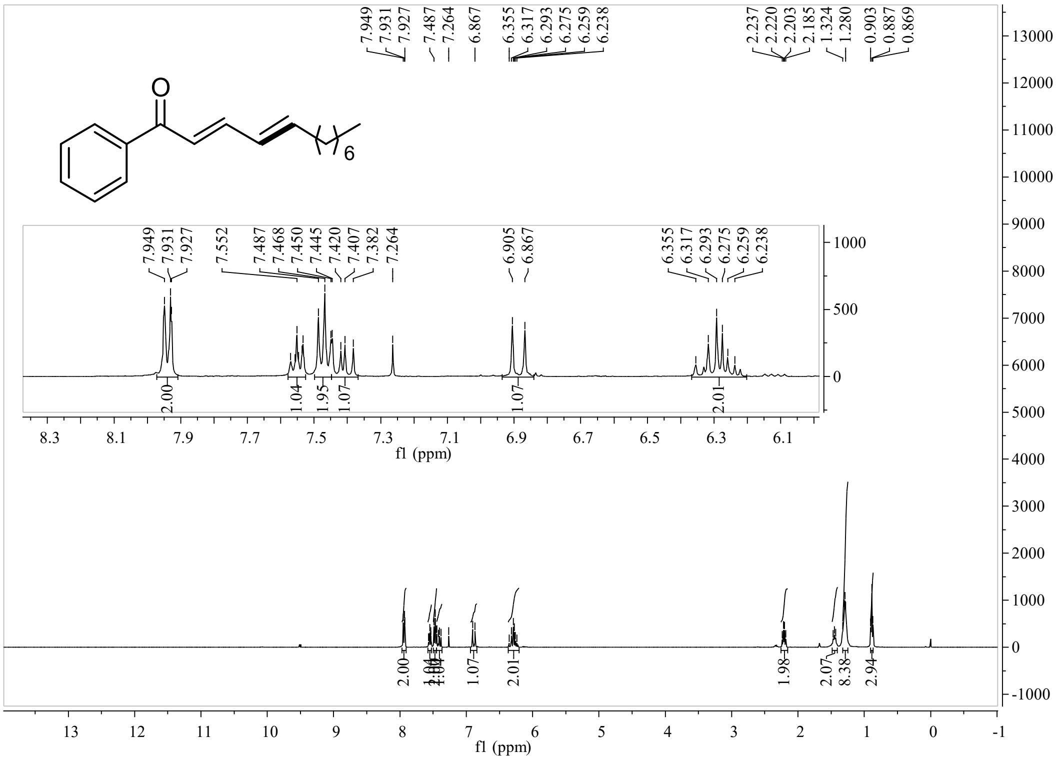

Figure S64 ^{1}H NMR spectrum of compound **2bg**, related to **Scheme 2**

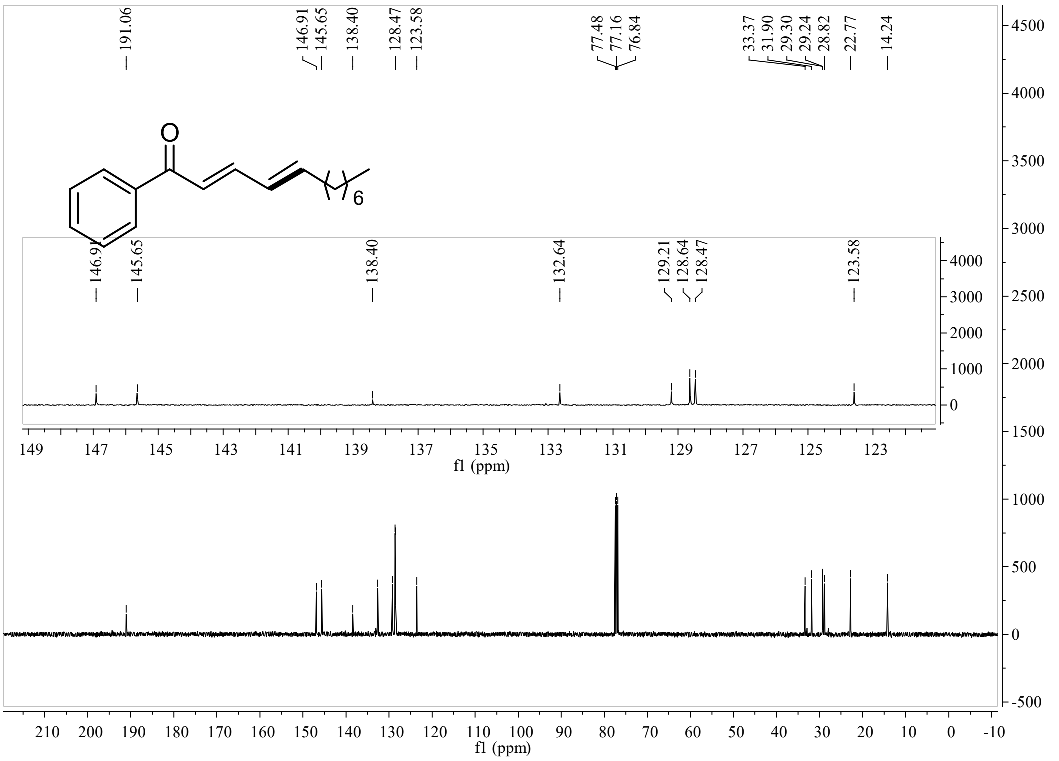

Figure S65 ^{13}C NMR spectrum of compound **2bg**, related to **Scheme 2**

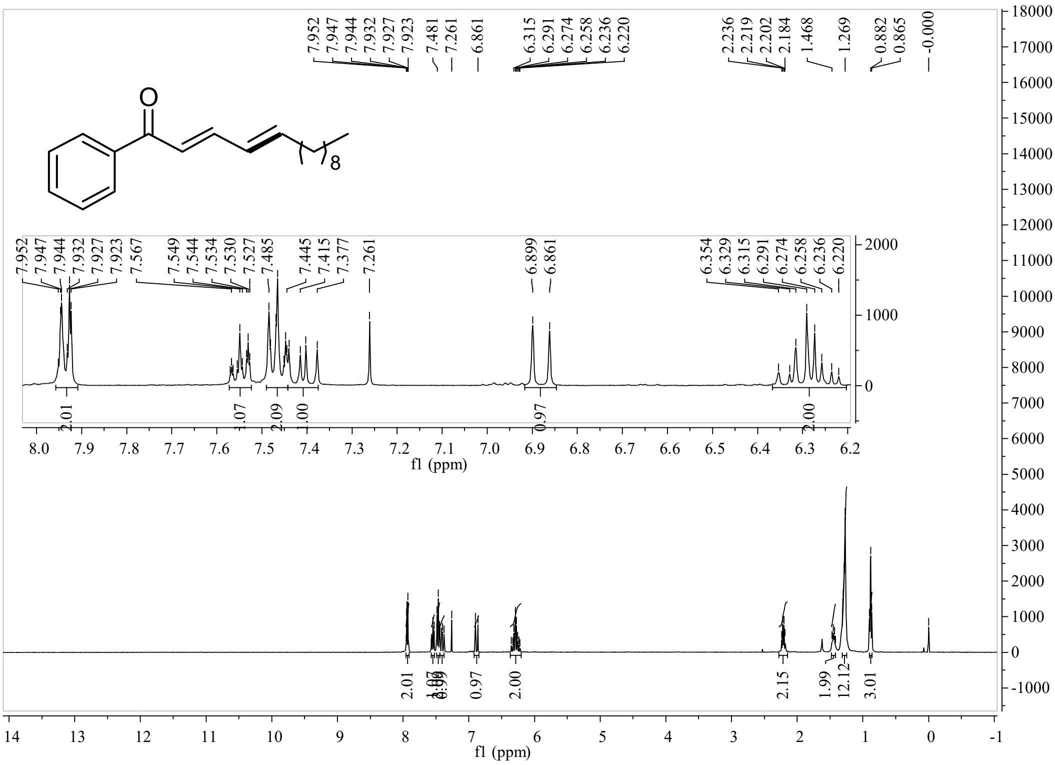

Figure S66 ^{1}H NMR spectrum of compound **2bh**, related to **Scheme 2**

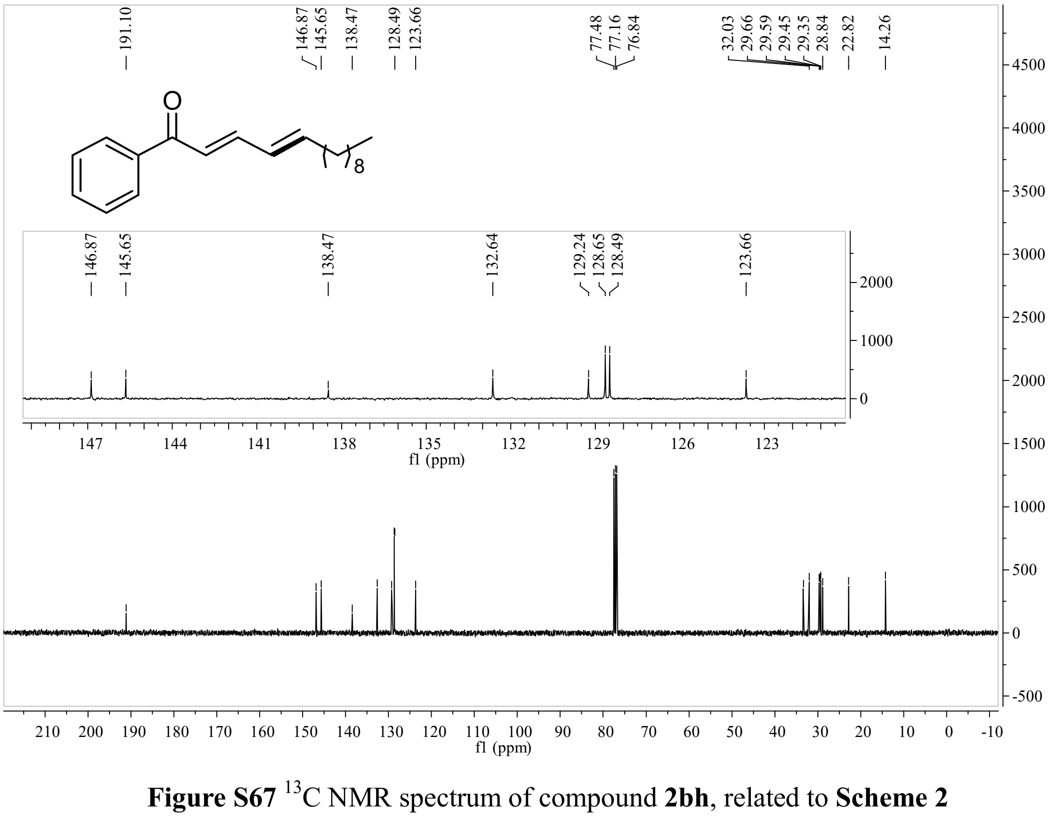

Figure S67 ^{13}C NMR spectrum of compound **2bh**, related to **Scheme 2**

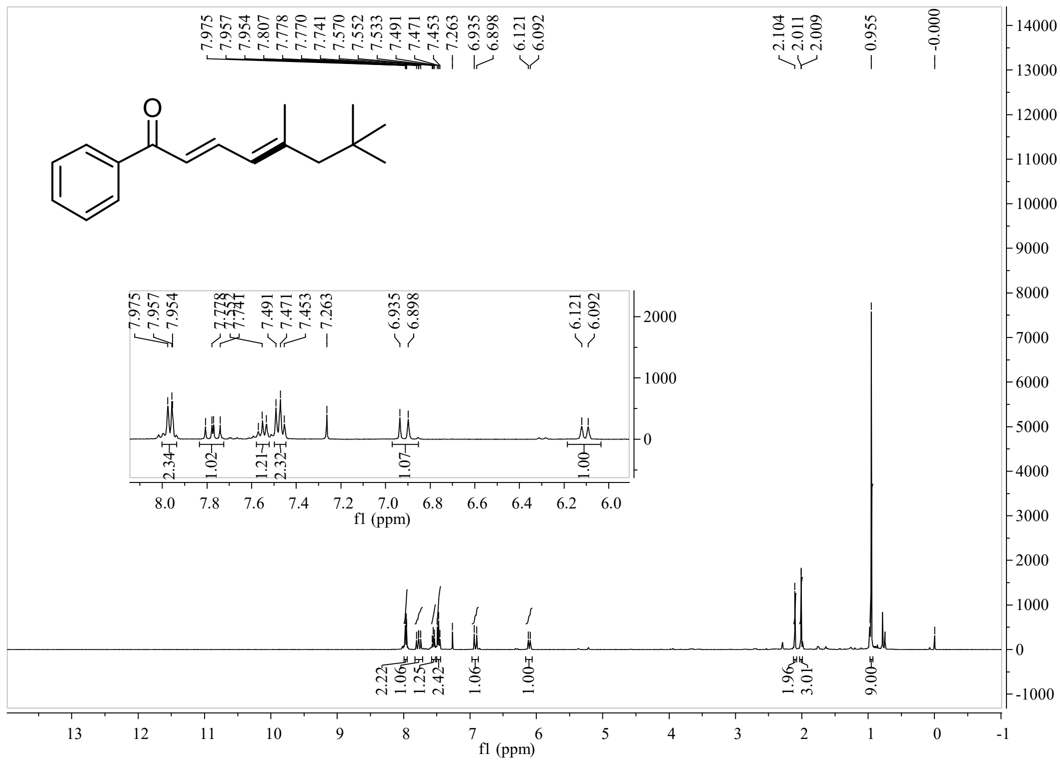

Figure S68 ^{1}H NMR spectrum of compound **2bi**, related to **Scheme 2**

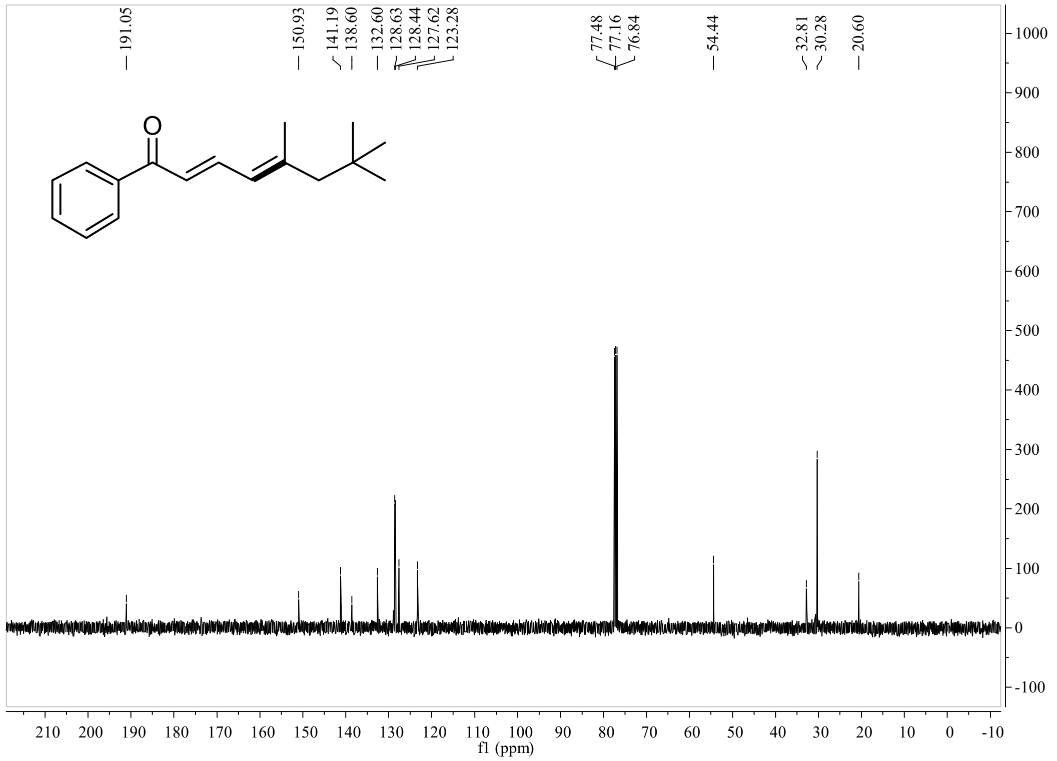

Figure S69 ^{13}C NMR spectrum of compound **2bi**, related to **Scheme 2**

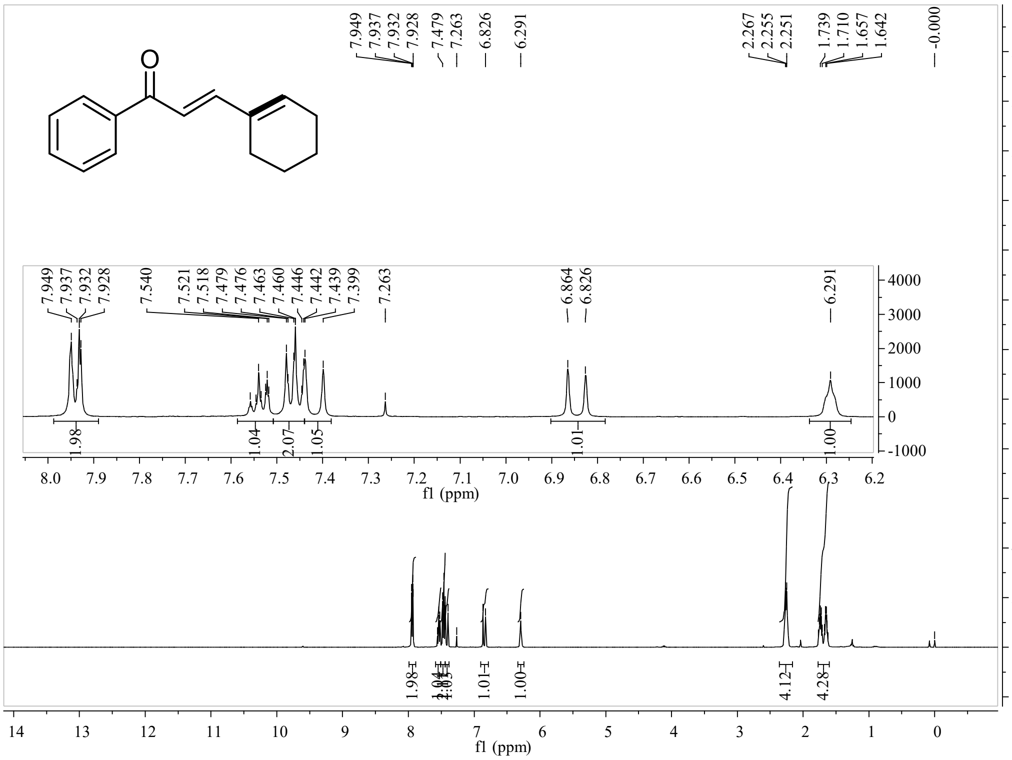

Figure S70 ^{1}H NMR spectrum of compound **2bj**, related to **Scheme 2**

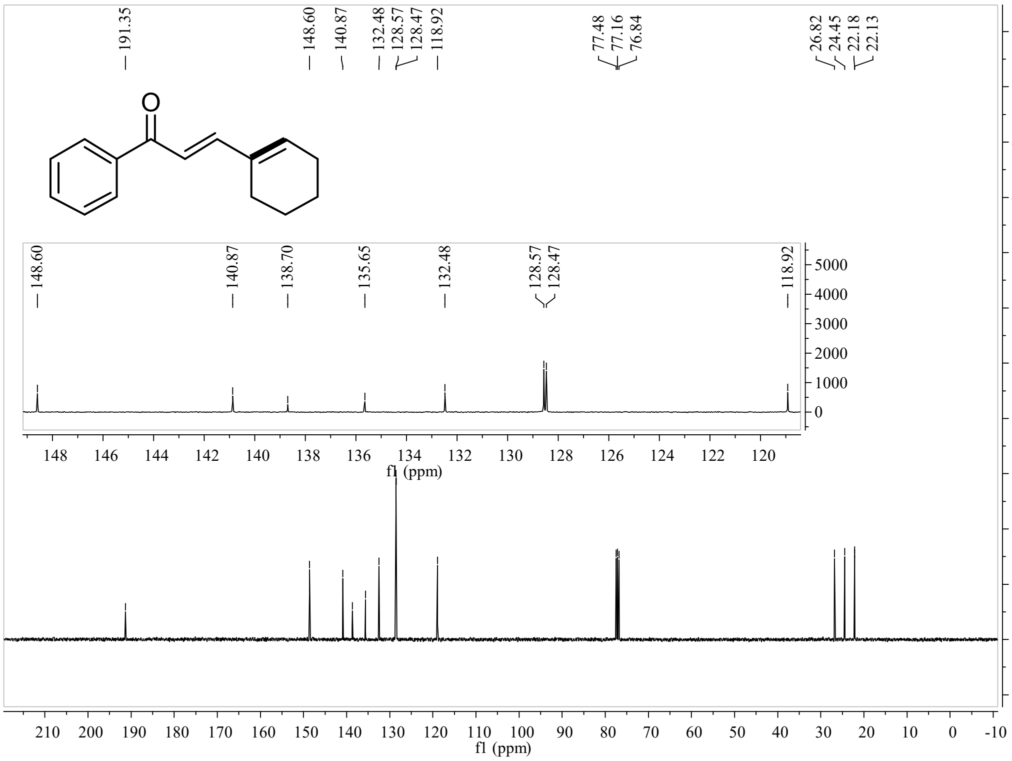

Figure S71 ^{13}C NMR spectrum of compound **2bj**, related to **Scheme 2**

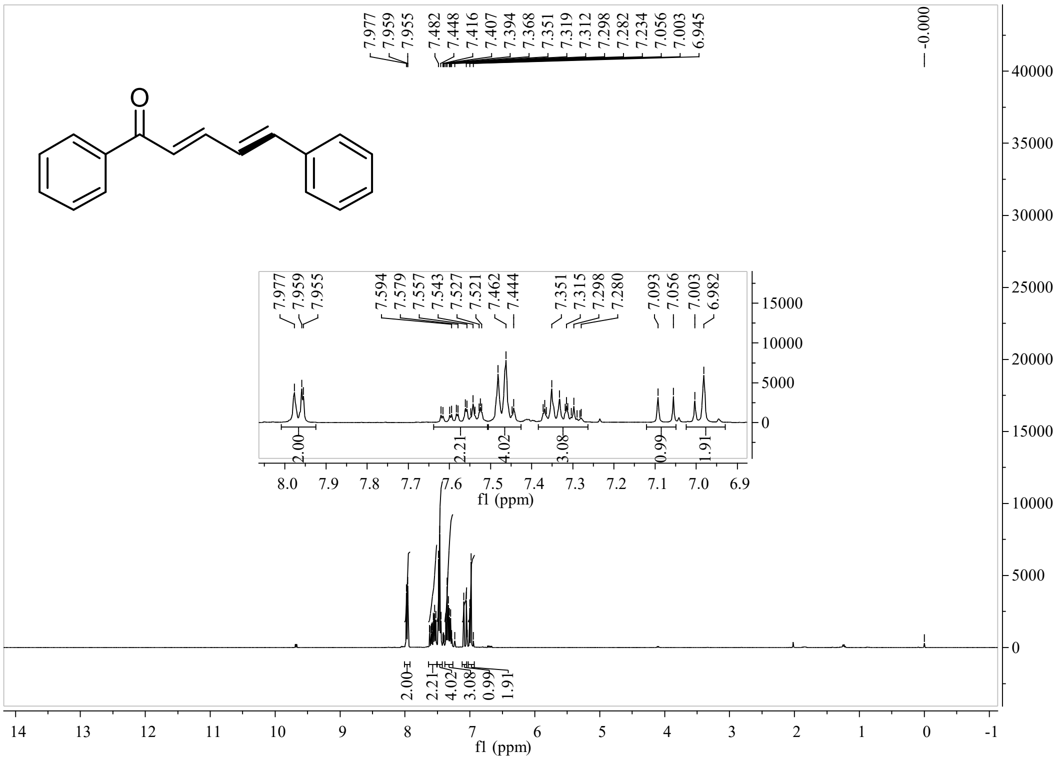

Figure S72 ^{1}H NMR spectrum of compound **2bk**, related to **Scheme 2**

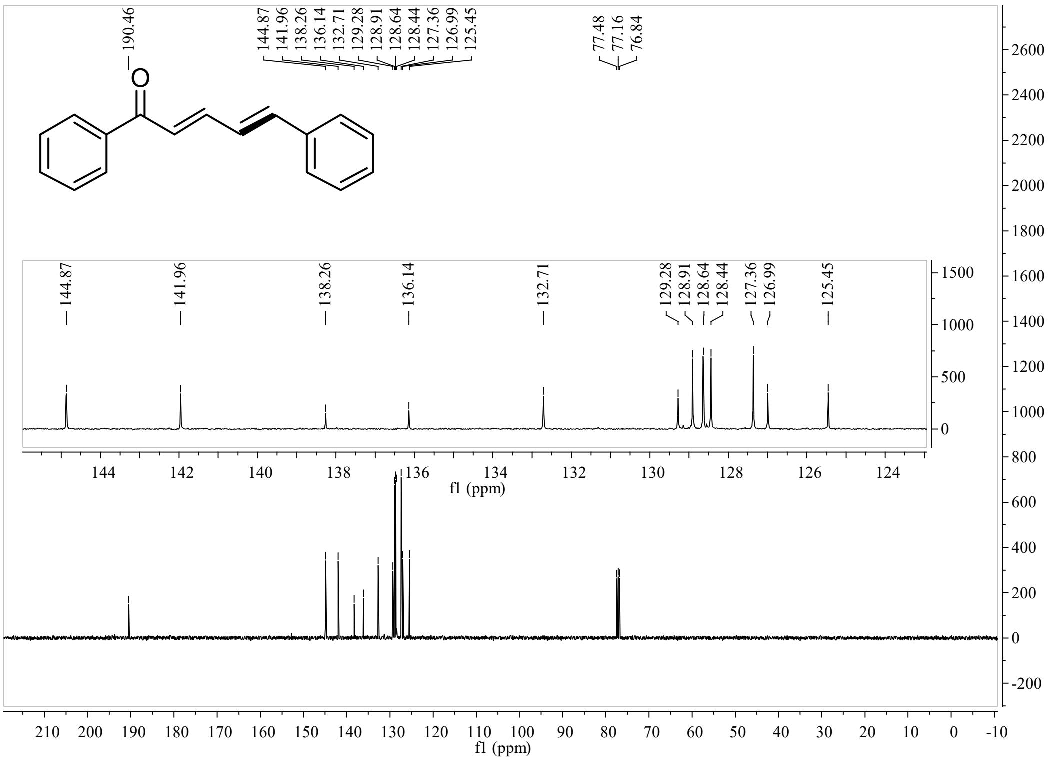

Figure S73 ^{13}C NMR spectrum of compound **2bk**, related to **Scheme 2**

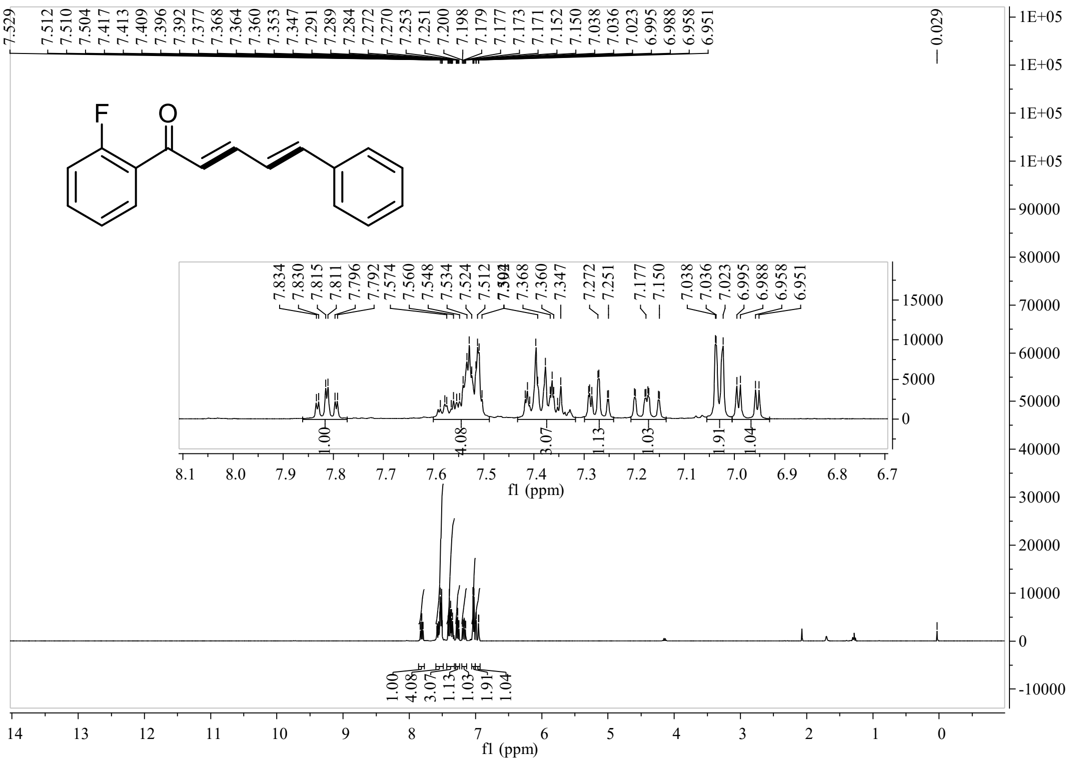

Figure S74 ^{1}H NMR spectrum of compound **2bl**, related to **Scheme 2**

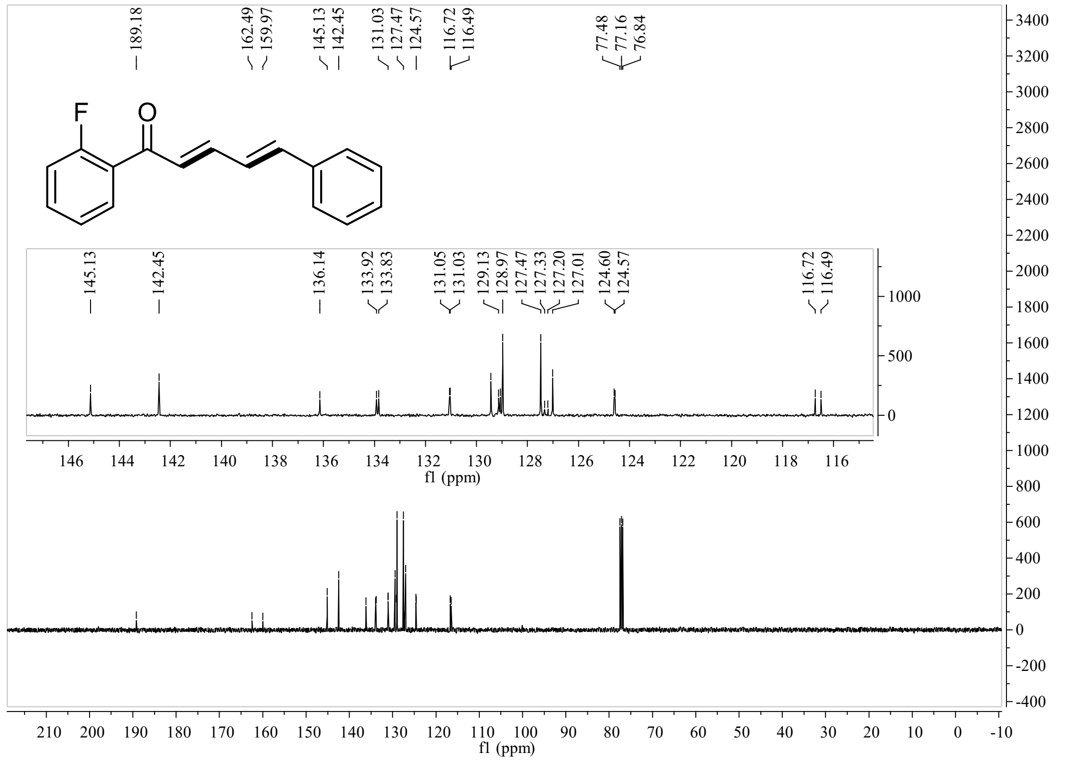

Figure S75 ^{13}C NMR spectrum of compound **2bl**, related to **Scheme 2**

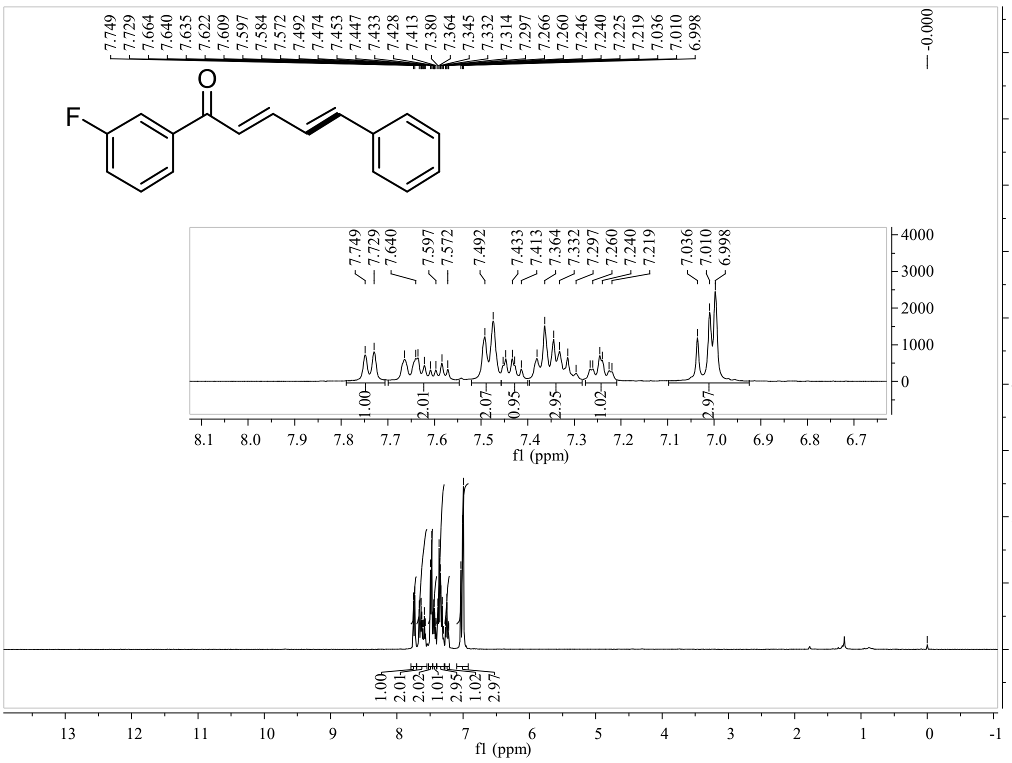

Figure S76 ^{1}H NMR spectrum of compound **2bm**, related to **Scheme 2**

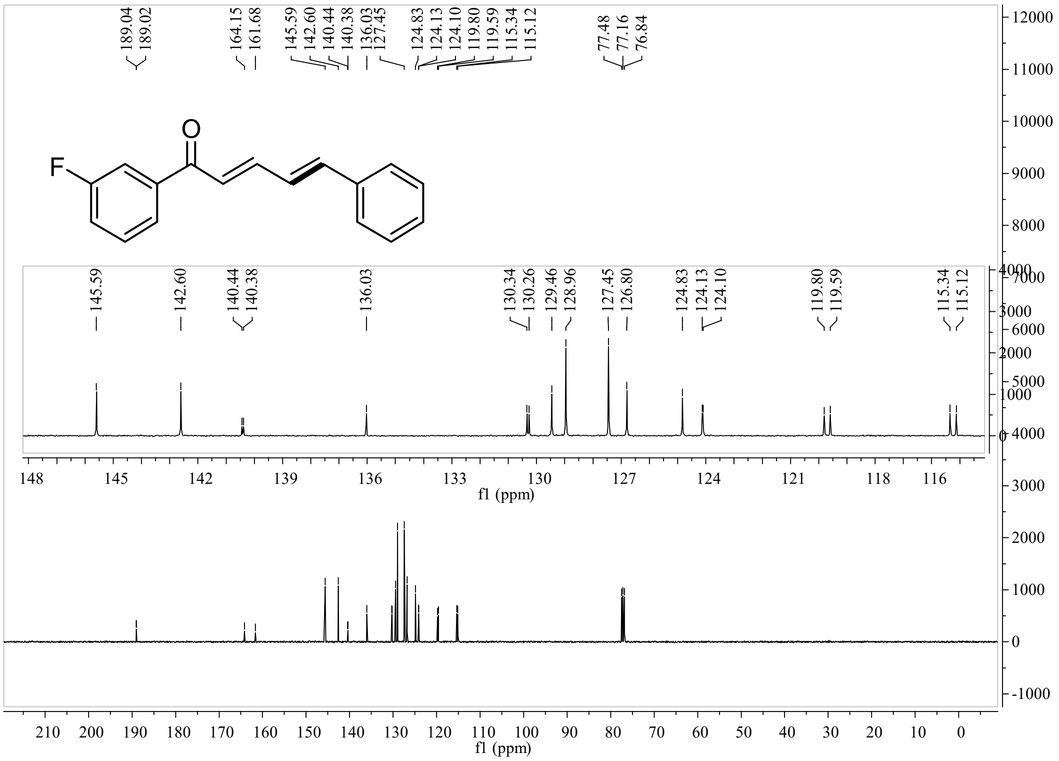

Figure S77 ^{13}C NMR spectrum of compound **2bm**, related to **Scheme 2**

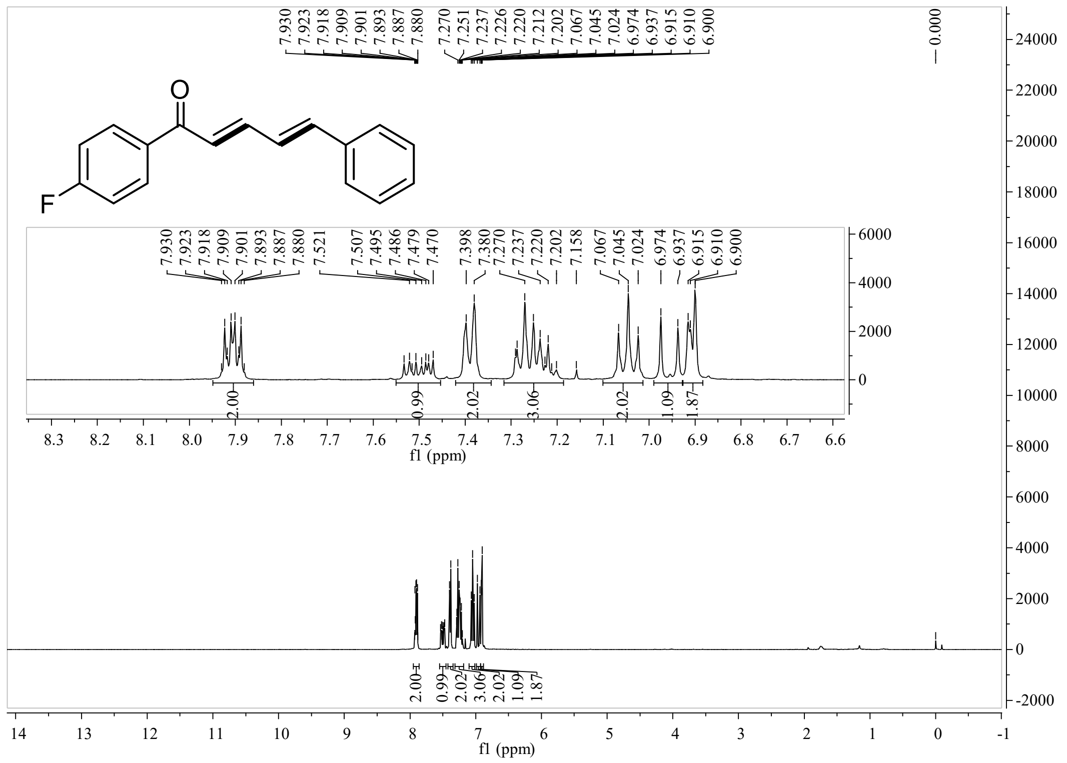

Figure S78 ^{1}H NMR spectrum of compound **2bm**, related to **Scheme 2**

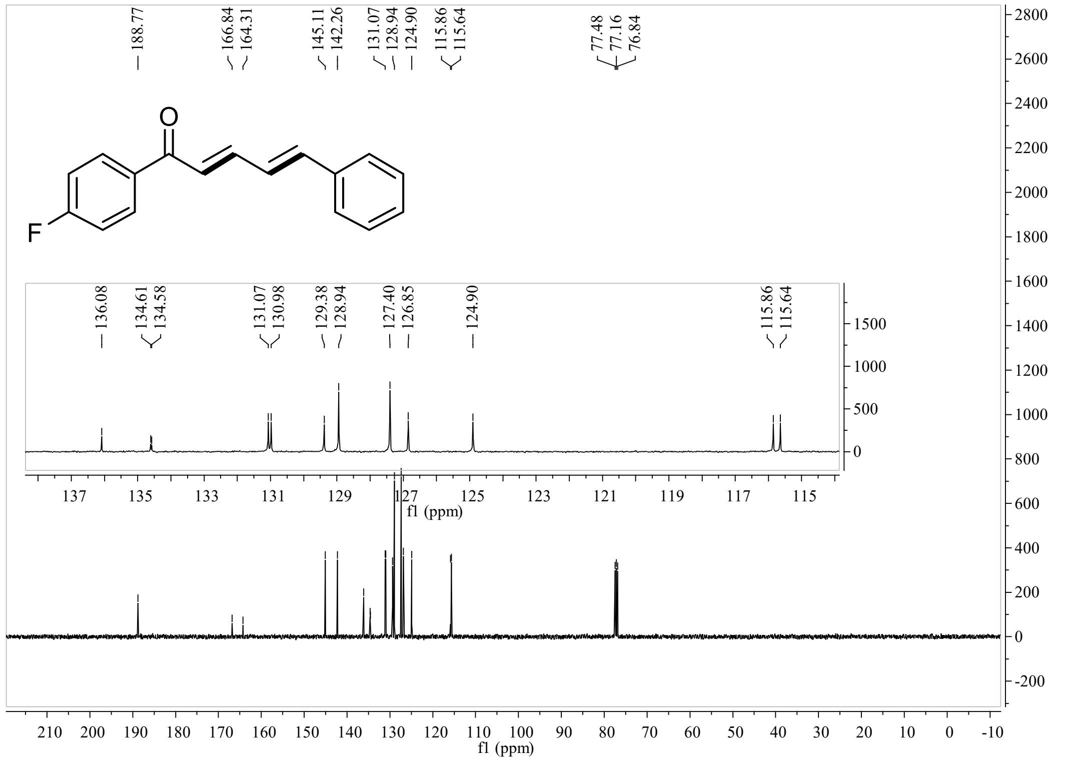

Figure S79 ^{13}C NMR spectrum of compound **2bm**, related to **Scheme 2**

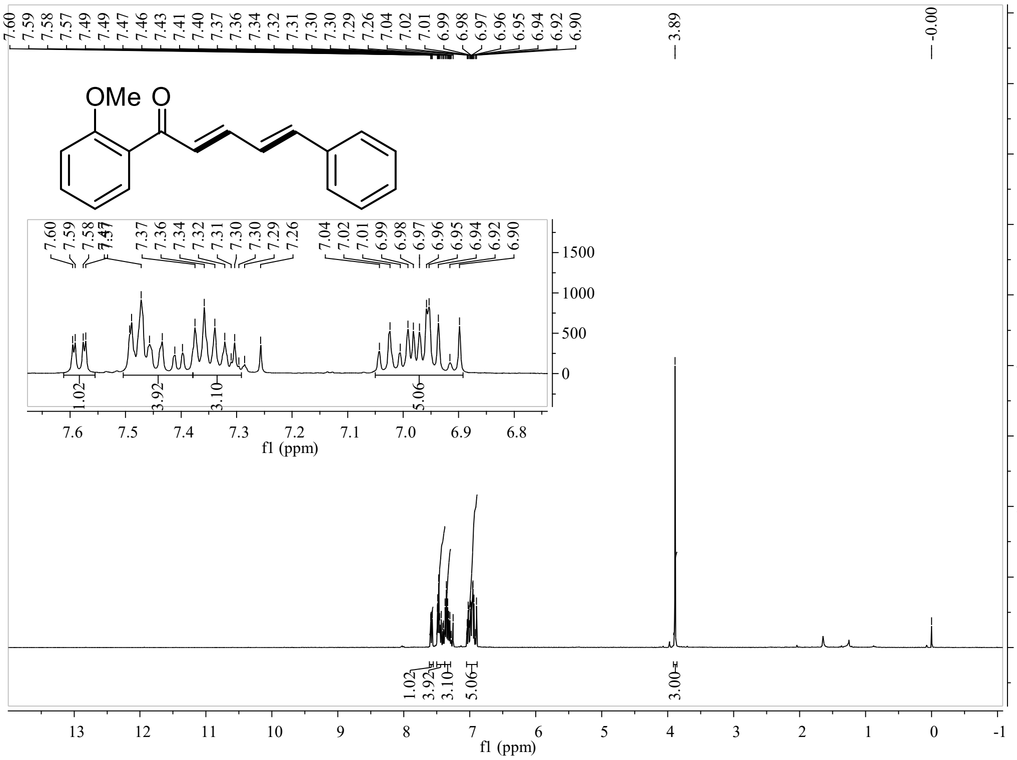

Figure S80 ^{1}H NMR spectrum of compound **2bo**, related to **Scheme 2**

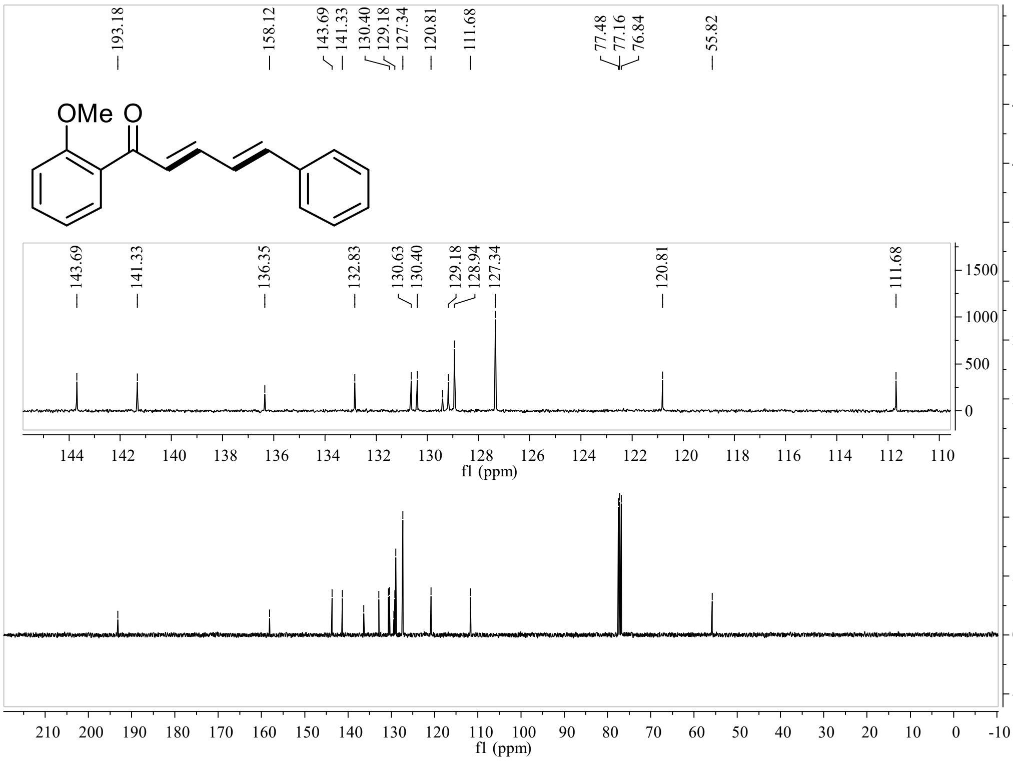

Figure S81 ^{13}C NMR spectrum of compound **2bo**, related to **Scheme 2**

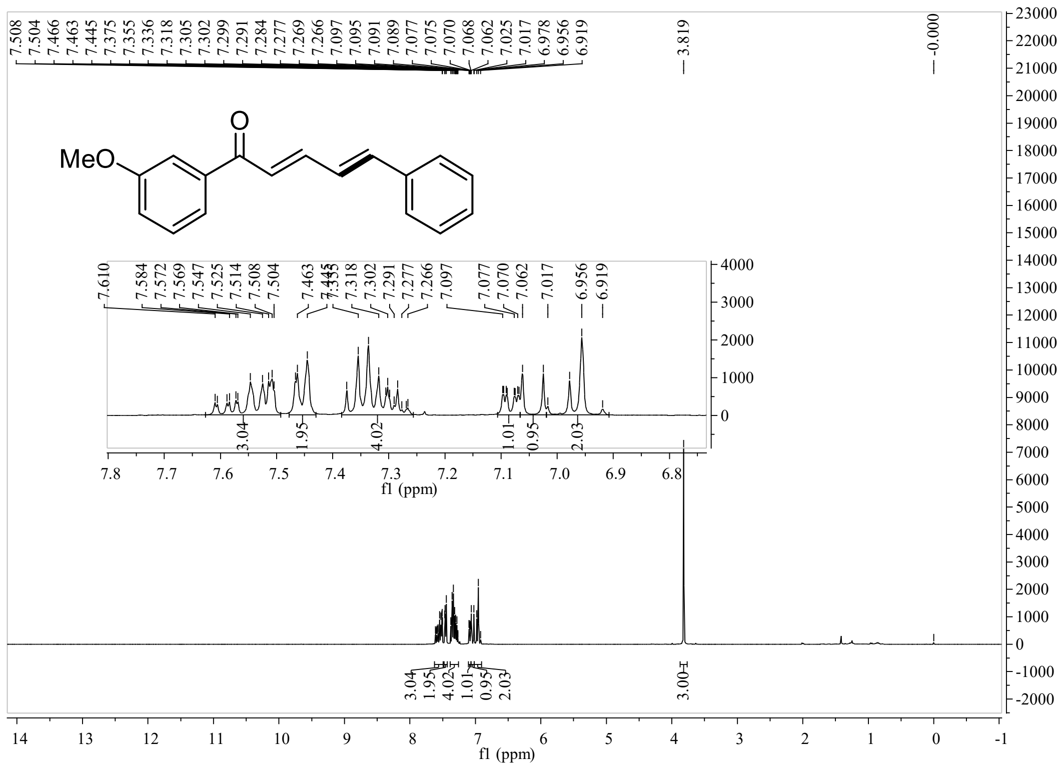

Figure S82 ^{1}H NMR spectrum of compound **2bp**, related to **Scheme 2**

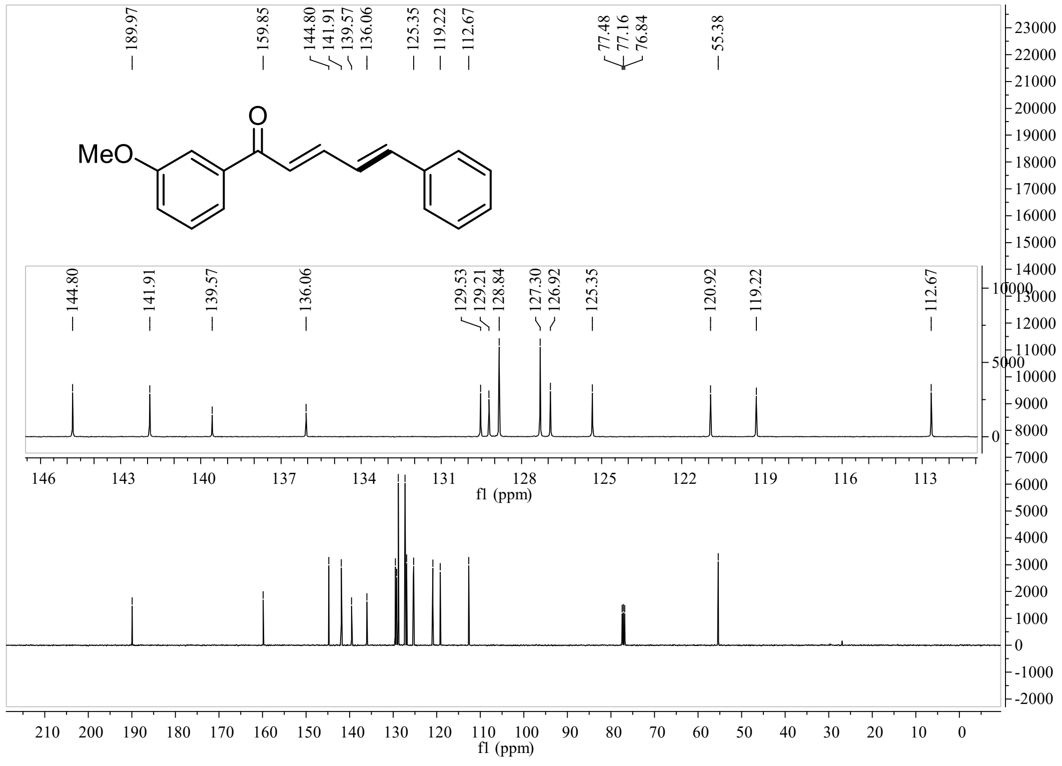

Figure S83 ^{13}C NMR spectrum of compound **2bp**, related to **Scheme 2**

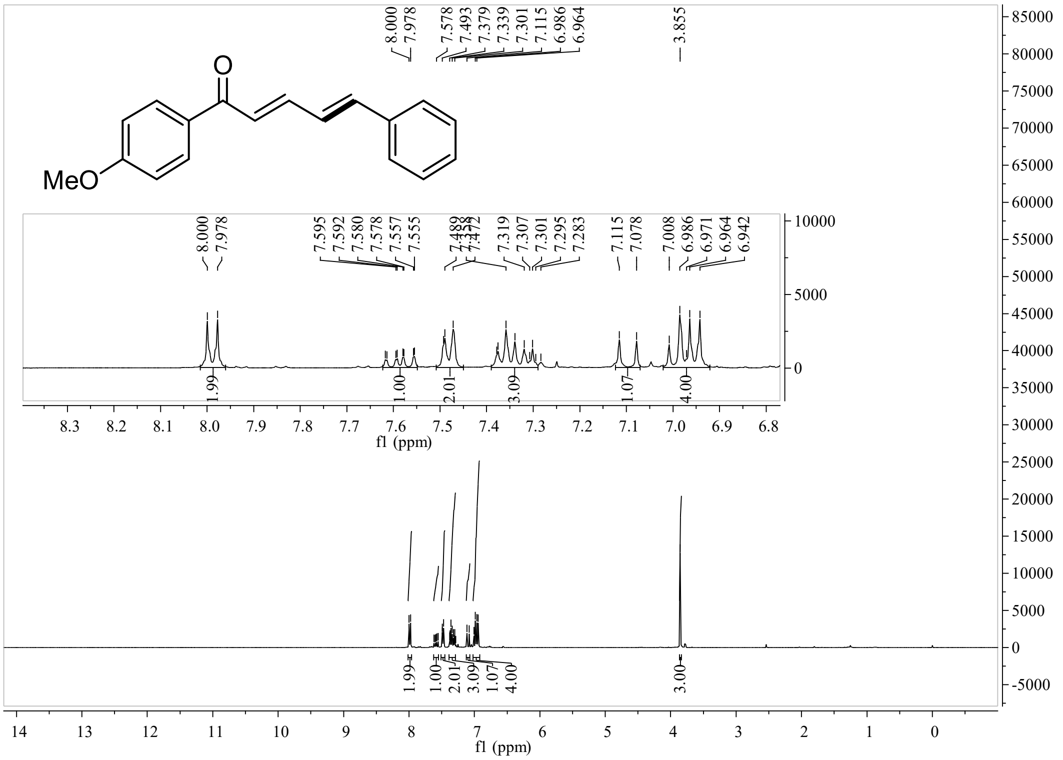

Figure S84 ^{1}H NMR spectrum of compound **2bq**, related to **Scheme 2**

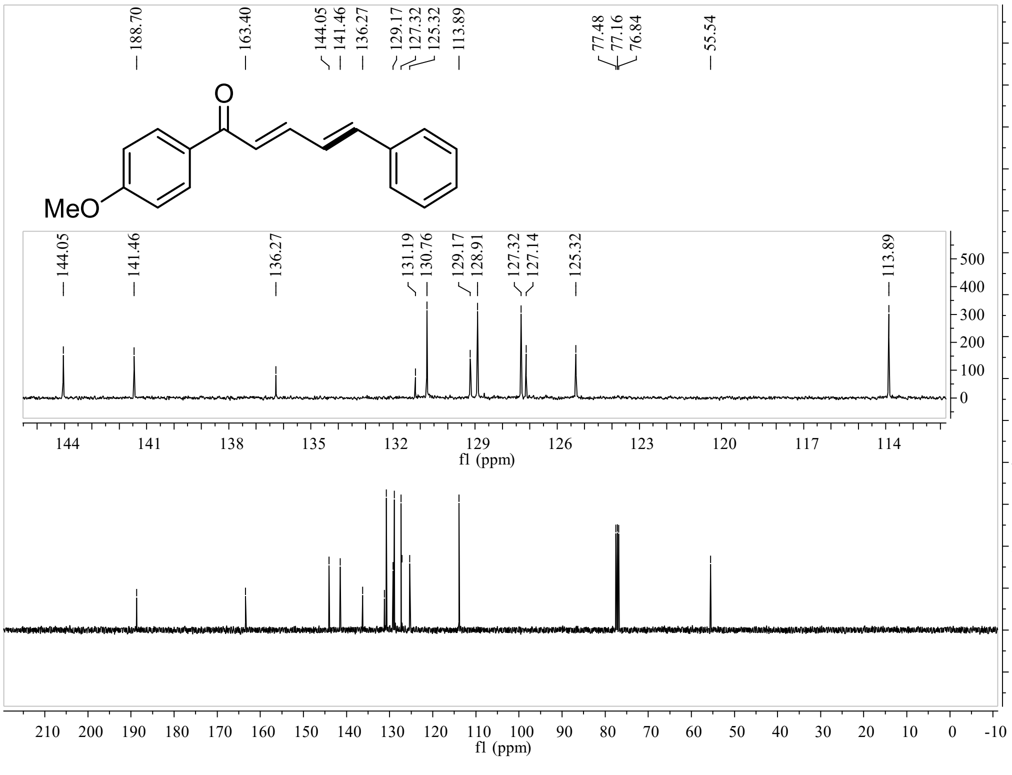

Figure S85 ^{13}C NMR spectrum of compound **2bq**, related to **Scheme 2**

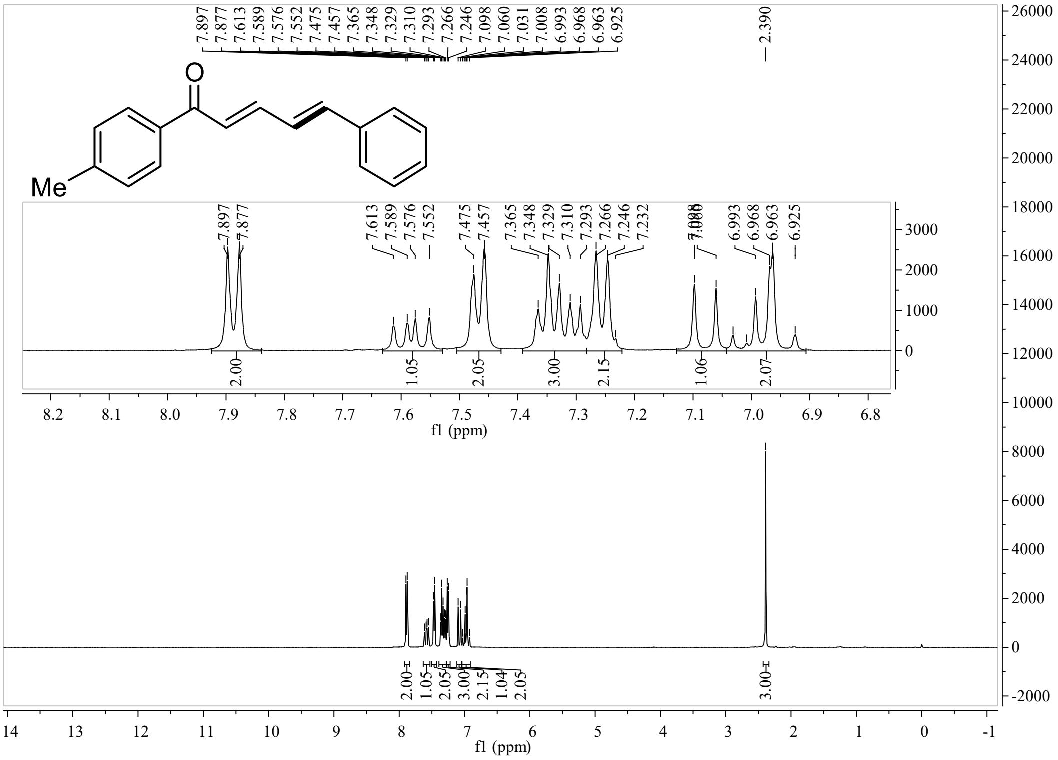

Figure S86^{1}H NMR spectrum of compound **2br**, related to **Scheme 2**

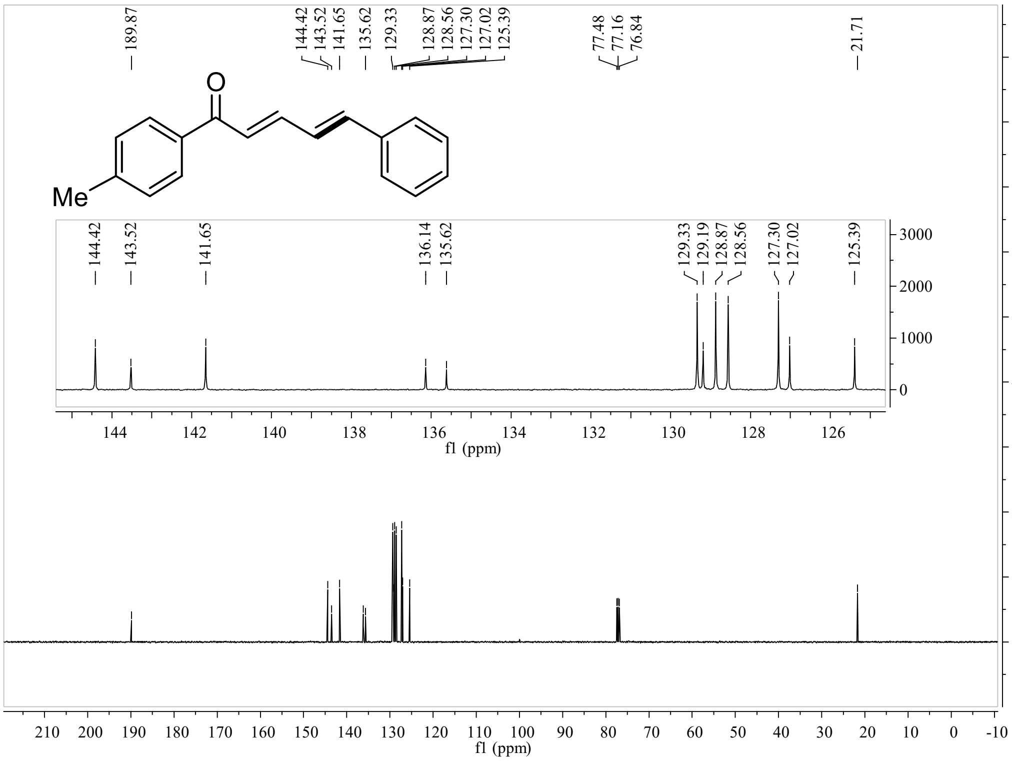

Figure S87 ^{13}C NMR spectrum of compound **2br**, related to **Scheme 2**

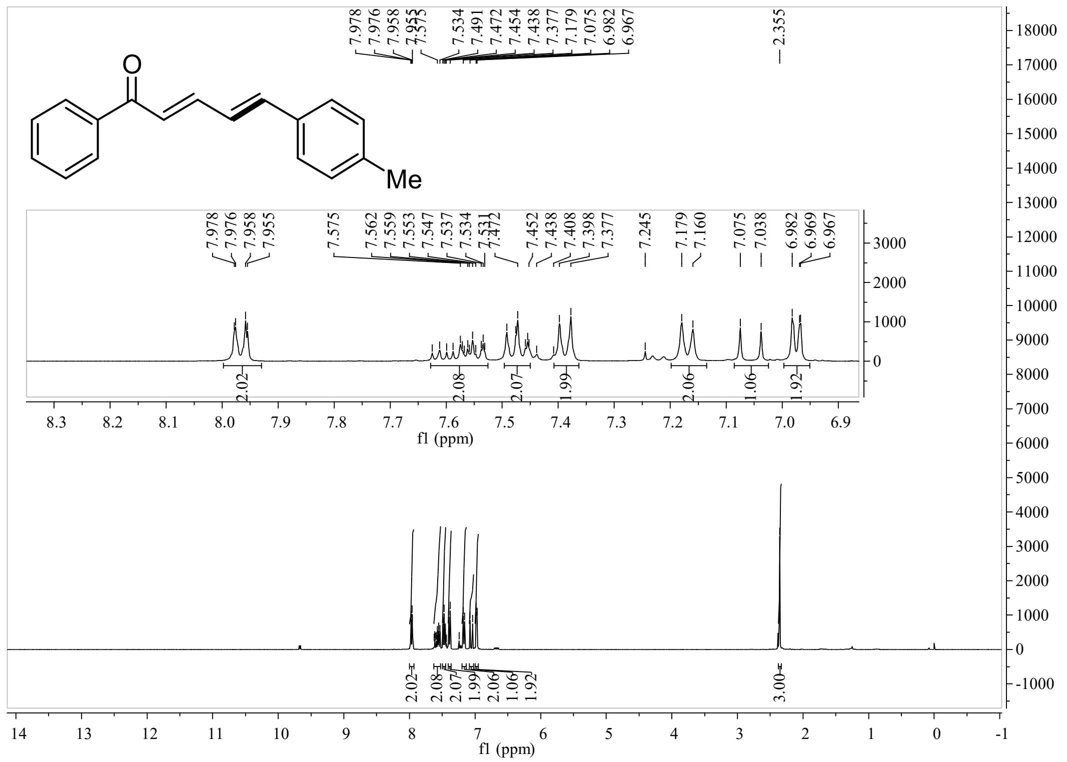

Figure S88 ^{1}H NMR spectrum of compound **2bs**, related to **Scheme 2**

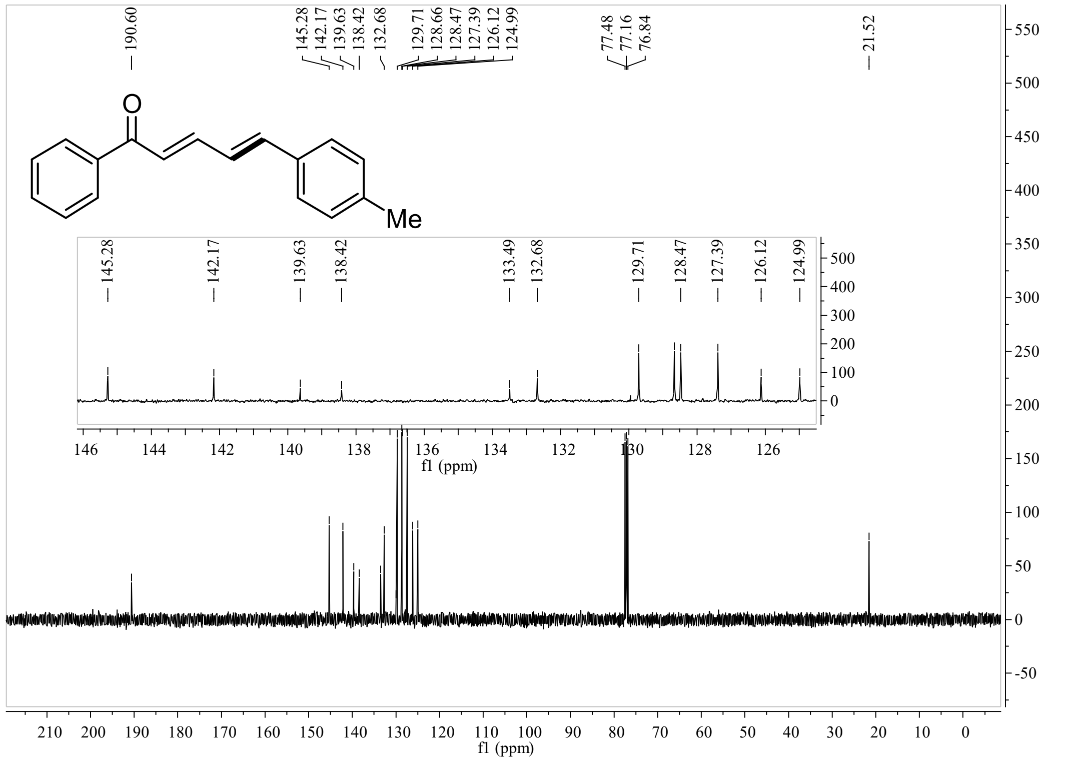

Figure S89 ^{13}C NMR spectrum of compound **2bs**, related to **Scheme 2**

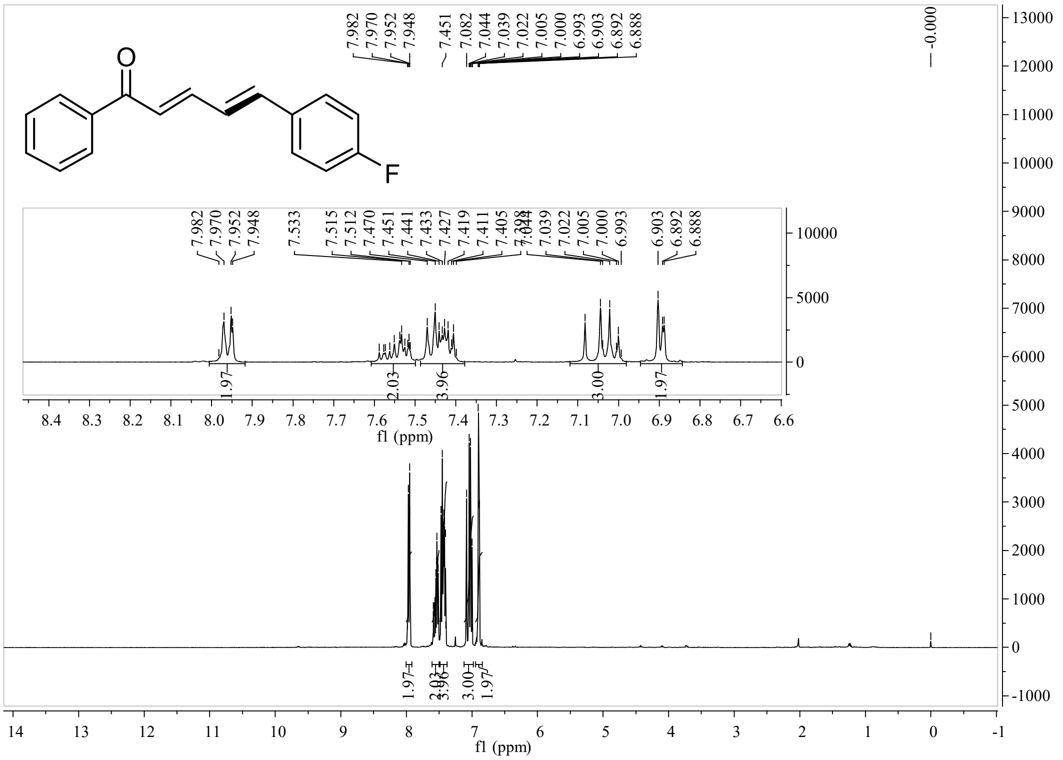

Figure S90 ^{1}H NMR spectrum of compound **2bt**, related to **Scheme 2**

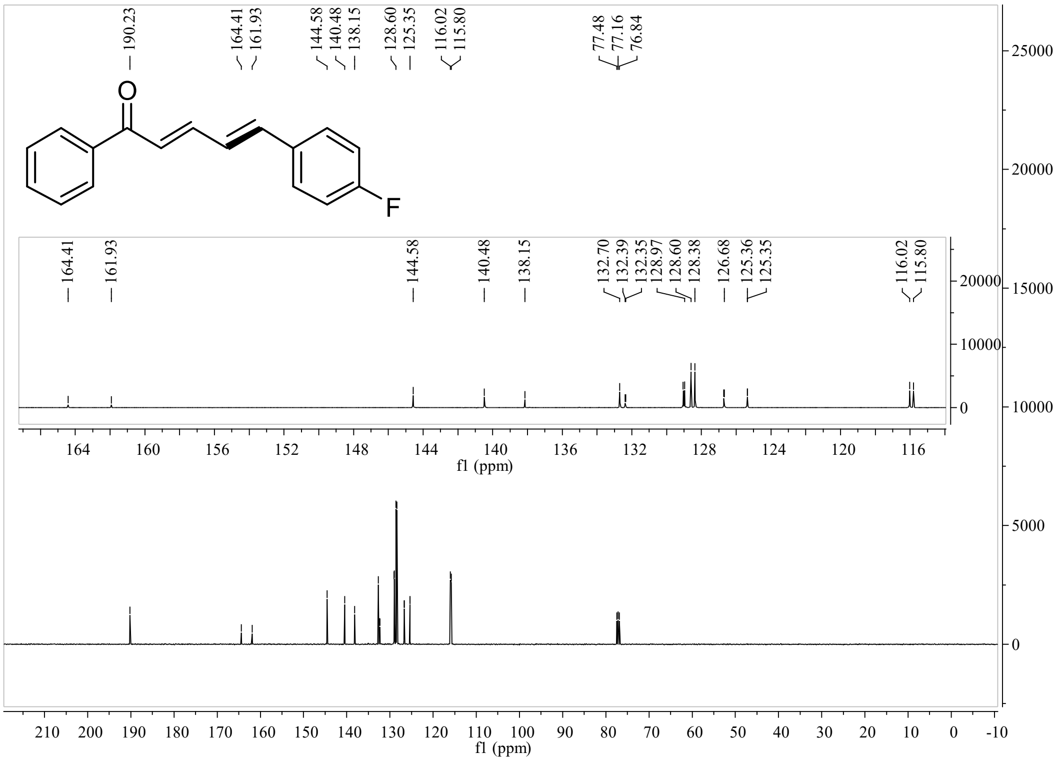

Figure S91 ^{13}C NMR spectrum of compound **2bt**, related to **Scheme 2**

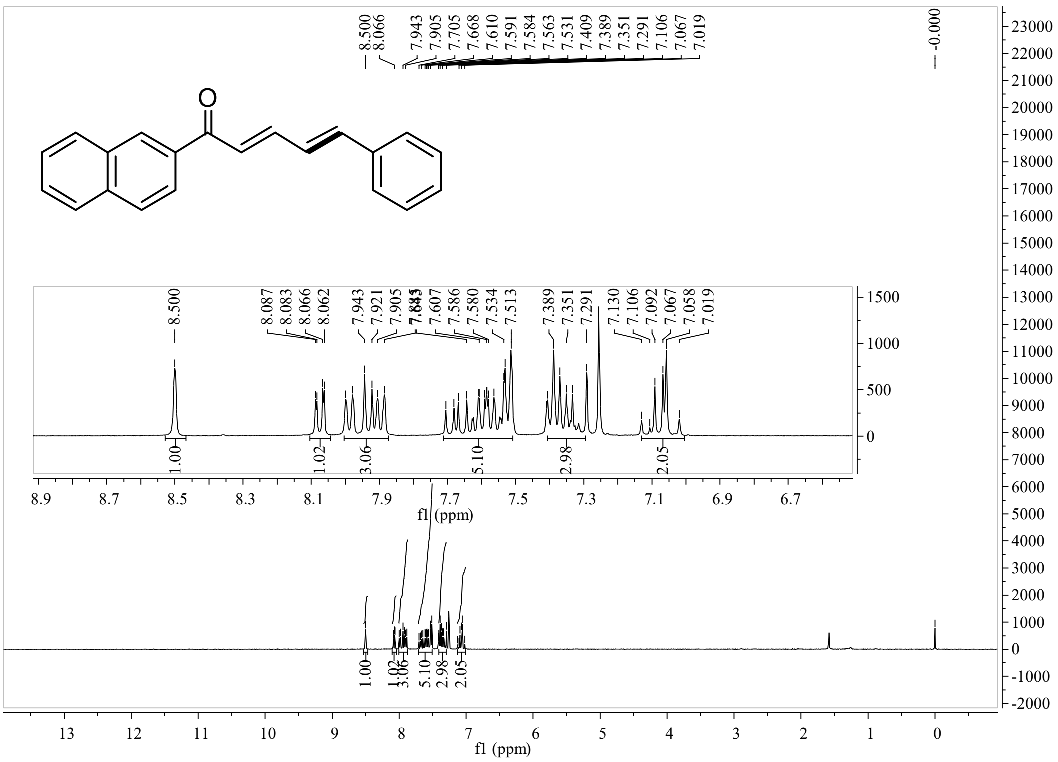

Figure S92 ^{1}H NMR spectrum of compound **2bu**, related to **Scheme 2**

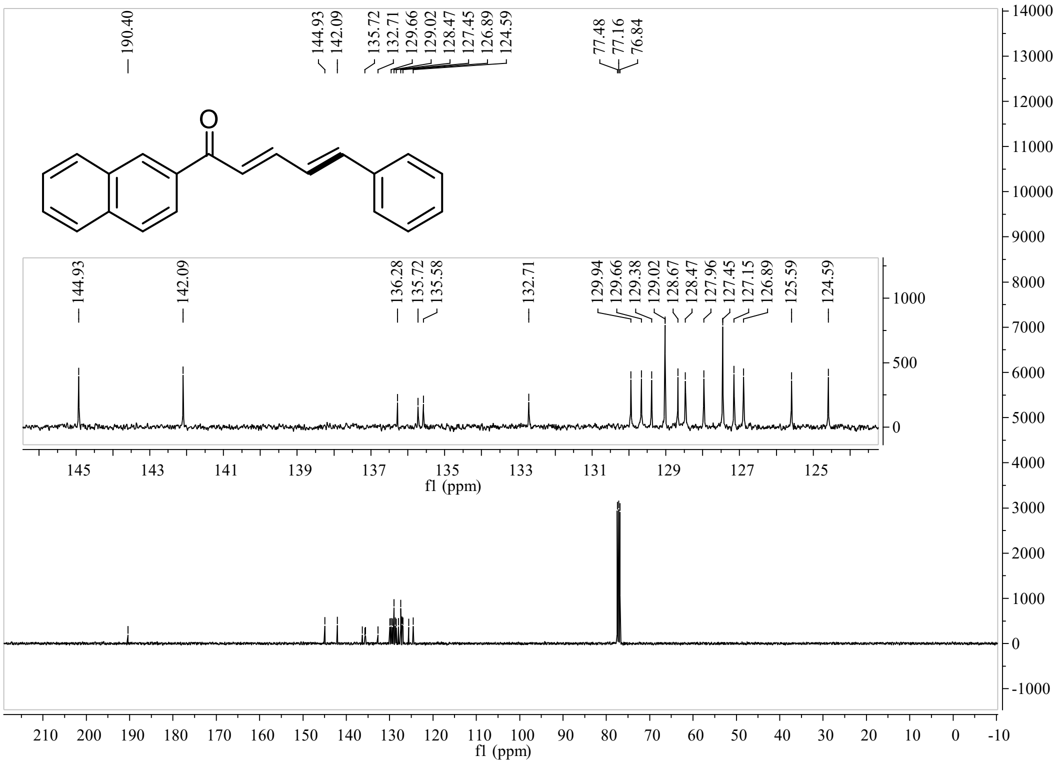

Figure S93 ^{13}C NMR spectrum of compound **2bu**, related to **Scheme 2**

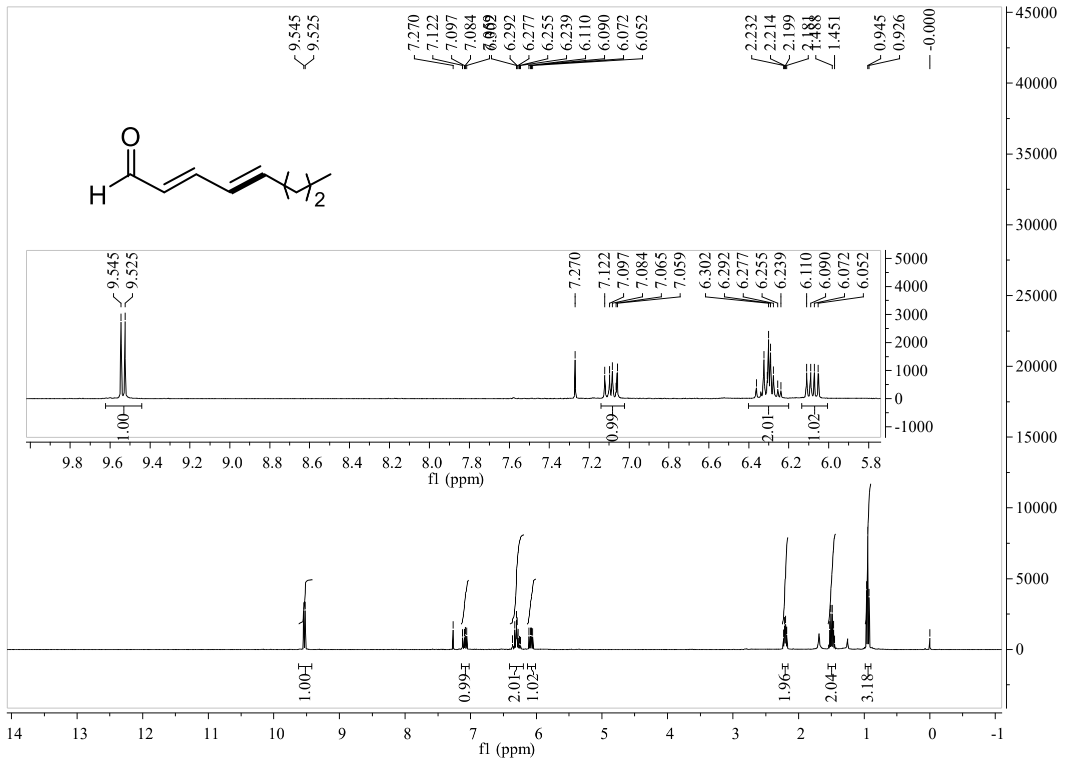

Figure S94 ^{1}H NMR spectrum of compound **4aa**, related to **Scheme 2**

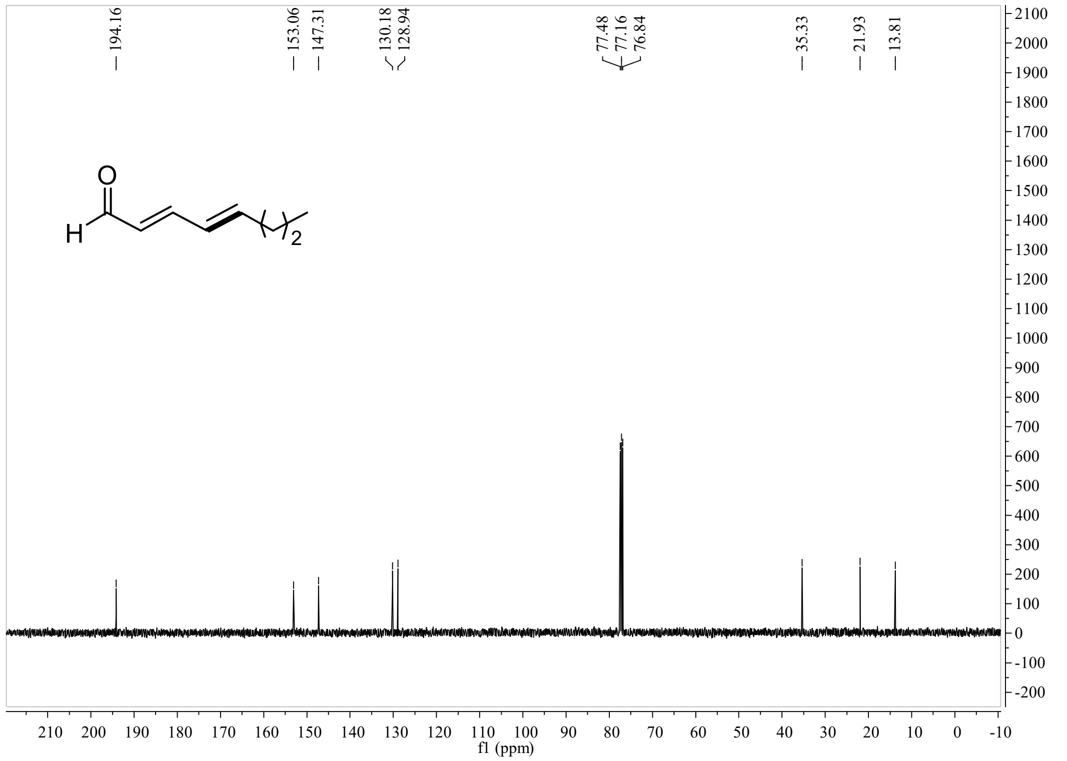

Figure S95 ^{13}C NMR spectrum of compound **4aa**, related to **Scheme 2**

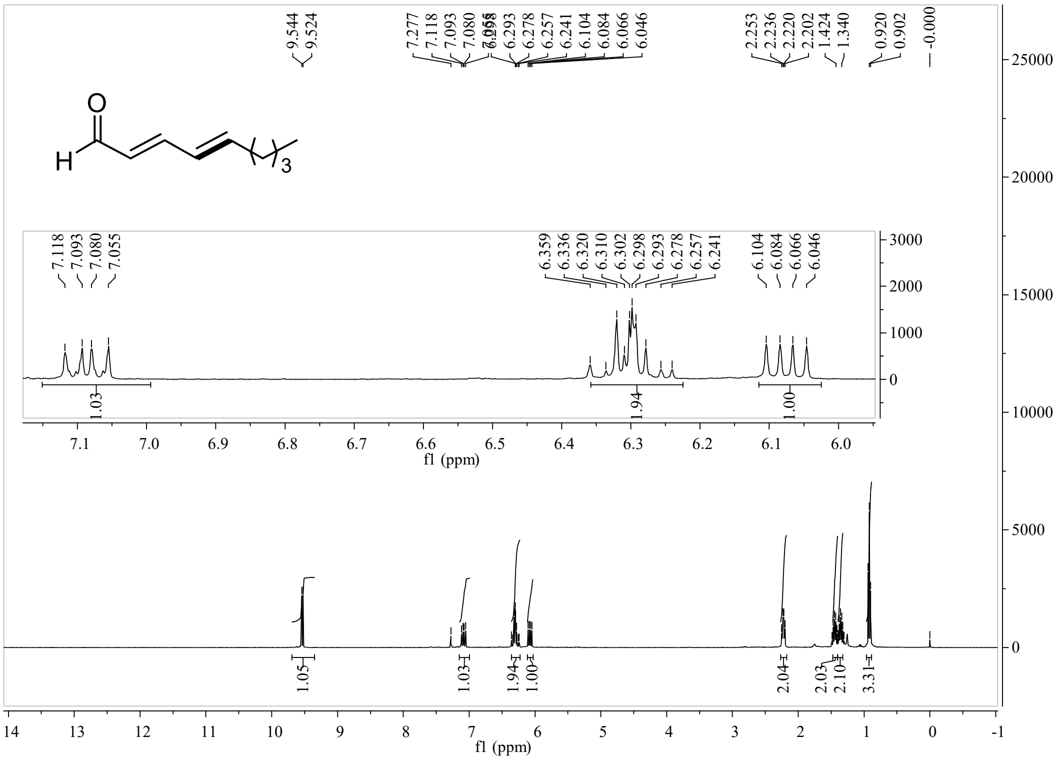

Figure S96 ^{1}H NMR spectrum of compound **4ab**, related to **Scheme 2**

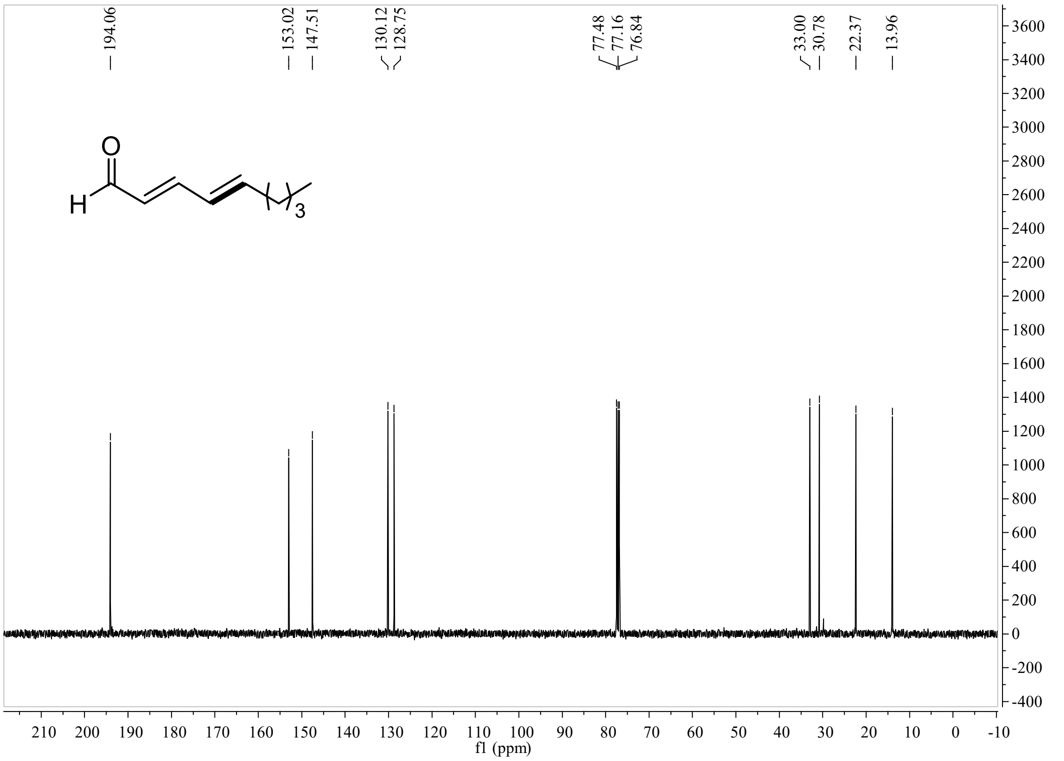

Figure S97 ^{13}C NMR spectrum of compound **4ab**, related to **Scheme 2**

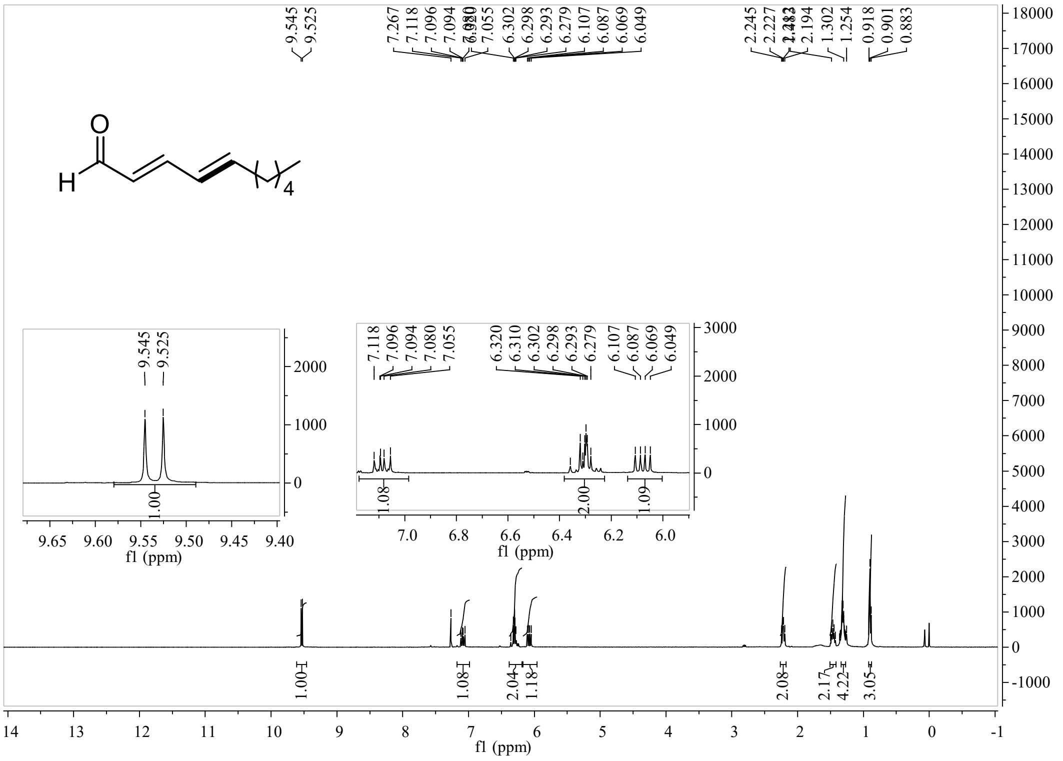

Figure S98 ^{1}H NMR spectrum of compound **4ac**, related to **Scheme 2**

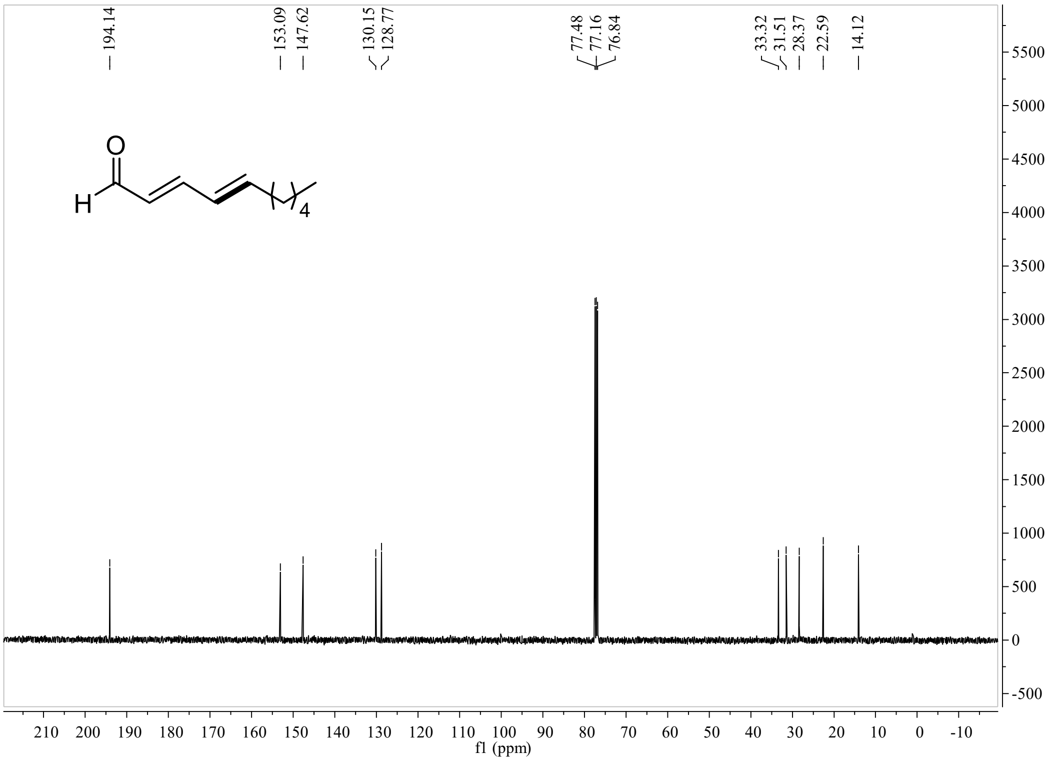

Figure S99 ^{13}C NMR spectrum of compound **4ac**, related to **Scheme 2**

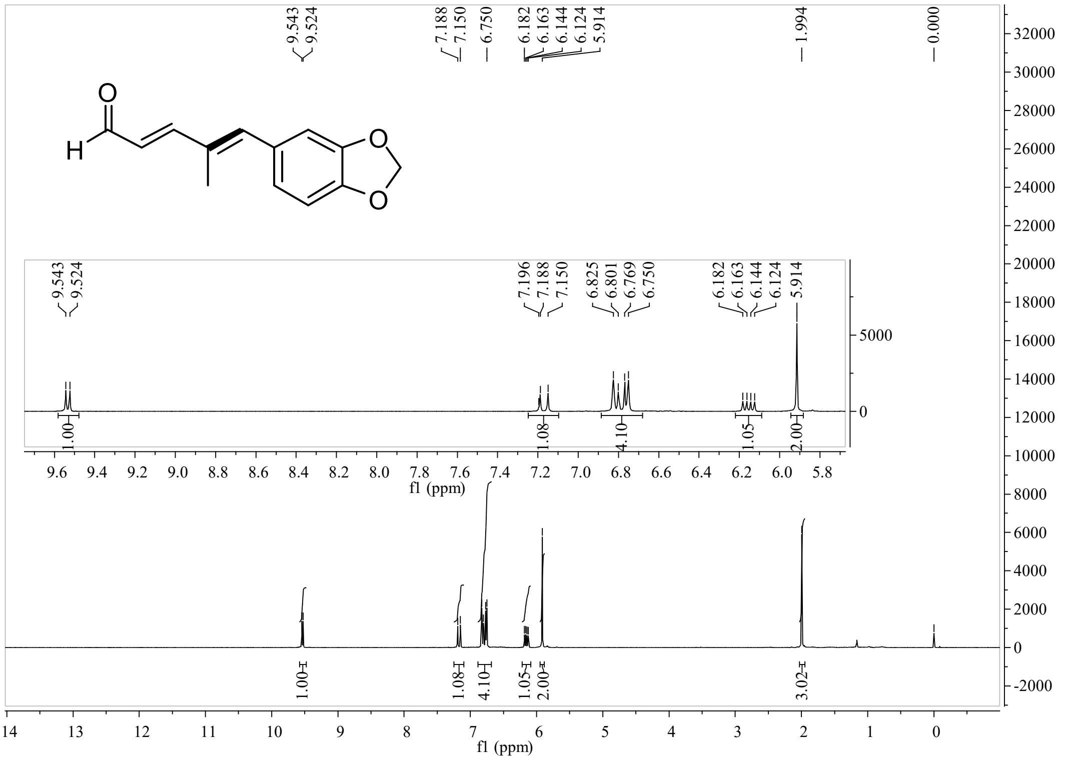

Figure S100 ^{1}H NMR spectrum of compound **4ae**, related to **Scheme 2**

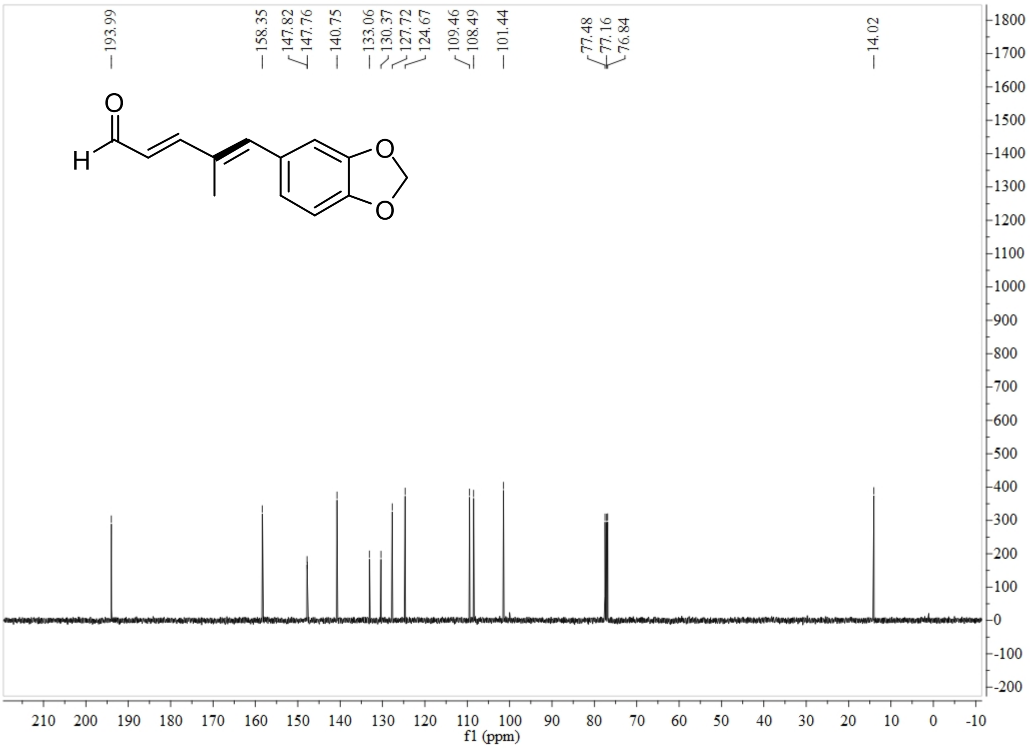

Figure S101 ^{13}C NMR spectrum of compound **4ae**, related to **Scheme 2**

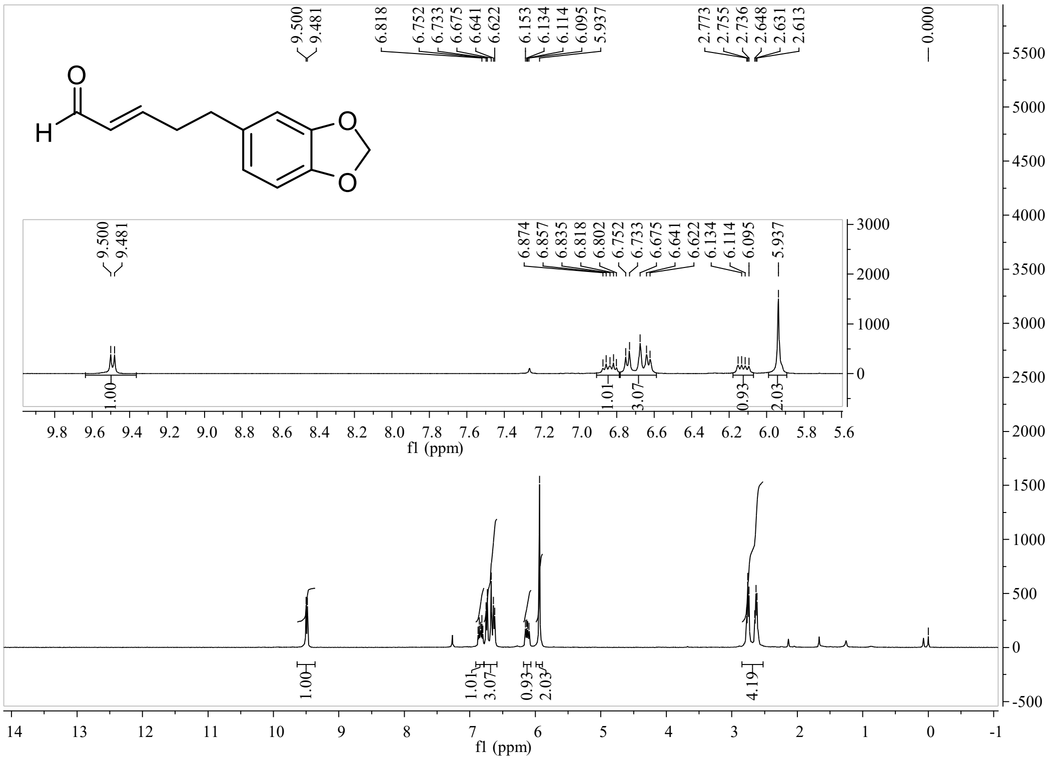

Figure S102 ^{1}H NMR spectrum of compound **3ad**, related to **Scheme 3**

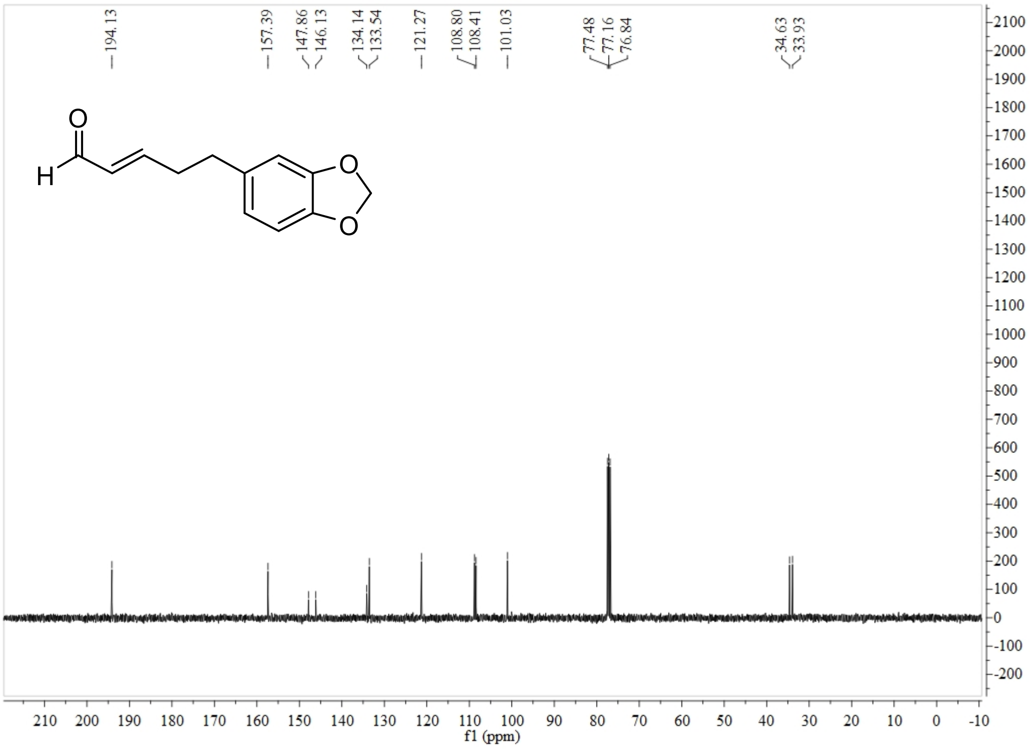

Figure S103 ^{13}C NMR spectrum of compound **3ad**, related to **Scheme 3**

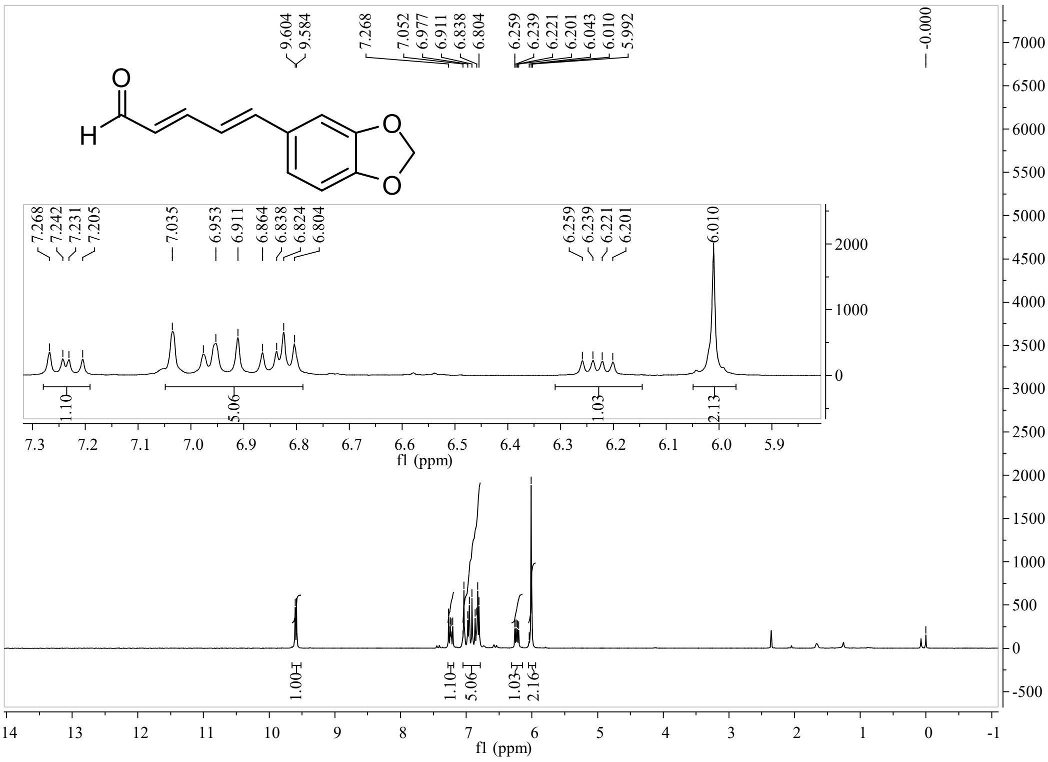

Figure S104 ^{1}H NMR spectrum of compound **4ad**, related to **Scheme 3**

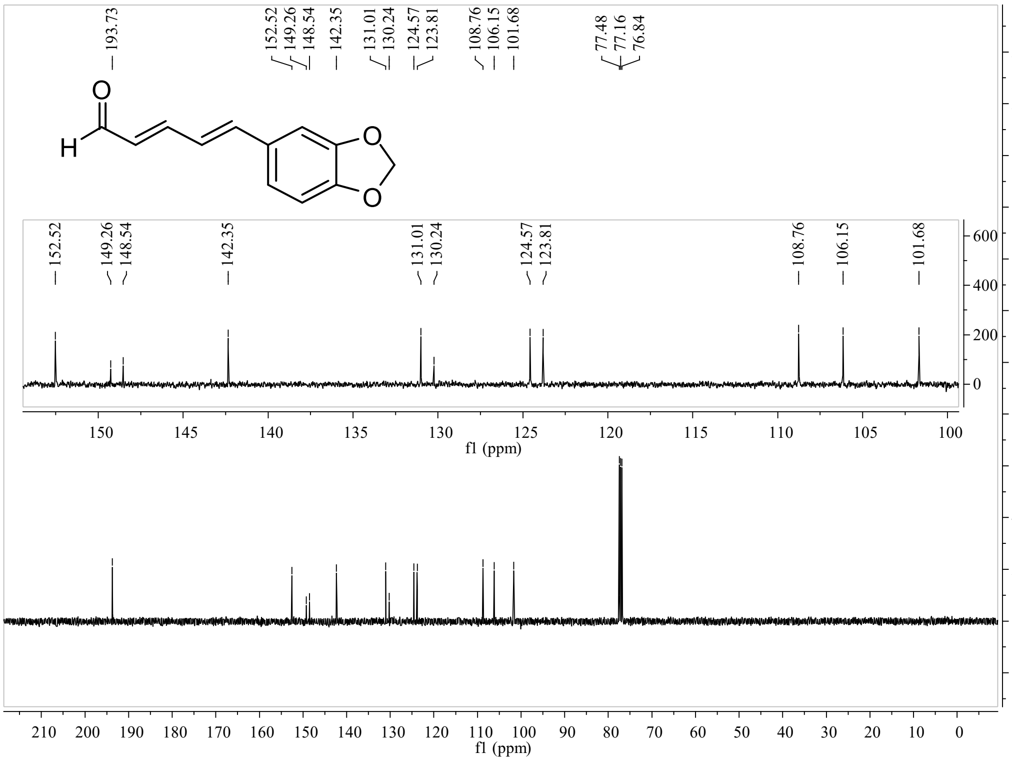

Figure S105 ^{13}C NMR spectrum of compound **4ad**, related to **Scheme 3**

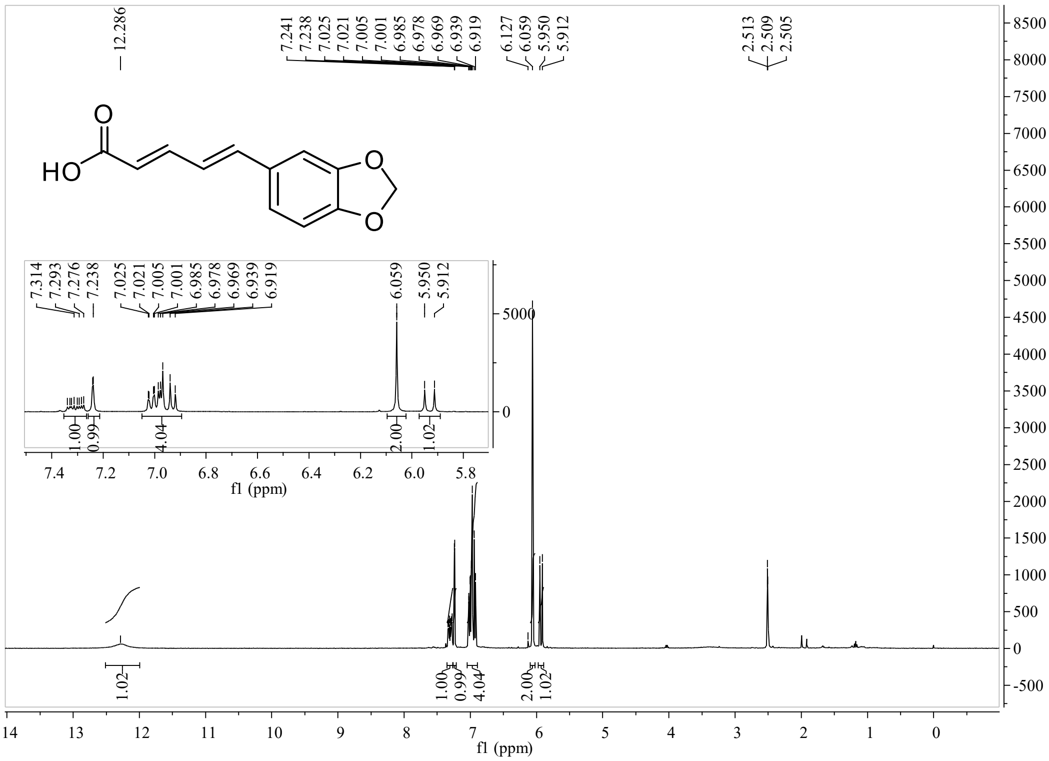

Figure S106 ^{1}H NMR spectrum of compound **5**, related to **Scheme 3**

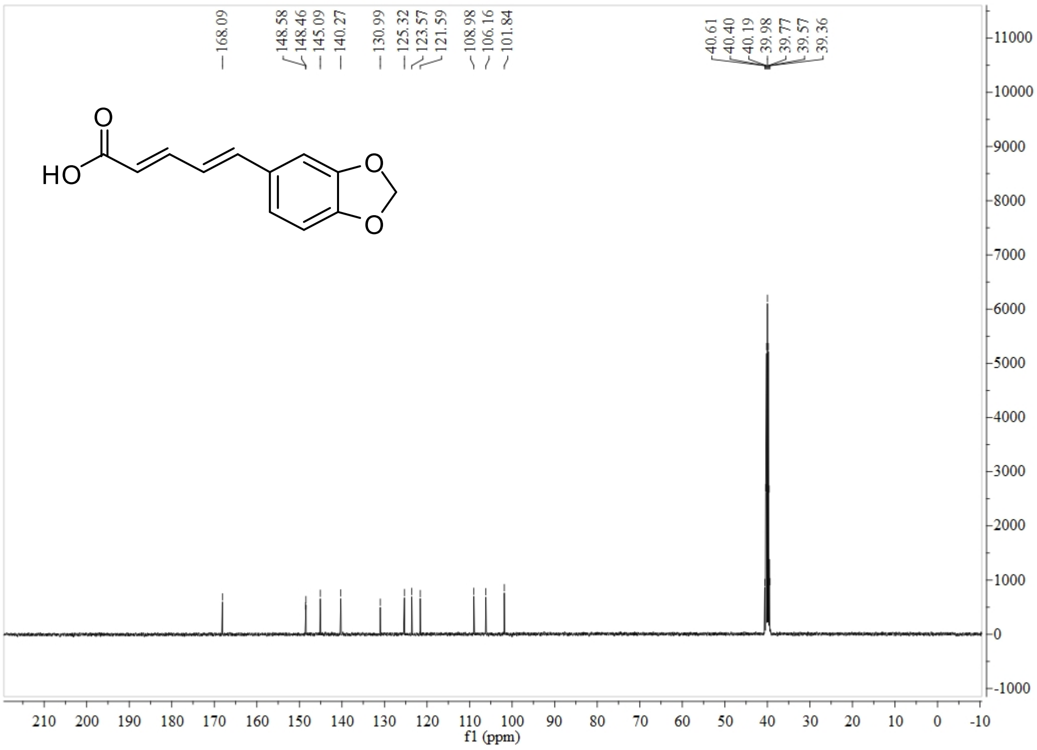

Figure S107 ^{13}C NMR spectrum of compound **5**, related to **Scheme 3**

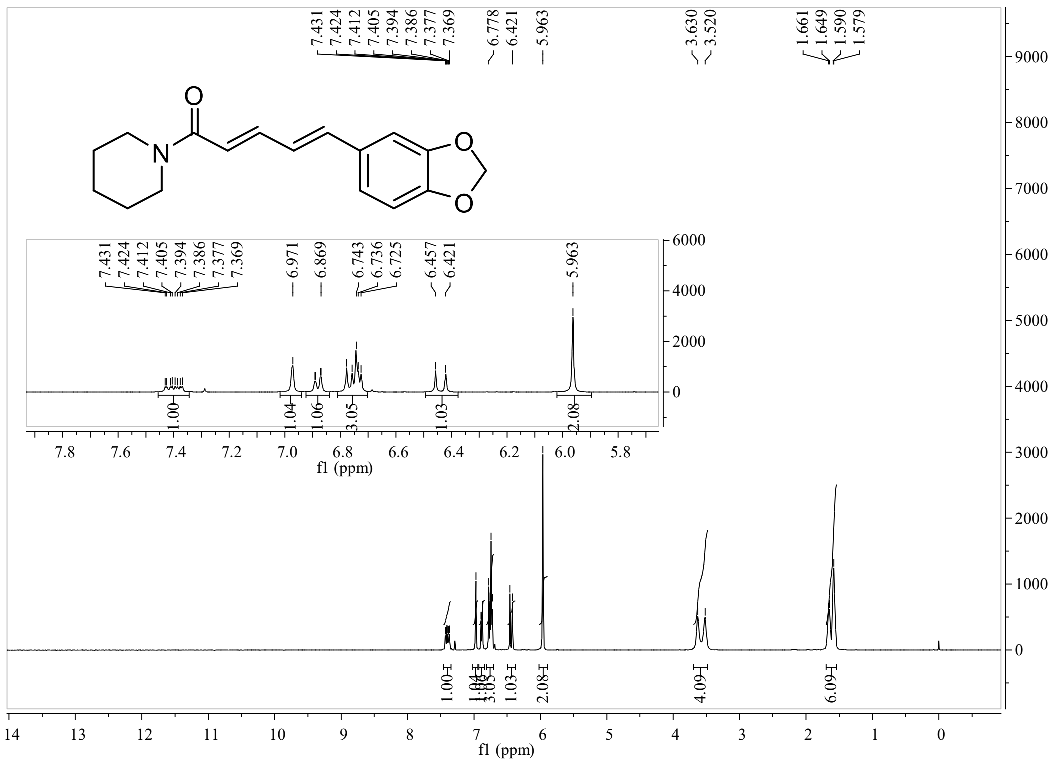

Figure S108 ^{1}H NMR spectrum of **Piperine**, related to **Scheme 3**

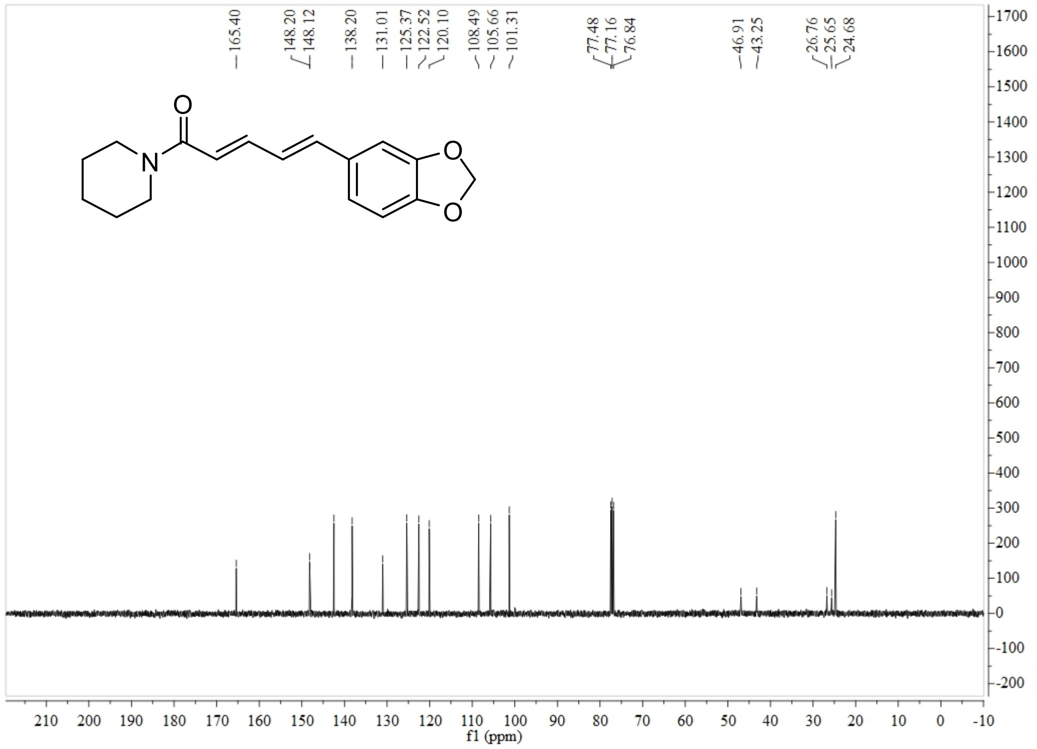

Figure S109 ^{13}C NMR spectrum of **Piperine**, related to **Scheme 3**

6. X-ray Crystal Structure of Compounds 2bt

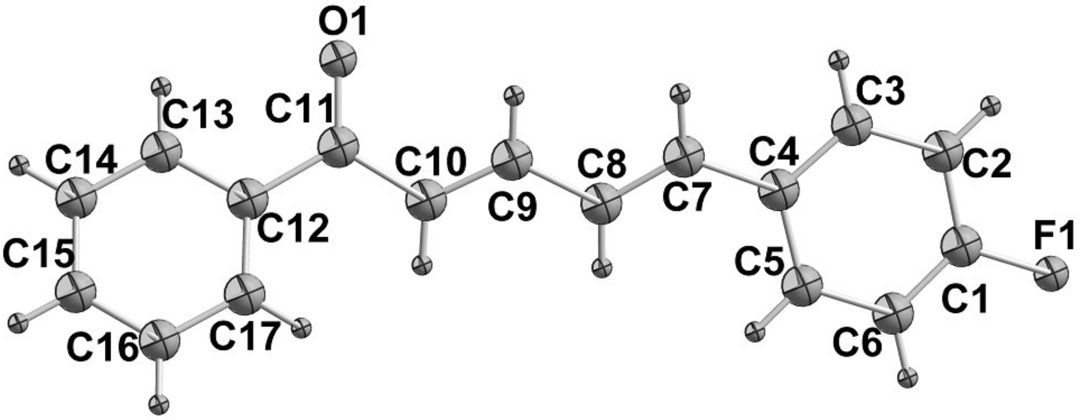

Figure S110 X-ray Crystal Structure of compound **2bt**, related to **Scheme 2**
